# Machine Learning-Based Integrated Analysis of PANoptosis Patterns in Acute Myeloid Leukemia Reveals a Signature Predicting Survival and Immunotherapy

**DOI:** 10.1155/2024/5113990

**Published:** 2024-01-30

**Authors:** Lanlan Tang, Wei Zhang, Yang Zhang, Wenjun Deng, Mingyi Zhao

**Affiliations:** ^1^Department of Pediatrics, The Third Xiangya Hospital, Central South University, Changsha, Hunan 410013, China; ^2^Department of Pediatrics, Xiangya Hospital, Central South University, Changsha, Hunan 410008, China

## Abstract

**Objective:**

We conducted a meticulous bioinformatics analysis leveraging expression data of 226 PANRGs obtained from previous studies, as well as clinical data from AML patients derived from the HOVON database.

**Methods:**

Through meticulous data analysis and manipulation, we were able to categorize AML cases into two distinct PANRG clusters and subsequently identify differentially expressed genes (PRDEGs) with prognostic significance. Furthermore, we organized the patient data into two corresponding gene clusters, allowing us to investigate the intricate relationship between the risk score, patient prognosis, and the immune landscape.

**Results:**

Our findings disclosed significant associations between the identified PANRGs, gene clusters, patient survival, immune system, and cancer-related biological processes and pathways. Importantly, we successfully constructed a prognostic signature comprising nineteen genes, enabling the stratification of patients into high-risk and low-risk groups based on individually calculated risk scores. Furthermore, we developed a robust and practical nomogram model, integrating the risk score and other pertinent clinical features, to facilitate accurate patient survival prediction. Our comprehensive analysis demonstrated that the high-risk group exhibited notably worse prognosis, with the risk score proving to be significantly correlated with infiltration of most immune cells. The qRT-PCR results revealed significant differential expression patterns of LGR5 and VSIG4 in normal and human leukemia cell lines (HL-60 and MV-4-11).

**Conclusions:**

Our findings underscore the potential utility of PANoptosis-based molecular clustering and prognostic signatures as predictive tools for assessing patient survival in AML.

## 1. Introduction

Acute myeloid leukemia (AML) is a heterogeneous disease characterized by a broad spectrum of cytogenetic and molecular abnormalities [1]. While the survival rate for pediatric AML in high-income countries has reached 70%, it remains below 60% in low-income countries [2]. A significant challenge in AML treatment is the occurrence of relapses [3]. The development of inhibitors targeting specific cell death pathways has revolutionized the management of both newly diagnosed and relapsed/refractory AML. Conventional chemotherapeutics primarily induce necroptosis and have been the mainstay of AML treatment. However, emerging evidence suggests promising roles for inhibitors targeting apoptosis, such as venetoclax, as well as molecules associated with pyroptosis, in the treatment of AML [4, 5]. Pediatric clinicians are currently faced with the task of developing a diverse range of novel antileukemia agents to overcome these challenges.

Programmed cell death mechanisms play pivotal roles in the pathogenesis of tumors and the development of chemotherapy resistance [6, 7]. PANoptosis, a novel and intricate programmed cell death pathway, exhibits the unique ability to concurrently trigger and orchestrate three distinct forms of cell death: pyroptosis, apoptosis, and necroptosis [8]. Notably, singular inhibitors of cell death are ineffective against PANoptosis [9]. The formation of a multiprotein complex, known as the PANoptosome, serves as a critical initiator of PANoptosis [10]. The PANoptosome facilitates extensive interplay and coordinated regulation between pyroptosis, apoptosis, and necroptosis. Perturbations in key PANoptotic proteins within cancer cells have been observed, potentially contributing to their survival and resistance against conventional therapies. For example, in colorectal cancer, phosphorylated cysteine desulfurase (NFS1) has been demonstrated to impair the chemosensitivity of oxaliplatin-based treatments through the inhibition of PANoptosis [11]. Furthermore, targeting PANoptosis-induced cardiomyocyte death holds promise as a therapeutic strategy to mitigate doxorubicin-induced cardiotoxicity [12]. Nevertheless, the underlying mechanisms that underpin the association between PANoptosis and AML remain largely obscure. Therefore, unraveling the involvement of PANoptosis in AML could potentially unveil novel therapeutic approaches and enhance the prognosis for pediatric AML patients.

An investigation was conducted in our study to construct a prognostic PANoptosis-related risk score signature, designated as PAN2RS, which comprised 19 genes associated with PANoptosis. This signature was based on the analysis of transcriptional and clinical data obtained from AML patients in the HOVON database. To further elucidate the functionality and prognostic significance of differentially expressed genes, multiple bioinformatics analyses were performed. Additionally, the prognostic value of the PAN2RS signature was validated in independent cohorts, including the Cancer Genome Atlas (TCGA), GEO, and BeatAML databases. Furthermore, in vitro experiments were conducted to assess the expression levels of the 19 genes in AML cell lines. Our findings provide a comprehensive theoretical framework for the identification of novel therapeutic targets and prognostic biomarkers for AML.

## 2. Materials and Methods

### 2.1. Collection and Preprocessing of AML Patient Cohorts and PANoptosis Gene List

All the AML patient cohorts including the gene expression data and the clinical traits were gathered from the public databases, such as the TCGA database, GEO database, and ArrayExpress database. The HOVON cohort was collected from the ArrayExpress database (E-MTAB-3444, https://www.ebi.ac.uk/biostudies/arrayexpress). The clinical characteristics, RNA-seq data, methylation data, and somatic mutation data of the TCGA-LAML cohort were obtained from the UCSC Xena website (https://xenabrowser.net/datapages/). GSE37642-GPL96 (*n* = 422), GSE12417-GPL96 (*n* = 163), and GSE106291 (*n* = 250) were gathered from the Gene Expression Omnibus (GEO, https://gdc.cancer.gov/access-data/gdc-data-portal). The BeatAML cohort was gathered from the GDC data portal (https://gdc.cancer.gov/access-data/gdc-data-portal). The AML patients with completive survival information and the survival time >0 were retained. In BeatAML cohort, the AML patients from the therapy-naïve adults were reserved. Finally, we got six AML patient cohorts including HOVON (*n* = 618), TCGA-LAML (*n* = 147), BeatAML (*n* = 143), GSE37642-GPL96 (*n* = 421), GSE12417-GPL96 (*n* = 162), and GSE106291 (*n* = 250). Considering the size of the dataset, the HOVON cohort was used as the training set and the other cohorts were used as the testing set. All relevant RNA-sequencing data were normalized with the R package limma and the raw count was transformed into log2 (the transcripts per million (TPM) + 1), which was usually used to measure the gene expression level. And for the expression data from microarrays, background adjustment and normalization were performed with R package affy.

The PANoptosis-related genes were combination of the pyroptosis-related genes, apoptosis-related genes, and necroptosis-related genes. All of them were obtained from the Molecular Signatures Database (MSigDB, https://www.gsea-msigdb.org/gsea/msigdb). The pyroptosis and apoptosis gene lists were gathered from the Reactome Pathway Database with searching for the keywords “pyroptosis” and “apoptosis,” respectively. The necroptosis gene list was collected from the Gene Ontology database with searching for the words “GOBP_PROGRAMMED_NECROTIC_CELL_DEATH.” The three gene lists were merged and the overlapping genes were excluded, forming the final PANoptosis gene list (*n* = 226), the detailed information of which is summarized in Supplementary Table 1.

### 2.2. Consensus Clustering Analysis

In order to identify the transcriptome characterization of the PANoptosis-related patterns, the consensus clustering analysis with the “pam” algorithm and Euclidean correlation distance was performed with the R package “ConsensusClusterPlus.” Additionally, the clustering algorithm with resampling 80% of the samples was repeated for 1000 times. The principal component analysis (PCA) was adopted to investigate the distribution of the PANoptosis patterns.

### 2.3. Immune-Related Analysis

Here, to compare immunization between the various subgroups, we performed multiple methods associated with the immunity analysis including the expression of the immune check point gene, antitumor immunity cycle, infiltration of immune cells, objective response rate (ORR), and so on. The 75 immune check points belong to 7 groups, such as antigen presentation, cell adhesion, coinhibitor, costimulator, ligand, receptor, and others. The antitumor cycle including 7 steps and corresponding gene signatures are also shown in Supplementary Table 2. To more accurately describe the infiltration of immune cells in the tumor microenvironment, we had adopted 7 common approaches, consisting of ESTIMATE, xCell, quanTIseq, ssGSEA, Cibersort, EPIC, and MCP-counter. Those algorithms were carried out with the R packages including GSVA, CIBERSORT, ESTIMATE, and immunedeconv. The ORR of anti-PD-1/PD-L1 therapy across the cancers in TCGA was obtained from the previous literature and is summarized in Supplementary Table 3.

### 2.4. Pathway Enrichment Analysis

For the up and down expressed DEGs, the Gene Ontology (GO) and Kyoto Encyclopedia of Genes and Genomes (KEGG) pathway enrichment analyses were performed to explore the potential biological function, respectively. Also, to ensure the reliability of the enrichment analysis results, based on the gene sets, other algorithms such as Gene Set Enrichment Analysis (GSEA) and Gene Set Variation Analysis (GSVA) were carried out with the R package GSVA and clusterProfiler. The four gene sets including REACTOME, KEGG, GO-BP, and HALLMARK were downloaded from MSigDB.

### 2.5. Machine Learning Framework for Development of PANoptosis Signature

Although the features of PANoptosis patterns were identified, the limitation of using the all the transcriptome profile was not convenient to accurately and clinically predict the prognosis of each patient. So, it was urgent to develop a relatively streamlined but powerfully predictive model. (a) Based on the different PANoptosis patterns, the differently expressed gene (DEG) analysis was subsequently carried out with the R package limma between the PANoptosis cluster with the worst prognosis and the PANoptosis cluster with the best prognosis. In order to retain as many genes as possible for the subsequent selection, the DEGs were screened out with FDR <0.05. (b) If a gene was robustly expressed in the cohort and had a small range of fluctuations in expression, then we preferred to consider the gene to be of low importance. So, we therefore excluded DEGs with a variance less than or equal to 0.3 in HOVON cohort. (c) To screen for the DEGs with the predictive efficacy, two different strategies were consecutively executed. Firstly, the univariate Cox regression analysis was performed for the DEGs in HOVON and the DEGs with the *p* value less than 0.01 were identified as the significantly prognostic DEGs. (d) Subsequently, we categorized the patients in the HOVON cohort into two subgroups based on the expression levels of the prognostic DEGs identified above, namely, the subgroup with high gene expression and the subgroup with low gene expression. The division was made using the median expression value as a threshold. Then, we performed a log-rank test to compare the overall survival time between these two subgroups and calculated the statistical significance of the differences. Finally, DEGs with a p value less than 0.05 were designated as the prognostic DEGs. The DEGs with *p* value less than 0.05 were finally identified as the prognostic DEGs. (e) To develop a highly robust and accurate PANoptosis signature (PANS), we further fitted the 101 predictive models with the candidate prognostic DEGs through the leave-one-out cross-validation (LOOCV) framework, consisting of 101 combinations of 10 machine learning algorithms including least absolute shrinkage and selection operator (lasso), elastic network (Enet), Ridge, supervised principal components (SuperPC), StepCox, Gradient Boosting Machine (GBM), CoxBoost, partial least squares regression for Cox (plsRcox), Random Survival Forest (RSF), and Survival Support Vector Analysis (SurvivalSVM). The four cohorts (HOVON, TCGA-LAML, GSE12417-GPL96, and GSE37642-GPL96) were detected, and Harrell's concordance index (C-index) of each predictive model was calculated across all cohorts. The optimal and best model was the model with the highest mean of the C-index in all detected cohorts. (f) Random Survival Forest (RSF), which was a machine learning and nonparametric algorithm that could handle high-dimensional data and complex interactions between the variables, was then performed to screen the candidate genes which were the most important features related with prognosis. Specifically, the function “var.select” in the R package randomForestSRC was utilized to help select the candidate genes and interpret the contribution of each candidate gene to the model prediction. With the multivariate Cox regression analysis in HOVON cohort, the coefficients of the candidate genes were calculated. So ultimately, we developed a PANoptosis signature scoring system, the formula of which was as follows:

(1) $$\begin{array}{c} \text{Risk Score}=\overset{N}{\underset{i=1}{\sum }}\left(\text{Expi}*\text{Coei}\right), \end{array}$$

where *N*, Exp, and Coe represented the number, gene expression, and the corresponding coefficient of the candidate genes, respectively. The risk score of the patients in all cohorts was calculated with the above formula.

### 2.6. Evaluation and Validation of the Developed PANoptosis Signature

In order to comprehensively assess the developed PANoptosis scoring system, the PANoptosis-related signature risk scores (PAN2RSs) were first calculated for each patient in all cohorts based on the profile of the PANoptosis signature and the corresponding coefficients. And with the optimal cutoff value determined by the R package survminer, the patients in each cohort were divided into two subgroups, named high PAN2RS group and low PAN2RS group. The Kaplan–Meier survival analysis was performed in the training cohort and the testing cohorts with the R package survival and the log-rank test. The time-dependent ROC analysis was also carried out in all cohorts, through the R package survivalROC and with the 1-year, 3-year, and 5-year area under curve (AUC) calculated. Moreover, the C-index and the 95% confidence interval of it were calculated in all cohorts with the R package “survcomp.” To evaluate the robustness and the independence of the signature, the multivariate Cox regression analysis was adopted based on the clinical information and the PAN2RS in HOVON cohort and TCGA-LAML cohort through the function coxph in R package survival. In order to systematically synthesize and analyze the existing studies and thus yield more accurate and objective conclusions, the meta-analysis was carried out to investigate the HR of the all six cohorts for the PANoptosis signature, through the R package meta.

### 2.7. Comparison of the PANoptosis Signature and the Previous Signatures

In order to assess the superiority of the predictive performance of the developed PANoptosis signature, we retrospectively collected 89 AML-related signatures and their corresponding coefficients from the published literature [13, 14], which is detailed and summarized in Supplementary Table 4. The patients in all cohorts were scored based on the gathered signatures and then the 1-year, 3-year, and 5-year AUC and the C-index were computed in the six cohorts. And with the mean value of the indicators across the six cohorts, we compared the predictive performance of each signature. Furthermore, with the risk scores calculated by 90 signatures and the clinical information, the multivariate Cox regression analysis was performed in HOVON cohort and TCGA-LAML cohort, and the *p* value of the risk score for each signature was computed and compared to show the independence and the robustness.

### 2.8. Construction of the PANoptosis-Related Nomogram

In order to improve clinical guidance and precision medicine for patients, including the accurate assessment of patient risk and the selection of individualized treatment strategies, a nomogram was established based on the common clinical variables (age, gender, cytogenetic risk, NPM1 mutation, and FLT3-ITD mutation) and the PAN2RS through the R package “rms.” To better visualize the discrimination and the prediction of the value for the developed nomogram, the curves of 1-year, 3-year, and 5-year calibration were drawn.

### 2.9. Prediction of the Response to the Immunotherapy for PANoptosis Signature

The datasets of Braun_2020 (renal cell carcinoma), PRJEB23709 (melanoma), phs000452 (melanoma), Nathanson_2017 (melanoma), GSE106128 (melanoma), GSE100797 (melanoma), GSE91061 (melanoma), GSE78220 (melanoma), the IMvigor210 (bladder cancer), and PRJNA482620 (glioblastoma) were collected and utilized to describe the prediction of response to immunotherapy. The core information of the immunotherapy datasets is summarized in Supplementary Table 5, and the PAN2RS was computed in the ten cohorts based on the PANoptosis signature, respectively.

### 2.10. Prediction of the Potential Drug Targets and Prospective Therapeutic Agents

Transcriptome data of human cancer cell lines (CCLs) were downloaded from the Broad Institute-Cancer Cell Line Encyclopedia project (CCLE, https://sites.broadinstitute.org/ccle/) and the CERES scores, the score to evaluate the dependency of the certain gene in the CCL, which indicated that the score was negative correlation with the possible significance of the gene in cell proliferation of the certain CCL, were downloaded from the dependency map portal (DepMap, https://depmap.org/portal/). For the CERES scores of the 18333 genes and 739 cell lines, we selected the leukemia-associated cell lines and scored them based on the PANoptosis signature. For each drug target, Spearman's rank-order correlation of the CERES score and the PAN2RS based on the leukemia-related cell lines was calculated. According to the computed correlation, the potential drug targets were further identified (*r* < −0.45 and *p* value <0.05).

In order to investigate the ex vivo drug sensitivity, we collected the transcriptome data and corresponding drug sensitivity assay data including the IC50 and the AUC of the 122 small-molecule inhibitors from the previous literature. After excluding objects with more than 50% missing data, there remained a total of 337 patients and 106 inhibitors in the dataset. The PAN2RS of each patient in the cohort was computed based on the PANoptosis signature. Additionally, in order to identify the sensitive agents for the PAN2RS, not only the differential analysis between AUC of the high PAN2RS patients (top decile) and the low PAN2RS patients (tail decile) (Wilcoxon test, R package stats) but also the correlation of the PAN2RS and the AUC of the patients (top and tail deciles) (Spearman's rank-order correlation, R package stats) were performed. We further ensured the prospective and sensitive agents for the high PAN2RS AML patients with the correlation's *r* < 0, correlation's *p* value <0.05, and the *p* value of the Wilcoxon test <0.05.

Acquired from the Cancer Therapeutics Response Portal (CTRP, https://portals.broadinstitute.org/ctrp.v2.1/) and PRISM repurposing dataset (https://www.theprismlab.org/), the agent sensitivity data of CCLs including the AUC values which indicated the drug sensitivity were utilized to find the potential drugs. After getting rid of the agents with more than 20% of missing information, the missing values of the AUC were extrapolated with the K-nearest neighbor imputation method (R package impute) in the both CTRP and PRISM datasets. The Ridge regression with the R package pRRophetic was carried out to develop a model for drug sensitivity prediction based on the transcriptome data and the drug sensitivity data in CTRP and PRISM. Based on the drug sensitivity model and the PANoptosis signature, we scored the AUC values of drugs and the PAN2RS of samples in the 337-patient cohort mentioned above, respectively. Similar to the above, once the 337 patients had been sorted according to PAN2RS, the top and bottom decile of patients had been extracted, and then the differential drug response analysis (Wilcoxon rank sum test) and the correlation of PAN2RS and AUC (Spearman rank correlation) had been used to identify the potential candidate drugs for the poor prognosis AML patients with the overlap of the drugs with lower AUC in the high-risk score group and the drugs with negative correlation (*R* < 0.3 or *R* < 0.4).

Apart from the above methods, with the Connectivity Map (Cmap, https://clue.io/query) dataset including 2424 perturbational drug sensitivity signatures, the Kolmogorov–Smirnov (KS) scores and eXtreme Sum (XSum) scores were computed based on the 300 differently expressed genes between the top risk decile and the tail risk decile of patients in the 337-patient cohort. A drug with the score closer to −1 (mapping transformed) or −100 was more likely to be a drug that reverses a poor prognosis for the high PAN2RS AML patients.

### 2.11. Pan-Cancer Analysis of Prognosis Prediction, Immune Microenvironment, and Somatic Mutation

To investigate the ubiquitous applicability of this model across diverse tumors, the pan-cancer analysis was performed based on the pan-cancer data including 33 different cancers in TCGA, which were acquired from the UCSC Xena database (https://xenabrowser.net/datapages/). Excluding the patients without the survival information, 10295 patients whose overall survival time and OS are at least preserved were retained. We first explored the predictive performance of the PANoptosis signature across the 33 cancers with the univariate Cox regression analysis and the Kaplan–Meier survival analysis. Tumor mutation burden (TMB) varies across the diverse cancers. Since the high values of TMB indicated a prospective response to the immunotherapy [15], we explored the values of TMB and the correlation of the PAN2RS and the TMB values across the cancer types (Spearman rank correlation). Moreover, the somatic mutation and the copy number alterations of the PANoptosis signature genes were also investigated across the cancers with R package maftools. To determine the transcriptome profile of the signature genes in other cancer types, the differential expression analysis between the tumor and the normal was carried out for the identified genes. We also investigated the potential biological functional pathways that the signature genes might involve in with the GSEA algorithm and HALLMARK gene set (R package clusterProfiler). Specifically, for one given cancer type, the patients were sorted based on the expression of the certain gene. The differently expressed analysis was performed between the patients with the top and tail 35% expression of the gene. And then, the GSEA method was carried out based on the result of the differential expression analysis. More importantly, the tumor immunity, including the immune infiltration and the correlation of the immune checkpoint genes and the PAN2RS, was explored across the cancer types, with the R package IOBR.

### 2.12. Cell Culture

HL-60 and MV-4-11 cells (Xiangya School of Medicine Type Culture Collection, China) were cultured in RPMI 1640 medium supplemented with 10% heat-inactivated FBS (Life Technologies, NY, USA) in 5% CO2. HL-60 is a promyelocytic leukemia cell line [16]. MV-4-11 is a FLT3-ITD -positive myelomonocytic leukemia cell line [17]. All cells were incubated at 37°C in a humidified atmosphere of 5% CO2 and 95% air.

### 2.13. Quantitative Real-Time PCR Analysis

The cell samples from AML cell lines were collected in the logarithmic phase of cell growth. Peripheral blood mononuclear cells (PBMCs) were collected from a healthy donor from Xiangya Hospital. Total RNA was extracted by using the Tiangen RNA extraction reagent kit, following the manufacturer's instructions. Subsequently, the extracted RNA samples were reverse transcribed into complementary DNA (cDNA) using a reverse transcription (RT) reagent kit (Dingguo, Beijing, China). Real-time PCR was conducted utilizing SYBR Premix ExTaq (Takara) on a StepOnePlus Real-Time PCR System (Applied Biosystems, Foster City, CA, USA). The obtained data were duly normalized against the *β*-actin internal control. Transcript levels were analyzed using the comparative threshold cycle method. All experiments were repeated in triplicates. The primer sequences for the chosen 19 genes can be found in Supplementary Table 6.

### 2.14. Statistical Analysis

All statistical analyses were carried out with R software (version 4.1.3). The continuous variables and the categorical variables were compared with Wilcoxon rank sum test and chi-square test, respectively. Three or more groups of continuous variables are compared using the Kruskal–Wallis test. The correlation analysis was performed with Spearman's rank-order correlation. The two-sided *p* value <0.05 was considered significant for all statistical analyses and shown as ^*∗*^*p* < 0.05, ^*∗∗*^*p* < 0.01, ^*∗∗∗*^*p* < 0.001, and ^*∗∗∗∗*^*p* < 0.0001. The other detailed methods were summarized in supplementary methods.

## 3. Results

### 3.1. Identification of the Diverse Patterns Based on the PANoptosis Gene List

**Figure 1:**
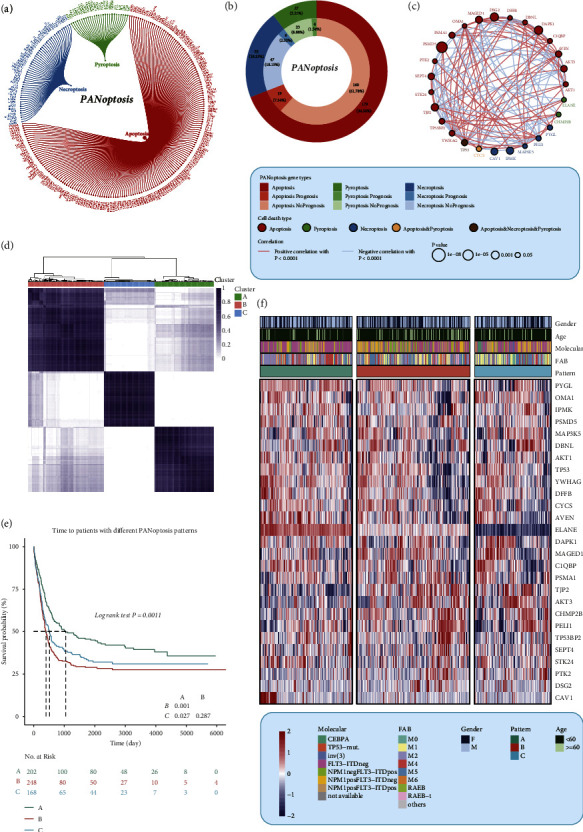
Identification of the diverse patterns based on the PANoptosis gene list. (a) A total of 226 PANoptosis-related genes belonging to necroptosis, apoptosis, and pyroptosis were collected for downstream analysis. (b) The univariate Cox regression analysis and Spearman's sum rank test were performed in the HOVON cohort, which was set as the training cohort. 28 genes with prognostic prediction were screened out with *p* value <0.05. (c) An interaction network of prognostic PANoptosis-related genes. (d) The 618 patients in HOVON cohort were divided into three clusters, according to the consensus clustering analysis based on the transcriptome profile of PANoptosis-related genes. (e) The KM curves showing the differential overall survival among the three subgroups. (f) A heat map showing the expression of the prognostic PANoptosis related genes in different subgroups. The two-sided *p* value <0.05 was considered significant for all statistical analyses and shown as ^*∗*^*p* < 0.05, ^*∗∗*^*p* < 0.01, ^*∗∗∗*^*p* < 0.001, and ^*∗∗∗∗*^*p* < 0.0001.

A total of 226 PANRGs belonging to necroptosis, apoptosis, and pyroptosis were collected for downstream analysis (Figure 1(a)). To explore the prognostic performance and internal relationship of the PANRGs, univariate Cox regression analysis and Spearman's sum rank test were performed in the HOVON cohort, which was set as the training cohort. 28 genes with prognostic prediction were screened out with *p* value <0.05 (Figure 1(b), Supplementary Table 7). The correlation of the predictive PANoptosis genes was also investigated and shown (Figure 1(c)). And then, the 618 patients in HOVON cohort were divided into three clusters, named cluster A (*n* = 202), cluster B (*n* = 248), and cluster C (*n* = 168), according to the consensus clustering analysis based on the transcriptome profile of PANRGs (Figure 1(d), Supplementary Figures 1A–1I). The PCA analysis showed the distribution of the different clusters (Supplementary Figure 1J). Each cluster corresponded to a specific expression pattern of the PANRGs. To investigate whether different expression patterns had an impact on the prognosis of AML patients, Kaplan–Meier survival analysis was applied, and the Kaplan–Meier curves indicated that there was a definite and significant difference in prognosis among the identified patterns (Figure 1(e)). Patients with pattern A had a better prognosis than those with pattern B (*p* < 0.05). Furthermore, the expression of prognostic PANRGs was shown to investigate the difference of each gene expression and the clinical features in different patterns (Figure 1(f)).

### 3.2. Description of the Clinical Traits, Immune Features, and Biological Function for the Diverse Patterns

**Figure 2:**
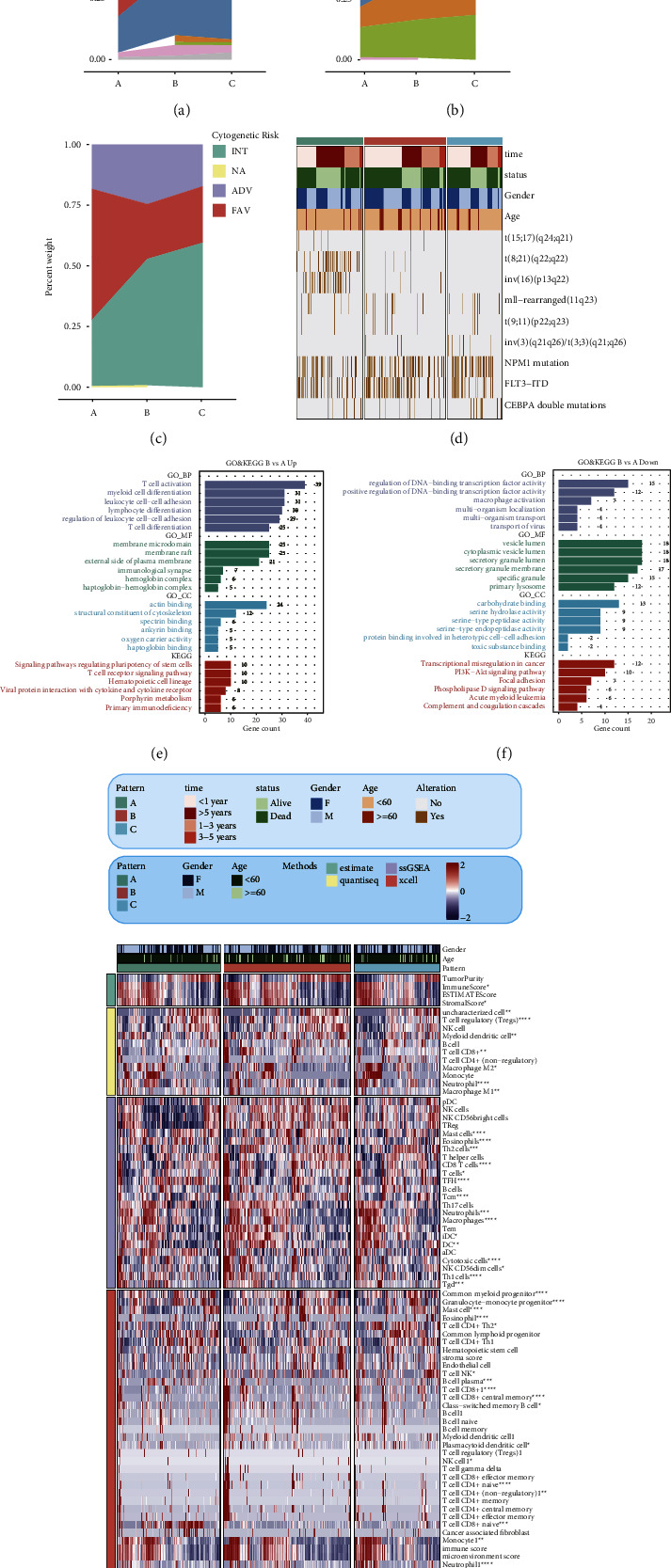
Description of the clinical traits, immune features, and biological function for the diverse patterns. The clinical features including French-American-British (FAB) classification (a), molecular features (b), cytogenetic risk (c), and the specific molecular features (d) were also investigated in the HOVON cohort in different patterns. GO and KEGG pathway enrichment analysis of the upregulated (e) and downregulated (f) differential expression genes between pattern B and pattern A, respectively. (g) Using four algorithms, including ESTIMATE, quanTIseq. XCell, and ssGSEA, the infiltration of immune cells was calculated in different patterns. The two-sided *p* value <0.05 was considered significant for all statistical analyses and shown as ^*∗*^*p* < 0.05, ^*∗∗*^*p* < 0.01, ^*∗∗∗*^*p* < 0.001, and ^*∗∗∗∗*^*p* < 0.0001.

**Figure 3:**
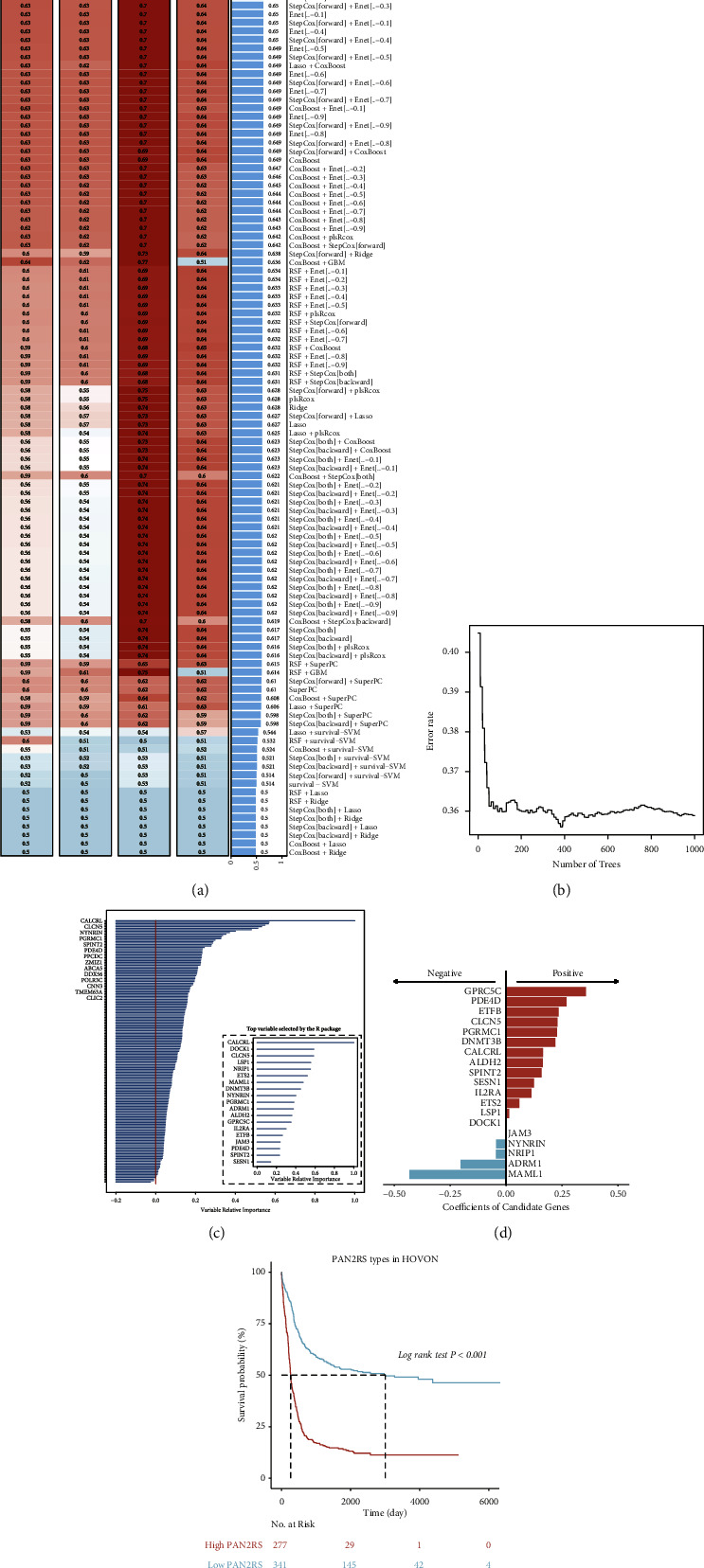
Development of the PANoptosis signature. (a) RSF was considered as the optimal algorithm with the highest mean C-index by the LOOCV framework. 19 genes were confirmed as signature genes by the RSF algorithm (b). Ranking these genes in order of relative importance, the top five genes are CALCRL, DOCK1, CLCN5, LSP1, and NRIP1 (c). (d) The coefficients of the 19 signature genes were calculated by multivariate Cox regression analysis. (e) The survival curves showing the significantly different overall survival between the high and low risk score subgroups.

Among the three patterns, the clinical features including French-American-British (FAB) classification, molecular features, and cytogenetic risk were also investigated in the HOVON cohort (Figures 2(a)–2(d)). The FAB classification system is a morphologic classification system for classifying acute leukemia into subtypes based on the type of cells that have developed and the degree of maturation of the cells. FAB in the worst prognosis pattern A was dominated by M2 and M4 subtypes, while FAB in the best prognosis pattern B is dominated by M5 subtype (Figure 3(a)). FLT3-ITD is a common driver mutation that manifests as a high leukemia burden in patients with acute myeloid leukemia with a poor prognosis. FLT3-ITD is closely associated with an unfavorable prognosis, leukocytosis, high white blood cell count, increased risk of relapse, and shortened overall survival. The FLT3-ITD-negative subtype was most common in pattern A and least common in pattern B (Figure 3(b)). Based on the treatment outcome, AML patients can be classified into three different cytogenetic risk groups, including favorable, intermediate, and unfavorable. The favorable subtype patients were mainly included in pattern A, while the unfavorable (ADV) subtype patients were mainly included in pattern B (Figure 3(c)). As previously described in the literature, some cytogenetic alterations such as (8; 21) (q22; q22), t (15; 17) (q24; q21), and inv (16) (p13; q22) are associated with longer palliation and life expectancy. Therefore, the specific molecular features were analyzed and the results showed that alterations of t (15; 17) (q24; q21), t (8; 21) (q22; q22), and inv (16) (p13; q22) occurred more frequently in pattern A, which had the best outcome among the patterns (Figure 3(d)). To explore the potential reasons contributing to the different prognosis among the PANoptosis-related patterns, pathway enrichment analysis and immune-related analysis including immune checkpoint gene expression, immune cell infiltration, and antitumor cycle were performed in the HOVON cohort. The 231 downregulated and 400 upregulated differently expressed genes were identified between pattern B and pattern A with |logFC| > 1 and *p* value <0.05. The results of the pathway enrichment analysis based on the identified genes, GO gene set and KEGG gene set, indicated that it was very likely that tumor immunity was the primary contributor to the deterioration of AML patients because those DEGs were enriched in the immune-related pathways, such as T cell activation, macrophage activation, T cell differentiation (GO BP), immunological synapse (GO MF), T cell receptor signaling pathway, primary immunodeficiency, and complement and coagulation cascades (KEGG) (Figures 2(e) and 2(f)). Therefore, we further investigated the differences in immunologic aspects between the different patterns. Using four algorithms, including ESTIMATE, quanTIseq XCell, and ssGSEA, the infiltration of immune cells was calculated, and the results showed that the infiltration of most immune cells differed among the three patterns, and it was particularly noteworthy that the distribution of T cells or macrophage-associated cells calculated by almost all methods differed among the three, with significantly higher expression in pattern B in particular (Figure 2(g)). The expression of immune checkpoint genes was also examined (Supplementary Figure 2A). Some common and classical immune checkpoint genes such as LAG3, CD27, and TIGIT were most and significantly expressed in pattern B. The antitumor immune cycle works by activating and enhancing the immune system's ability to recognize and destroy cancer cells. This cycle involves seven steps, including release of cancer cell antigens, antigen presentation, T cell priming and activation, T cell trafficking to the tumor, T cell infiltration into the tumor, T cell recognition of tumor cells, and T cell killing of tumor cells. We also analyzed the cycle steps among the different patterns and found that except for cancer cell antigen release and antigen presentation, the rest of the cycle steps were significantly different among the three patterns (Supplementary Figure 2B).

### 3.3. Development of the PANoptosis Signature

As mentioned in the Materials and Methods section, 5127 DEGs curated from the differential expression analysis between pattern B and pattern A were screened, and only 262 genes were retained by analysis of variance, univariate Cox regression analysis, and Kaplan–Meier survival analysis (Supplementary Table 8). RSF was considered as the optimal algorithm with the highest mean C-index by the LOOCV framework (Figure 3(a)). Among the 262 candidate genes, 19 genes were confirmed as signature genes by the RSF algorithm (Figure 3(b)). Ranking these genes in order of relative importance, the top five genes are CALCRL, DOCK1, CLCN5, LSP1, and NRIP1 (Figure 3(c)). The coefficients of the 19 signature genes were calculated by multivariate Cox regression analysis (Figure 3(d), Supplementary Table 9). The PAN2RS of each patient was calculated in all cohorts. The patients with high PAN2RS had a significantly worse prognosis than those with low PAN2RS (Figure 3(e)).

### 3.4. Evaluation and Validation of the Developed PANoptosis Signature

**Figure 4:**
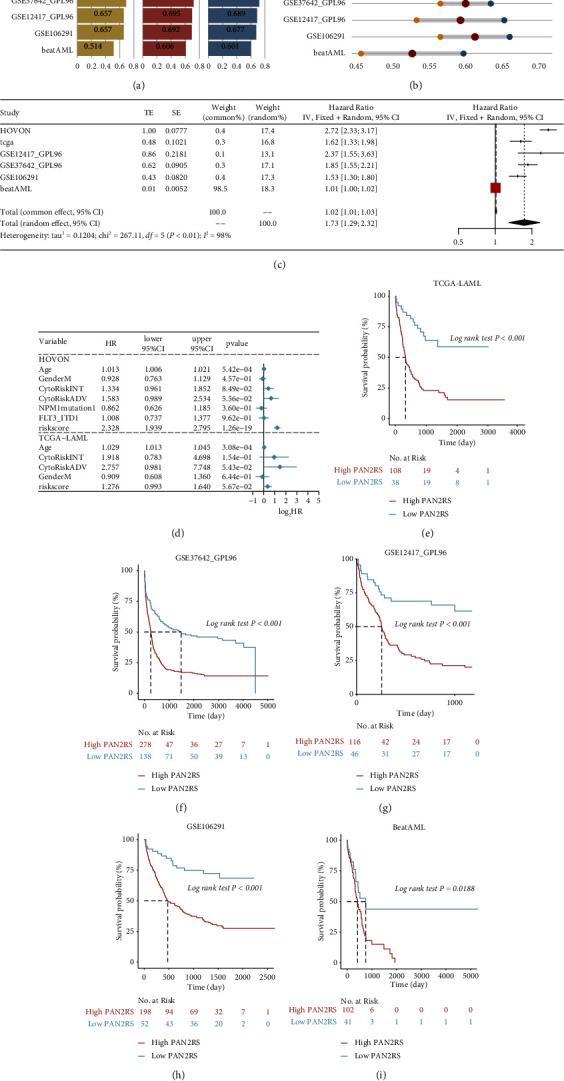
Evaluation and validation of the developed PANoptosis signature. (a) The 1-year, 3-year, and 5-year AUCs were calculated in six cohorts including TCGA-LAML, HOVON, GSE37642, GSE12417, GSE106291, and BeatAML. (b) The C-index of the developed signature was calculated in six cohorts. (c) The comprehensive HR of the signature was confirmed by the meta-analysis. (d) The multivariable Cox regression was carried out in the HOVON cohort and the TCGA-LAML cohort. (e–i) The KM curves showed that the patients with high risk score had a significant worse overall survival than those with low risk score, in TCGA-LAML, BeatAML, GSE37642, GSE12417, and GSE106291.

To assess the robustness of the signature, the 1-year, 3-year, and 5-year AUCs were calculated in six cohorts. The 1-year, 3-year, and 5-year AUCs were 0.7, 0.713, and 0.712 in the TCGA-LAML cohort, 0.747, 0.772, and 0.761 in the HOVON cohort, 0.689, 0.717, and 0.719 in GSE37642-GPL96, 0.657, 0.695, and 0.689 in GSE12417-GPL96, 0.657, 0.692, and 0.677 in GSE106291, and 0.514, 0.606, and 0.601 in BeatAML. All results indicated that the signature was robust and had a strong performance of outcome prediction (Figure 4(a)). The C-index was another index to evaluate the performance, and the result showed that the signature allowed a good prediction of the prognosis of AML patients, although the C-index in the BeatAML cohort was not satisfactory, which might be attributed to its relatively small cohort capacity (Figure 4(b)). The meta-analysis result based on the univariate Cox regression analysis result in each cohort also suggested that the PAN2RS had a stable, robust, and independent performance of prognosis prediction (Figure 4(c)). The multivariate Cox regression analysis regarding the PAN2RS and the clinical variables including age, gender, cytogenetic risk, NPM1 mutation, and FLT3-ITD mutation was adopted in HOVON cohort and TCGA-LAML cohort, suggesting the powerful and independent predictive ability (Figure 4(d)). In addition, and based on the optimal cutoff value, the patients in each cohort were divided into low and high PAN2RS subgroups (Supplementary Figures 3A and 3B). The Kaplan–Meier curves in the validation cohorts all indicated that patients with low PAN2RS had longer survival and better median survival (Figures 4(e)–4(i)).

### 3.5. Comparison of the PANoptosis Signature against the Published Signatures

**Figure 5:**
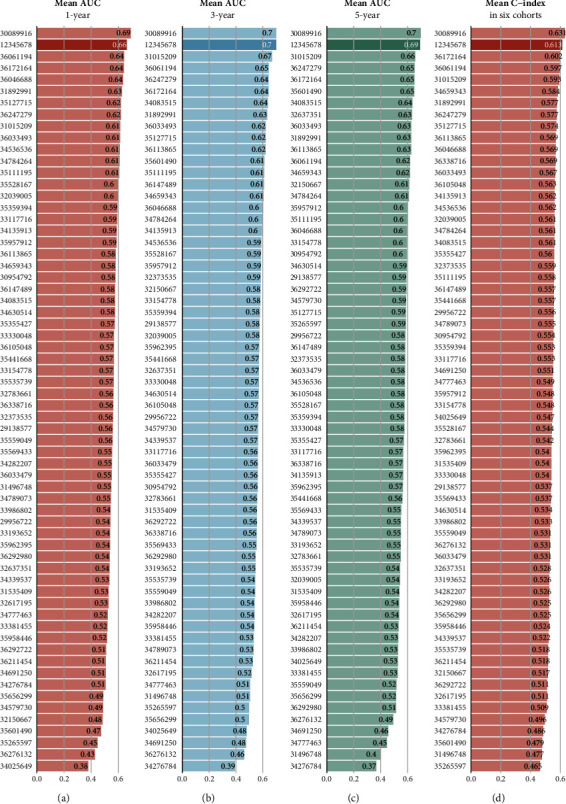
Comparison of the PANoptosis signature against the published signatures. Four metrics, including 1-year AUC (a), 3-year AUC (b), 5-year AUC (c), and C-index (d), were used to compare the developed PANoptosis signature with other published signatures. The left number indicated the PMID of each signature. 12345678 represented the signature developed in this study.

Four metrics, including 1-year AUC, 3-year AUC, 5-year AUC, and C-index, were used to compare the developed PANoptosis signature with other published signatures (Supplementary Figures 4–7). By comparing the average of each metric across all cohorts, we found that the developed signature (“12345678”) had a strong advantage, second only to the signature constructed by the article with PMID 30089916 in terms of performance in all aspects (Figures 5(a)–5(d)). In fact, from a certain point of view, the signature we constructed was even better. When we focused our attention on the performance of each indicator across all cohorts, it became clear that for the developed PANoptosis signature, the low value of the average of each indicator was due to the poor performance in the BeatAML cohort and the TCGA-LAML cohort. We excluded the indicator values of the signature when the signature genes did not match in the cohort. The signature with PMID 30089916 was only tested in the HOVON cohort and was inferior to the developed PANoptosis signature. This condition was observed in all four metrics (Supplementary Figures 4–7). These results indicated that the PANoptosis signature was a robust signature with a high level of predictive accuracy for the AML patients. Based on the collected signatures, multivariate Cox regression analysis was complemented in the HOVON cohort and the TCGA-LAML cohort, and the *p* value of PAN2RS of the signatures was compared (Supplementary Figures 8A and 8B).

### 3.6. Construction of a Clinically Useful Nomogram for AML Patients

**Figure 6:**
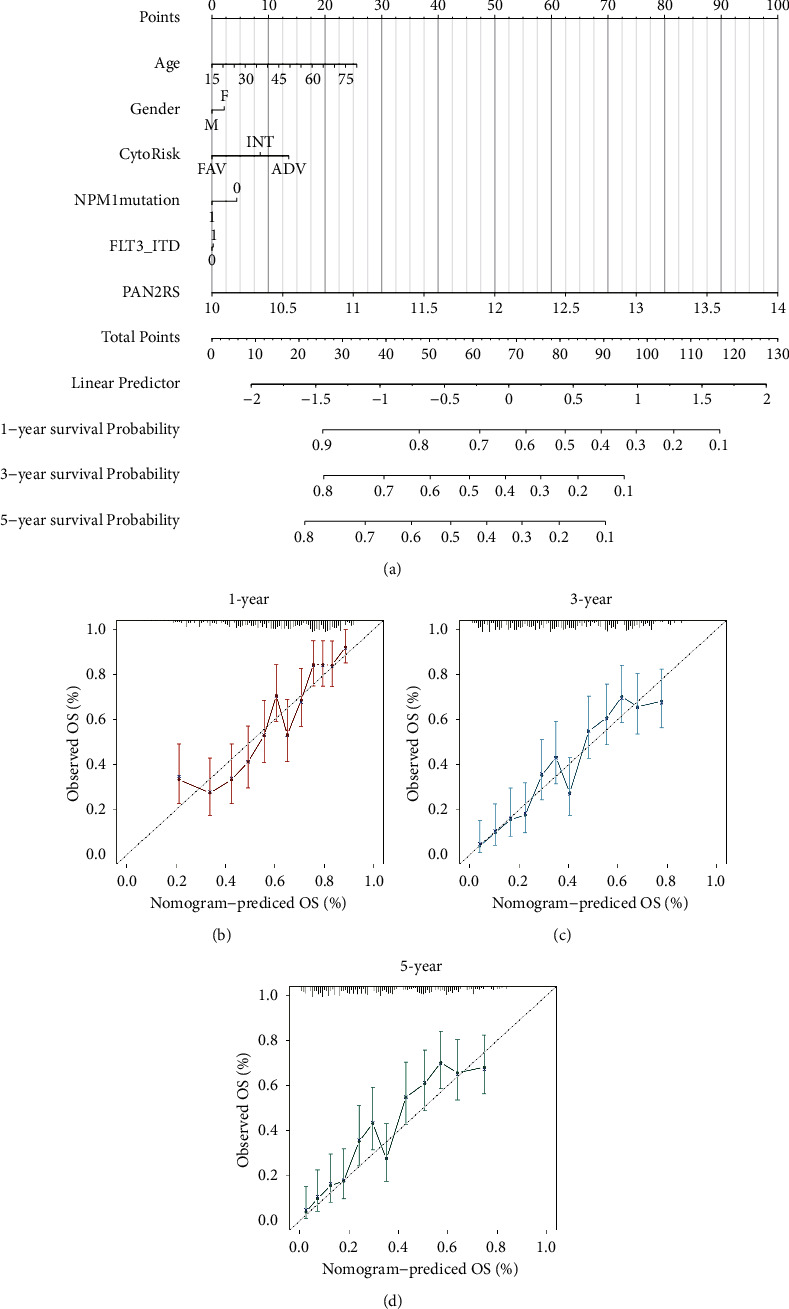
Construction of a clinically useful nomogram for AML patients. (a) A nomogram including PAN2RS and some clinical characteristics such as age, sex, cytogenetic risk, NPM1 mutation, and FLT-ITD mutation was constructed in the HOVON cohort. (b–d) The Platt calibration curves for 1-year, 3-year, and 5-year curves.

According to the multimethod analysis, a nomogram including PAN2RS and some clinical characteristics such as age, sex, cytogenetic risk, NPM1 mutation, and FLT-ITD mutation was constructed in the HOVON cohort to better guide clinical work and precision medicine (Figure 6(a)). There was almost no deviation from Platt calibration curves for 1-year, 3-year, and 5-year curves, indicating that the nomogram had excellent predictive accuracy (Figures 6(b)–6(d)).

### 3.7. Immune Profile and the Mutation Characteristics of Different PAN2RS Subgroups

**Figure 7:**
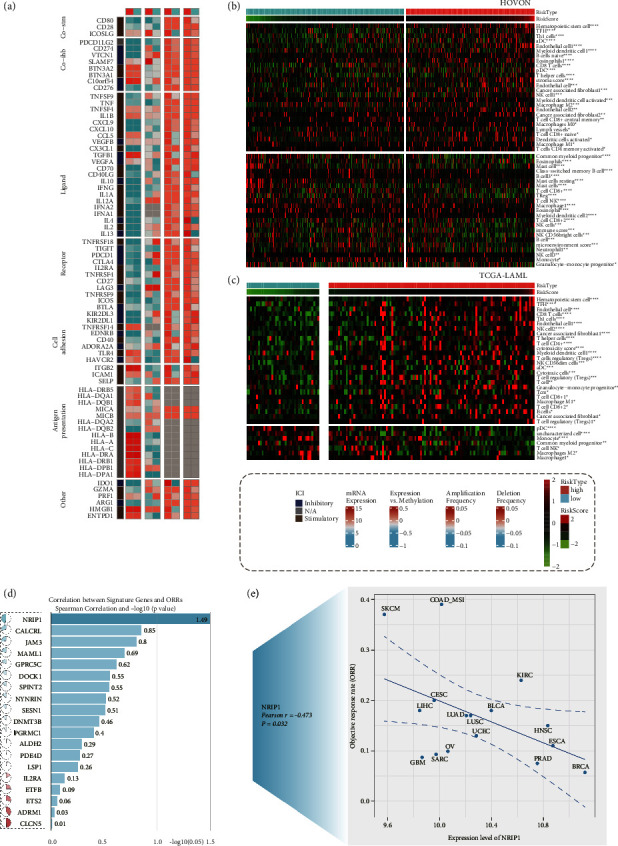
Immune profile and the mutation characteristic of the different PAN2RS subgroups. (a) The mRNA expression, correlation of expression and methylation, amplification frequency, and deletion frequency of immune checkpoint genes were comprehensively investigated in the high and low PAN2RS subgroups of the TCGA-LAML cohort. (b, c) The level of immune cell infiltration in the high and low PAN2RS groups and the correlation between the level of immune cell infiltration and PAN2RS were investigated in the HOVON cohort and the TCGA-LAML cohort. (d, e) The relationship between the 19 signature genes and the ORR of immunotherapy was explored, and the results indicated that NRIP1 could be a therapeutic target for adjuvant immunotherapy. ^∗^*p* < 0.05, ^∗∗^*p* < 0.01, ^∗∗∗^*p* < 0.001, and ^∗∗∗∗^*p* < 0.0001.

Influenced by the previously described significant differences in immune cells and immune cell-related pathways between the different PANoptosis patterns, we proposed here that the immune profiles should also be significantly different between the different PAN2RS groups. To test this hypothesis, we comprehensively investigated the mRNA expression, correlation of expression and methylation, amplification frequency, and deletion frequency of immune checkpoint genes in the high and low PAN2RS subgroups of the TCGA-LAML cohort (Figure 7(a)). Overall, there was no significant difference in the expression of these immune checkpoint genes at the mRNA level between the high and low PAN2RS groups, with only some genes such as CD28, IL12A, and IL2RA being more highly expressed in the high PAN2RS group. The methylation results showed opposite methylation status of most genes in the high-risk and low-risk groups, suggesting that these genes may be involved in the progression and prognosis of AML by altering the methylation status. The amplification frequency and deletion frequency of these genes were almost at a relatively high level in both the high PAN2RS group and the low PAN2RS group. A few genes, such as IDO1, IL4, and IL13, which showed higher amplification or deletion frequency in the high PAN2RS subgroup than in the low PAN2RS subgroup, might play a central role in the prognosis of AML patients. Seven algorithms were used to quantify the level of immune cell infiltration in all cohorts. We compared the level of immune cell infiltration in the high and low PAN2RS groups and also calculated the correlation between the level of immune cell infiltration and PAN2RS in all cohorts (Figures 7(b) and 7(c), Supplementary Figures 9–12). Based on the results of the analysis of different cohorts in multiple algorithms, we found that the PAN2RS was mainly positively associated with the infiltration of hematopoietic stem cells, CD8 T cells, endothelial cells, M2 macrophages, and regulatory T cells (Tregs) and mainly negatively associated with the infiltration of NK T cells and M1 macrophages. In addition, the relationship between the 19 signature genes and the ORR of immunotherapy was explored, and the results indicated that NRIP1 could be a therapeutic target for adjuvant immunotherapy, which may provide new opportunities for the treatment of AML patients with high NRIP1 activity (Figures 7(d) and 7(e), *p*=0.032, Supplementary Table 10). With the mutation data in TCGA-LAML cohort, the gene mutation characteristic in high PAN2RS group and low PAN2RS group was investigated, and the top 20 of the mutated genes were focused and displayed (Supplementary Figures 13A and 13B). The gene mutation rate of RUNX1 and TP53 was higher in the high PAN2RS subgroup. The top 5 of mutation genes were NPM1, RUNX1, TP53, ASXL1, and IDH2 in high PAN2RS subgroup, while the top 5 of mutation genes were KIT, NPM1, TTN, ARID1A, and CACNA1C in low PAN2RS subgroup. The somatic mutation of RUNX1 and TP53 had been proven to be associated with the worse outcome in AML, which was consistent with the results of our analysis. Previous studies had shown that TMB was low in AML, with less meaningful reference value to guide immunotherapy. Here, we investigated the correlation between PAN2RS and TMB in the TCGA-LAML cohort. PAN2RS was not significantly associated with TMB in AML (Supplementary Figure 13C, Spearman's sum rank correlation *r* = 0.04, *p*=0.6457), and there was no significant difference of TMB between the patients with high and low PAN2RS (Supplementary Figure 13D, Wilcoxon' test, *p*=0.94). These TMB-related analysis results were consistent with previous expectations.

### 3.8. Prediction of the Response to the Immunotherapy for AML Patients

**Figure 8:**
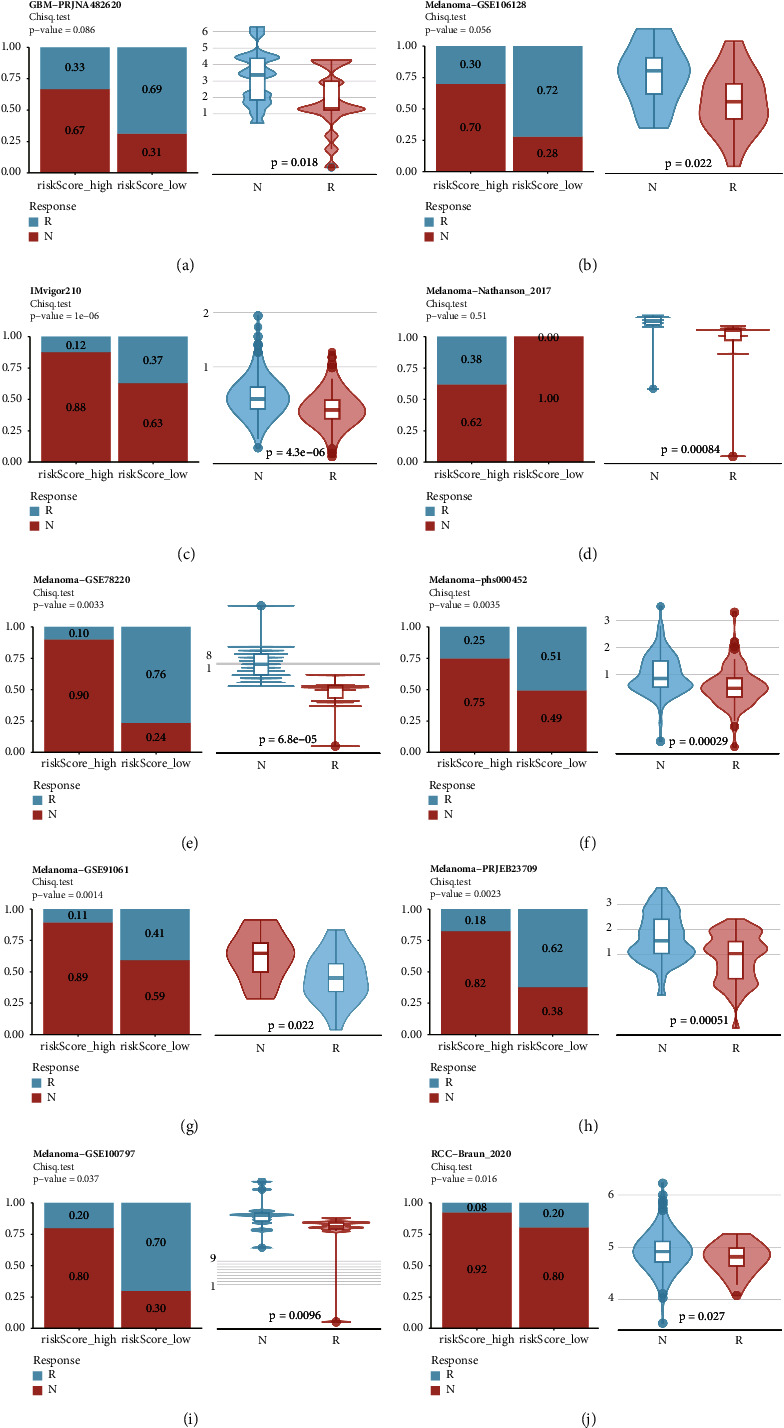
Prediction of the response to the immunotherapy for AML patients. (a–j) The association between PAN2RS and response to immunotherapy was investigated in ten cohorts that received immunotherapy.

AML is one of the diseases with immunologic treatment in the form of allogeneic stem cell transplantation. There are two primary treatment approaches in current practice: immunotherapy, including allogeneic hematopoietic stem cell transplantation, and the repositioning of effector cells (such as T cells, NK cells, and macrophages) to induce a rapid, potent, and long-lasting cytotoxic response, potentially resulting in immune memory [18, 19]. Despite their prolonged exposure to host immune cells, including cytotoxic T cells and natural killer cells, AML cells are able to evade immune attack, allowing them to inactivate cytotoxic lymphocytes both in direct and distant contact. Some mechanisms such as these make immunotherapy almost useless for some AML patients. Therefore, it is extremely important and economical to assess the efficacy of immunotherapy before administering it to AML patients. Here, we evaluated the association between PAN2RS and response to immunotherapy in ten cohorts that received immunotherapy. Based on PAN2RS, the patients were divided into high and low groups, and the patients with high PAN2RS all had worse survival time in all ten immunotherapy cohorts (Supplementary Figure 14). Moreover, Patients in the high PAN2RS group showed significantly lower levels of response to immunotherapy (significant in 7/10 cohorts) (Figure 8). Unsurprisingly, the patients responding to the immunotherapy showed significantly lower PAN2RS (Figure 8). Though it was a pity that there was no analyzed cohort of AML patients who had received immunotherapy, these results were still very informative. Overall, we proved that PAN2RS was highly associated with the response to the immunotherapy and high PAN2RS usually meant less response to the immunotherapy.

### 3.9. Prediction of the Therapeutic Targets and Agents for High PAN2RS AML Patients

**Figure 9:**
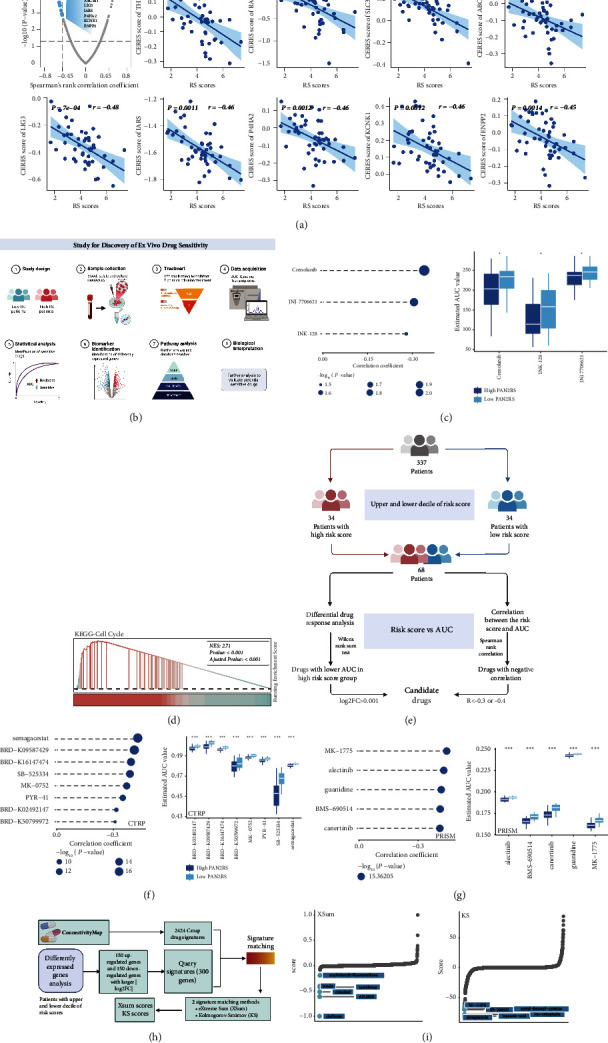
Prediction of the therapeutic targets and agents for high PAN2RS AML patients. (a) The correlation between CERES score and PAN2RS was investigated based on AML cell lines, and 9 potentially druggable targets for the poor survival AML patients were finally identified. (b) The workflow of a comprehensive ex vivo drug sensitivity analysis for AML patients with PAN2RS based on existing transcriptomic data and corresponding drug sensitivity data from previous literature. (c) Three drugs including crenolanib, JNJ-7706621, and INK-128 were identified as the potential therapeutic agents for the patients with high PAN2RS. All of them showed lower AUC values in the high PAN2RS group compared to the low PAN2RS group and were significantly negatively correlated with PAN2RS. (d) GSEA result of the KEGG cell cycle pathway. (e) The workflow of the ridge regression analysis for selecting the potential drugs. (f, g) Ridge regression analysis identified 8 and 5 drugs with high PAN2RS and poor prognosis using the CTRP and PRISM datasets, respectively. (h) The workflow of the drug selection based on the XSum score and KS score. (i) Based on the XSum score and KS score, clofibrate and alvespimycin were identified as most likely to be clinically effective in the treatment of AML patients with high PAN2RS. The two-sided *p* value <0.05 was considered significant for all statistical analyses and shown as ^*∗*^*p* < 0.05, ^*∗∗*^*p* < 0.01, ^*∗∗∗*^*p* < 0.001, and ^*∗∗∗∗*^*p* < 0.0001.

**Figure 10:**
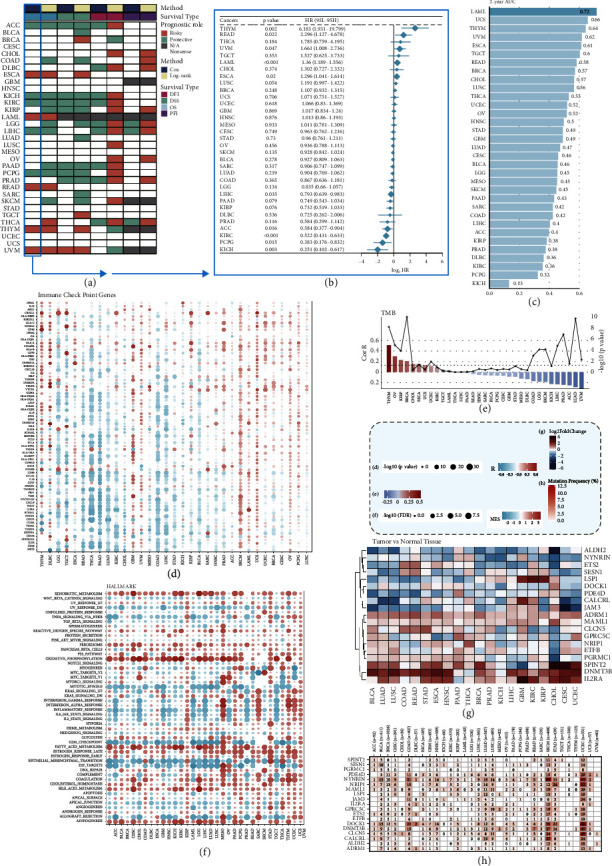
Extending the PANoptosis signature to pan-cancer. (a, b) The prediction accuracy of the PANoptosis signature was tested in other cancers, using the univariate Cox regression analysis and log-rank sum test. The PANoptosis signature had an excellent prediction performance in THYM, READ, UVM, LAML, ESCA, LIHC, ACC, KIRC, PCPG, and KICH. (c) The 2-year AUC of the PANoptosis signature was calculated across the cancer types. (d) The correlation of the PAN2RS and the expression of immune check point genes was investigated across the cancers. (e) The correlation of the PAN2RS and the TMB was investigated across the cancers. (f) The correlation of the PAN2RS and the hallmarks of the tumor was investigated across the cancers. (g) The heat map showing the expression of the signature genes across cancer types. (h) The heat map showing the somatic mutation of the signature genes, and NYNRIN, DOCK1, DNMT3B, NRIP1, CLCN5, and CALCRL had higher mutation frequency among the 19 genes.

To identify potential druggable targets for poor prognosis AML patients, information on 2249 targets for 4484 compounds was collected and analyzed to find candidate targets. We then investigated the correlation between CERES score and PAN2RS based on AML cell lines and finally identified 9 potentially druggable targets for the poor survival AML patients (Spearman's *r* < −0.45, *p* < 0.05) (Figure 9(a)). We found that the CERES values of some genes, including THBD, SLC36A2, P4HA2, KCNK1, and ENPP2, were greater than 0 in most AML cell lines, suggesting that these genes may not be very essential for the growth and proliferation of AML cells. The CERES scores of other genes, such as RAF1, ABCB11, LIG3, and IARS, were less than 0 in most AML cell lines, suggesting that AML patients with poorer prognosis may benefit from treatment targeting these genes. The CERES scores of the PANoptosis signature genes were also calculated. ADRM1, which had the lowest CERES score, might play an important role in the development and progression of AML (Supplementary Figure 15A). Given the significantly high association between high PAN2RS and poor patient survival, we further investigated whether patients with high PAN2RS scores could benefit from pharmacological treatment strategies. A comprehensive ex vivo drug sensitivity analysis was performed for AML patients with PAN2RS based on existing transcriptomic data and corresponding drug sensitivity data from previous literature (Figure 9(b)). To verify the reliability of this analysis, we had a number of drugs as positive controls. The drugs were sunitinib, foretinib, sorafenib, KW-2449, crenolanib, quizartinib, cabozantinib, dovitinib, NVP-TAE684, and Vargatef. These drugs had been shown in previous studies to be more effective than wild-type drugs in the treatment of AML patients with NPM1 mutations or FLT3-ITD mutations. And we compared the AUC of the drug between mutation type and wild type and found that the drugs as positive controls showed lower AUC value in NPM1 or FLT3-ITD mutation type than in wild type, indicating that the patients with NPM1 or FLT3-ITD mutation type were more sensitive to the drugs, which was consistent with the previous studies (Supplementary Figures 15B and 15C). And then a two-step analysis was carried out to find the high PAN2RS-dependent and sensitive agents (Figure 10(b)). Finally, three drugs including crenolanib, JNJ-7706621, and INK-128 were identified as the potential therapeutic agents for the patients with high PAN2RS. All of them showed lower AUC values in the high PAN2RS group compared to the low PAN2RS group and were significantly negatively correlated with PAN2RS (Figure 9(c)). Crenolanib, a receptor tyrosine kinase (RTK) inhibitor, may inhibit FLT3 kinase. JNJ-7706621, a novel and potent cell cycle inhibitor of CDK family members, may prevent tumor cell proliferation in multiple cancers. INK-128, a potent mTOR inhibitor, has shown encouraging potential in the treatment of cancer. We hypothesized that PAN2RS may represent underlying biological functions based on the fact that the three compounds associated with cell cycle and energy metabolism showed different AUC values in the high and low PAN2RS groups. To confirm this hypothesis, we first performed differential gene expression analysis in patients with the highest and lowest deciles of PAN2RS, followed by enrichment analysis based on four collected gene sets using two methods (GSEA and GSVA) to explore the underlying biological pathways. GSEA results based on KEGG, CO-BP, REACTOME, and HALLMARK gene sets showed high levels of biological signaling such as cell cycle-related pathways, glycolysis, and mTOR1 signaling in the high PAN2RS AML group, explaining the potential of these drugs to treat patients with high PAN2RS (Figure 9(d), Supplementary Figures 15D–15G). Similarly, with the GSVA algorithm, significant differences in cell division and energy metabolism pathways were found between the high and low PAN2RS groups (Supplementary Figure 16A). Ridge regression analysis then identified 8 and 5 drugs with high PAN2RS and poor prognosis using the CTRP and PRISM datasets, respectively (Figures 9(e)–9(g), Supplementary Figure 16B). All of these drugs were significantly negatively associated with PAN2RS and showed significantly lower AUC values in the high PAN2RS group (Figures 10(e)–10(g)). The XSum score and the KS score were calculated to search for the potential drugs that could reverse the process of AML (Figure 9(h)). Based on the XSum score and KS score, clofibrate and alvespimycin were identified as most likely to be clinically effective in the treatment of AML patients with high PAN2RS (Figure 9(i)).

### 3.10. Extending the PANoptosis Signature to Pan-Cancer

**Figure 11:**
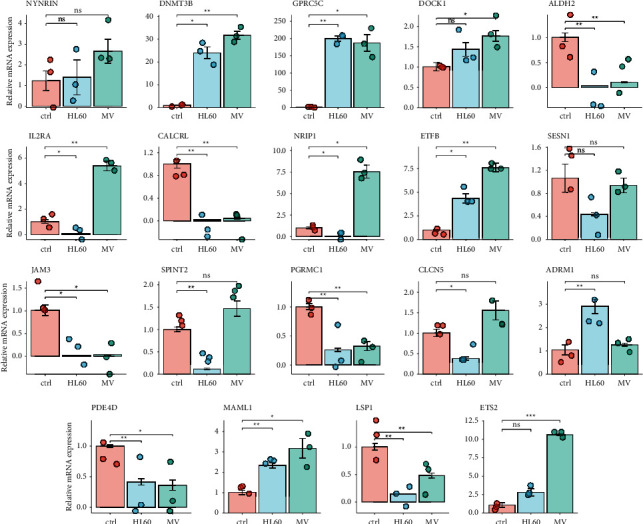
Validation of 19-gene signature expression in acute myeloid leukemia. qRT-PCR was performed to validate the mRNA expression levels of 19 genes in our signature with MV-4-11 cell line and HL-60 cell line. Ctrl means control, HL60 means HL-60, and MV means MV-4-11. ^∗^*p* < 0.05, ^∗∗^*p* < 0.01, ^∗∗∗^*p* < 0.001, and ^∗∗∗∗^*p* < 0.0001.

The correlation of the PANoptosis signature and the tumor-related pathway signaling had been comprehensively analyzed above. We speculated that this association would also exist in other tumors. In order to verify this, we first tested the prediction accuracy of the PANoptosis signature in other cancers, using the univariate Cox regression analysis and survival analysis. The PANoptosis signature had an excellent prediction performance in THYM, READ, UVM, LAML, ESCA, LIHC, ACC, KIRC, PCPG, and KICH (Figures 10(a) and 10(b)). 1-year, 2-year, 3-year, 4-year, and 5-year AUCs of the PANoptosis signature were calculated across the cancer types, and the results indicated that the PANoptosis signature had a high prediction accuracy in some cancers such as THYM, TGCT, UVM, and READ (Figure 10(c), Supplementary Figures 17A–17E). Moreover, the correlation of the PAN2RS and the expression of immune check point genes was investigated across the cancers, suggesting that the association in SKCM, OV, UCS, GBM, and UVM was similar to the correlation in LAML, while that in READ, THCA, PAAD, LUAD, and COAD was opposite to that in LAML (Figure 10(d)). This revealed different patterns of expression of immune checkpoint genes and signature genes in different cancers. From the previous analysis, the TMB measurement in AML was found to be of low predictive value; however, the study of TMB in other tumors with high levels of TMB is important as a measure of the level of response to immunotherapy. PAN2RS was significantly and positively associated with TMB in THYM, OV, KIRP, BRCA, and UCEC, whereas UVM, LUAD, ACC, PRAD, LIHC, SKCM, LGG, and COAD showed the opposite (Figure 10(e)). And then, the correlation of the PAN2RS and the hallmarks of the tumor was investigated. All the hallmark gene sets were at a high level in the high PAN2RS patients of LAML, UCS, and UVM. Metabolism-related pathways including xenobiotic metabolism, fatty acid metabolism, and oxidative phosphorylation were present at high levels in patients with high PAN2RS across the cancer types. The levels of these pathways were largely consistent and high in PAN2RS AML patients; however, this consistency was rarely seen in solid tumors, and some pathways were even downregulated in the high PAN2RS groups, which could be due to the great heterogeneity between solid tumors and hematologic tumors and required further investigation (Figure 10(f)). In addition, the expression of the signature genes was examined across cancer types. The results showed that IL2RA, DNMT3B, ADRM1, and NRIP1 were highly expressed in tumor tissue compared to normal tissue, while ALDH2, NYNRIN, LSP1, and CALCRL were the opposite (Figure 10(g)). The somatic mutation of the signature genes was also explored, and NYNRIN, DOCK1, DNMT3B, NRIP1, CLCN5, and CALCRL had higher mutation frequency among the 19 genes and the main type of mutation was missense mutation (Figure 11, Supplementary Figure 18). Finally, we examined the correlation of the PAN2RS and the immune cell infiltration quantified by five algorithms including CIBERSORT, EPIC, ESTIMATE, quanTIseq, and xCell. PAN2RS was significantly and positively associated with the infiltration of CD4 T cells, Tregs, and M2 macrophages while negatively associated with the infiltration of NK cells, memory B cells, and Th1 cells across cancer types (Supplementary Figure 19A–19E), consistent with the previous conclusion.

### 3.11. Validation of 19-Gene Signature Expression in Acute Myeloid Leukemia

We compared the mRNA expression of the candidate genes with the combination analysis of the TCGA-LAML and the GTEx data (Supplementary Figure 20). The qRT-PCR was performed to validate the mRNA expression levels of 19 genes in our signature. As shown in Figure 11, upregulated NYNRIN, DNMT3B, GPRC5C, DOCK1, ETFB, ADRM1, MAML1, and ETS2 were found in both HL-60 and MV-4-11 cells while ALDH1, CALCRL, JAM3, PGRMC1, PDE4D, LSP1, and SESN1 were downregulated in AML. The genes NRIP1, IL2RA, SPINT2, and CLCN5 were upregulated in MV-4-11 cell and downregulated in HL-60 cell.

## 4. Discussion

PANoptosis is a recently discovered type of programmed cell death that promotes tumor cell death and perhaps reduces the effect of aberrant apoptotic pathways on chemoresistance in tumors [20]. However, few studies on the relationship between PANoptosis and AML exist. Relapse is one of the leading causes of AML-related deaths. Abundant evidence suggests that the abnormal expression of gene affects the poor prognosis AML cells [21–26]. How gene expression influences PANoptosis is yet unknown. In this study, we established a novel PANoptosis-related prognostic gene (PANRGs) signature for AML patients.

We comprehensively investigated the PANoptosis patterns in AML and identified some key PANoptosis-related candidate genes, and with the LOOCV framework, we developed a prognostic signature consisting of 19 genes and corresponding coefficient.

By comparing the average of each metric across all cohorts, we found that the developed signature (“12345678”) had a strong advantage, second only to the signature constructed by the article with PMID 30089916 in terms of performance in all aspects. In fact, from a certain point of view, the signature we constructed was even better. When we focused our attention on the performance of each indicator across all cohorts, it became clear that for the developed PANoptosis signature, the low value of the average of each indicator was due to the poor performance in the BeatAML cohort and the TCGA-LAML cohort. We excluded the indicator values of the signature when the signature genes did not match in the cohort. The signature with PMID 30089916 was only tested in the HOVON cohort and was inferior to the developed PANoptosis signature. This condition was observed in all four metrics. These results indicated that the PANoptosis signature was a robust signature with a high level of predictive accuracy for the AML patients compared with the other prognostic signatures of AML patients.

We also compared enrichment score of infiltration of immune cells and immune pathways between the high- and low-risk groups, investigated functional mechanisms via GSEA, and assessed potentially suitable drugs. This novel 19-gene signature may contribute to the improvement in the prediction of AML prognosis and patient stratification for therapeutic strategies.

The PGERs NYNRIN, DNMT3B, GPRC5C, DOCK1, ETFB, ADRM1, MAML1, ETS2, ALDH2, CALCRL, JAM3, PGRMC1, PDE4D, LSP1, and SESN1 were included in our signature. The genes in our signature were related to tumor predisposition (NYNRIN [27], GPRC5C [24], and CLCN5 [28]), tumorigenesis (JAM3 [29], PGRMC1 [30], and LSP1 [31]), progression (DOCK1 [32], MAML1 [33], PGRMC1, NRIP1, and PDE4D [34]), migration and invasion (ADRM1 [35] and SPINT2 [36]), and chemotherapy sensitivities (ALDH2 [25], ETFB [37], ETS2 [38], CALCRL [23], SESN1 [39], and IL2RA [40]). In detail, NYNRIN gene was related to Wilms tumor predisposition [27]. Wong et al. found that DNMT3B contributes to the progression and severity of AML [24]. GPRC5C is consistently elevated exclusively in neuroblastoma cancer stem cells [32]. DOCK1, as a member of DOCK family, can encode evolutionarily conserved guanine nucleotide exchange factors for Rho GTPase to enhance the progression of AML [41]. Exome array analysis identifies ETFB as a novel susceptibility gene for anthracycline-induced cardiotoxicity in cancer patients [37]. ADRM1 gene amplification is a candidate driver for metastatic gastric cancers [35]. MAML1/2 promotes YAP/TAZ nuclear localization and tumorigenesis [33]. ETS2 acts by regulating expression of hematopoietic lineage and transcription factor genes involved in erythropoiesis and megakaryopoiesis and in chemotherapy sensitivities [38]. Inhibition of ALDH2 can sensitize AML cells to chemotherapy [25], which means ALDH2 may be a target to chemoresistance. CALCRL-associated genes could also potentially mediate the chemoresistance and relapse of AML [23]. JAM3 functions as a novel tumor suppressor and is inactivated by DNA methylation in colorectal cancer [29], and the role in leukemia is unknown. PGRMC1 is an enigmatic heme-binding protein, is highly expressed in breast cancer tissue, and may be important in tumorigenesis [42]. PDE4D binds and interacts with YAP to cooperatively promote HCC progression [34]. The distinct morphological characteristics of hairy cell leukemia (HCL) cells can be attributed to the overexpression of pp52 (LSP1) and/or its specific association with the cytoskeleton. Elevated cytoskeleton-binding pp52 (LSP1) protein contributes to the distinctive morphology of hairy cell leukemia [31]. Suppression of SESN1 reduces cisplatin and hyperthermia resistance through increasing reactive oxygen species (ROS) in human maxillary cancer cells [39]. The top five genes related to AML are DOCK1, ALDH2, LSP1, NRIP1, and IL2RA. Focus on chemotherapy sensitivity and resistance may be the potential target to AML treatment.

In our research, we also conducted a comprehensive drug sensitivity analysis to identify potential therapeutic options for AML patients with high PAN2RS expression. Our results indicated that clofibrate, a fibric acid derivative primarily used in the treatment of hypertriglyceridemia and dyslipidemia, may exhibit clinical benefits by modulating superoxide anion production, lipoperoxidation, and reactive oxygen species production [43]. Additionally, alvespimycin, a heat shock protein 90 inhibitor, demonstrated promising antileukemia activity in advanced AML patients [44]. Notably, alvespimycin has also shown efficacy in overcoming imatinib resistance in chronic myeloid leukemia cell lines [45]. These findings suggest that both clofibrate and alvespimycin could serve as potential therapeutic interventions in the management of AML.

PANoptosis was first known in normal cells or tissues under various physiological or pathological conditions [46]. Several risk signatures of PANoptosis have been established, which mainly focus on solid tumors. Our research is the first signature of PANoptosis related to AML. Although we have integrated this predictive model into the R package to enhance its clinical utility, there are many limitations in our research. First, only retrospective data were used to develop and validate the signature. More prospective clinical studies should be collected to validate the robustness and effectiveness of the constructed signature. Second, a single signature was used to construct a prognostic model, which may lead to the loss of many key prognostic genes in AML. Third, the detailed roles of PANRGs in AML, including in vivo and in vitro, should be further investigated in the future. Fourth, although we collected a large amount of transcriptomic data, larger samples with multiomic data should be collected to investigate the role of PANoptosis in AML.

## 5. Conclusion

Collectively, by integrative analysis of sequencing data of LAML cohorts, pan-cancer cohorts, and human cancer cell lines based on a wealth of machine learning algorithms, our study established a novel, stable, and robust 19-PANRG prognostic risk signature for AML patients. It is a promising tool for personalizing treatment and clinical management for individual AML patients. In addition, FRGs may represent novel therapeutic targets in AML.

## Data Availability

All data associated with this study are available in the main text or the supplementary materials. We have integrated this PAN2RS into the R package PANScore (https://github.com/zwxiangya/PANScore). Further inquiries can be directed to the corresponding author.

## Supplementary Materials

Supplementary Materials Supplementary Tables provide a helpful elucidation of the researched genes and their background, as well as the sources and comparison of the data. Supplementary Figures 1 to 20 provide a more detailed description of the origin of this model and the advantages and disadvantages compared to other models. They can help elucidate the purpose of this article.
