# Supplementary material for: Machine Learning-Based Integrated Analysis of PANoptosis Patterns in Acute Myeloid Leukemia Reveals a Signature Predicting Survival and Immunotherapy
Source: Int J Clin Pract. 2024 Jan 30;2024:5113990. doi: 10.1155/2024/5113990 (PMC10846924; doi:10.1155/2024/5113990)
Supplement: Supplementary Materials — Supplementary Tables provide a helpful elucidation of the researched genes and their background, as well as the sources and comparison of the data. Supplementary Figures 1 to 20 provide a more detailed description of the origin of this model and the advantages and disadvantages compared to other models. They can help elucidate the purpose of this article. [file 5113990.f1.zip › Supplementary figure.20231227.R.pdf]

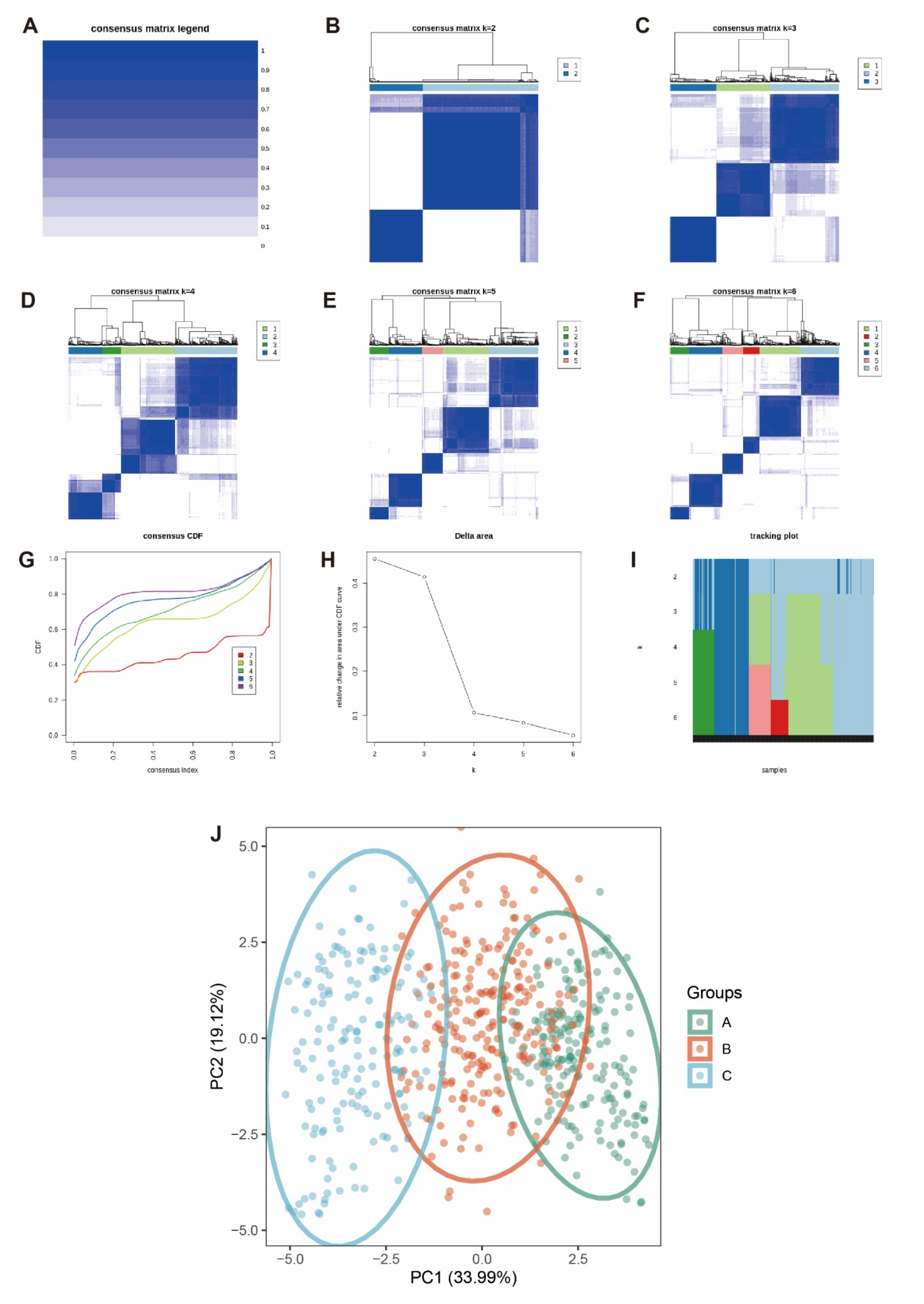

Supplementary figure 1. A-I. The result of the consensus clustering analysis. J. The PCA plot shown the different

distribution of the PANoptosis groups.

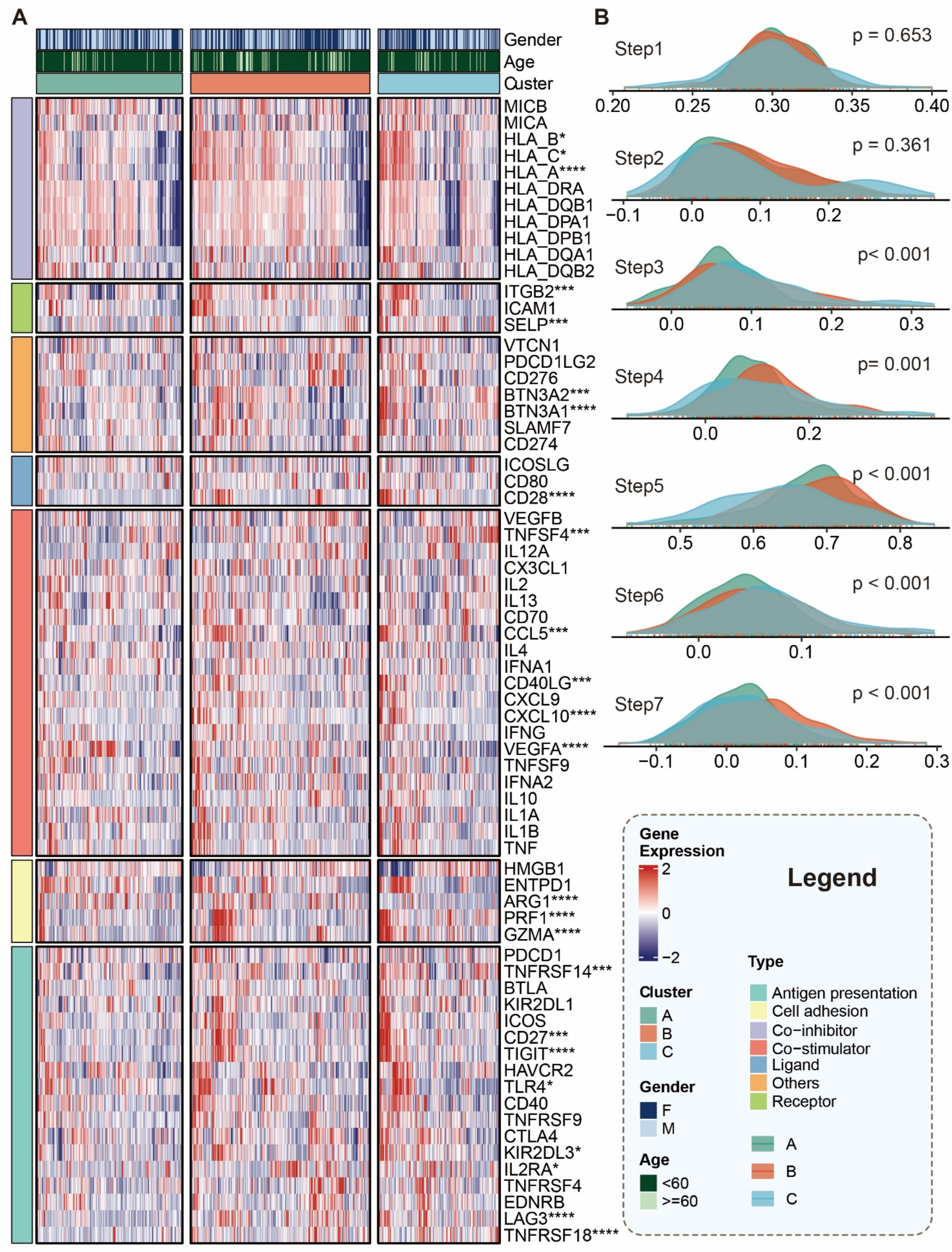

Supplementary figure 2. A. The expression of immune checkpoint genes was also examined in different PANoptosis groups. B. The steps of the anti-tumor immune cycle were calculated in the three subgroups. The two- sided p value < 0.05 was considered of significance for all statistical analyses and shown as * $p < 0.05$, ** $p<0.01$, *** $p < 0.001$ and **** $p < 0.0001$.

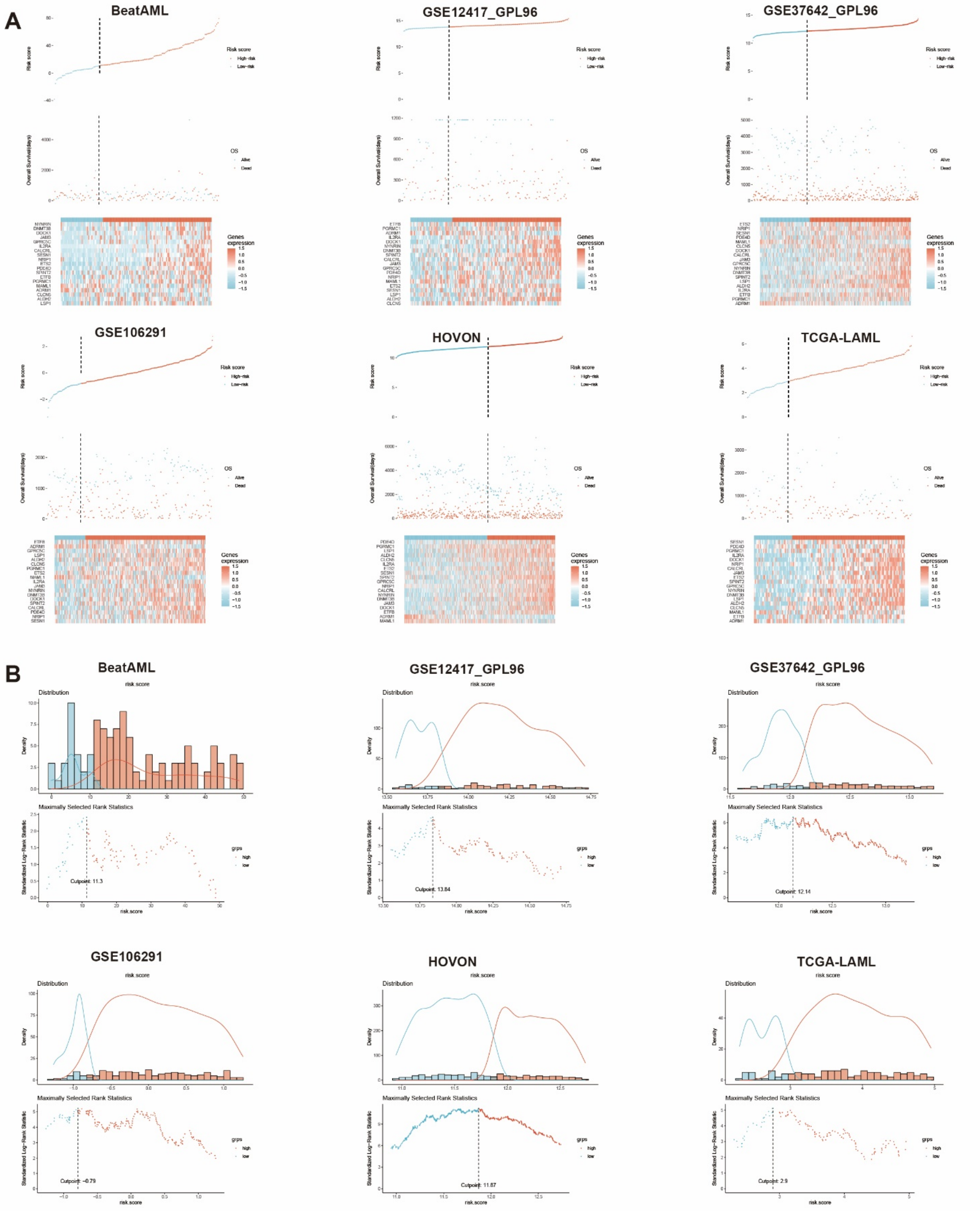

Supplementary Figure 3. A-B. Based on the optimal cut-off value, the patients in each cohort were divided into low

and high PAN2RS subgroups.

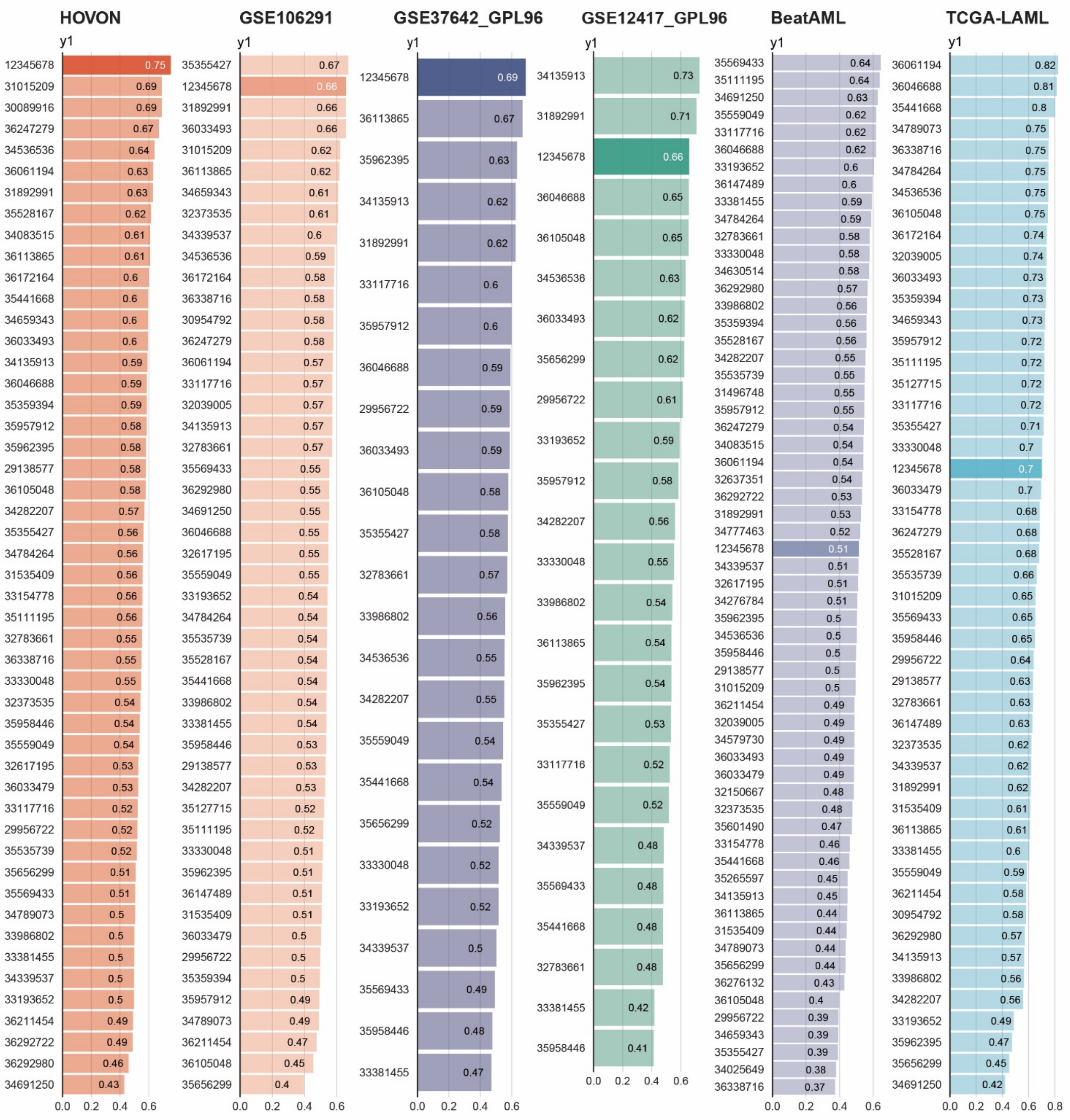

Supplementary Figure 4. The 1-year AUC was used to compare the developed PANoptosis signature with other published signatures. The left number indicated the PMID of each signature. The 12345678 represented the signature developed in this study.

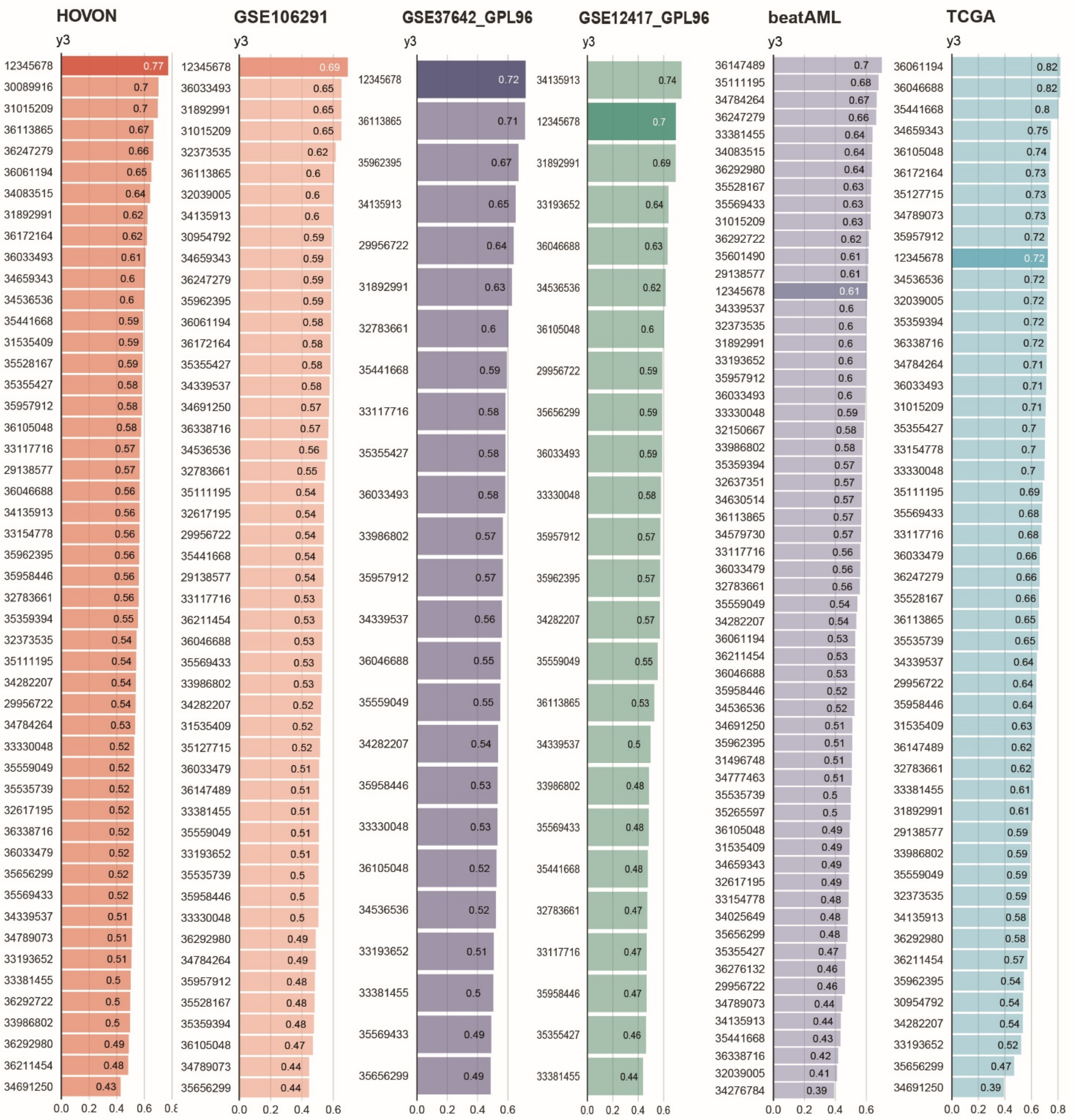

Supplementary Figure 5. The 3-year AUC was used to compare the developed PANoptosis signature with other published signatures. The left number indicated the PMID of each signature. The 12345678 represented the

signature developed in this study.

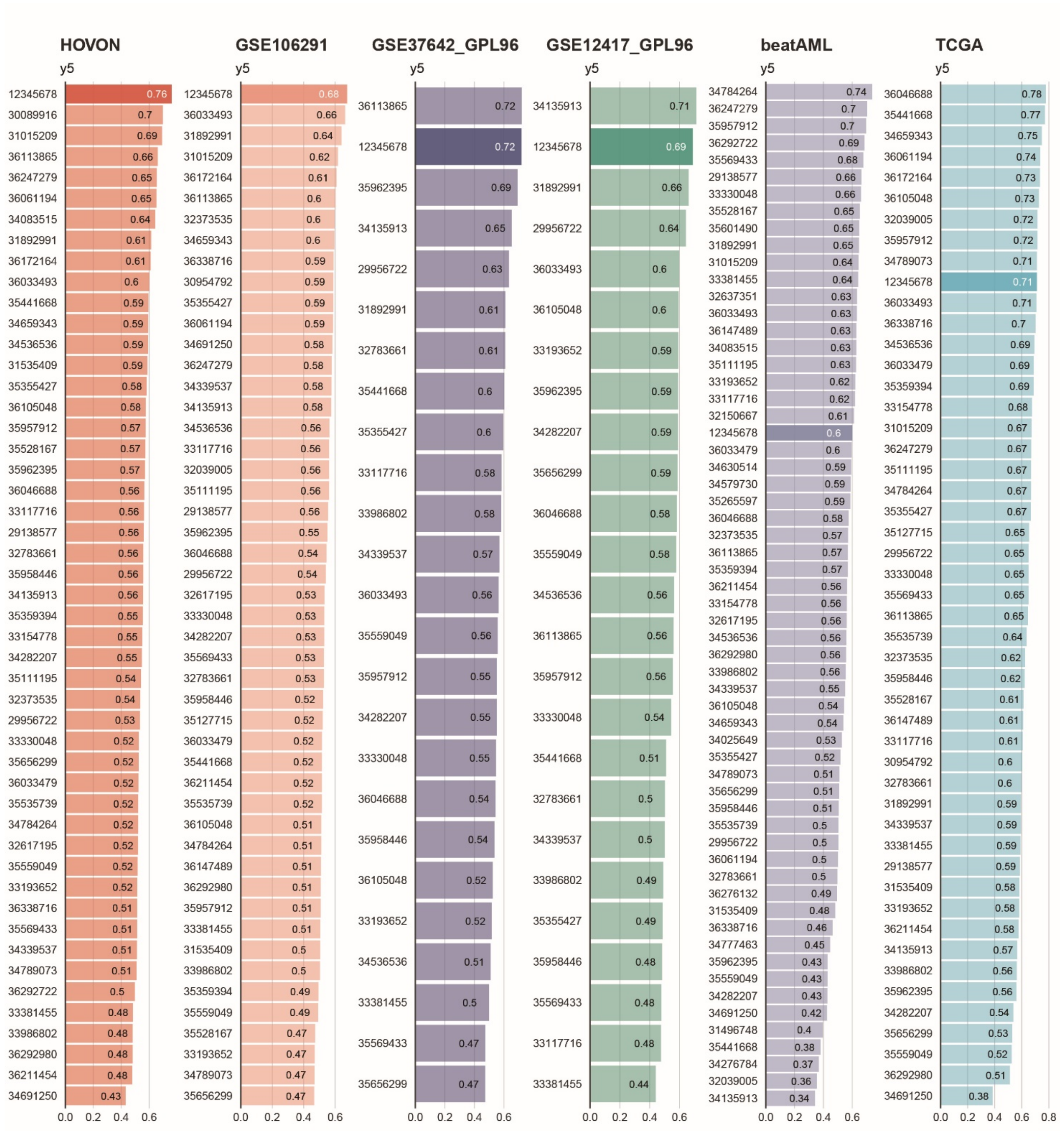

Supplementary Figure 6. The 5-year AUC was used to compare the developed PANoptosis signature with other published signatures. The left number indicated the PMID of each signature. The 12345678 represented the

signature developed in this study.

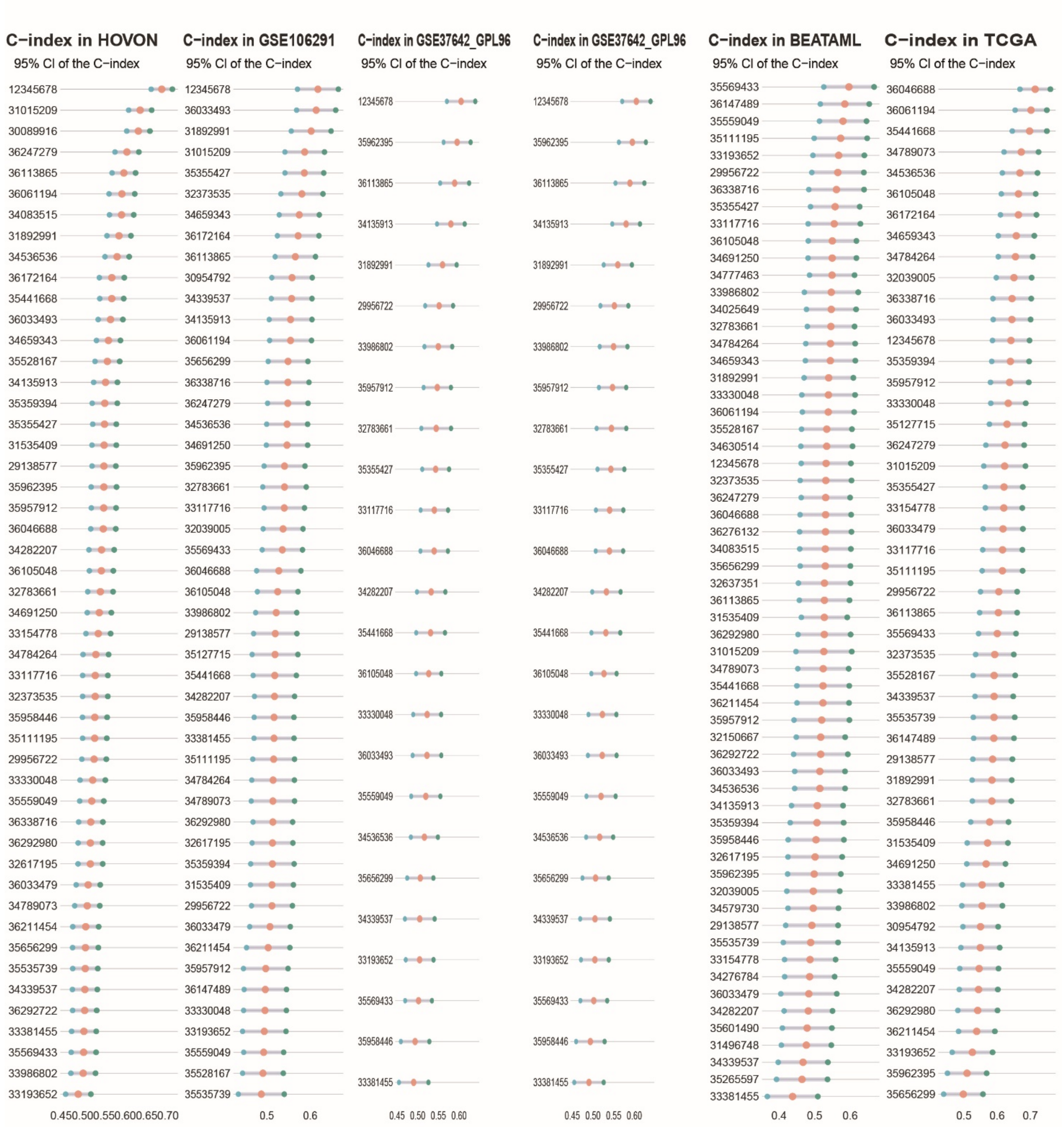

Supplementary Figure 7. The C-index was used to compare the developed PANoptosis signature with other published signatures. The left number indicated the PMID of each signature. The 12345678 represented the

signature developed in this study.

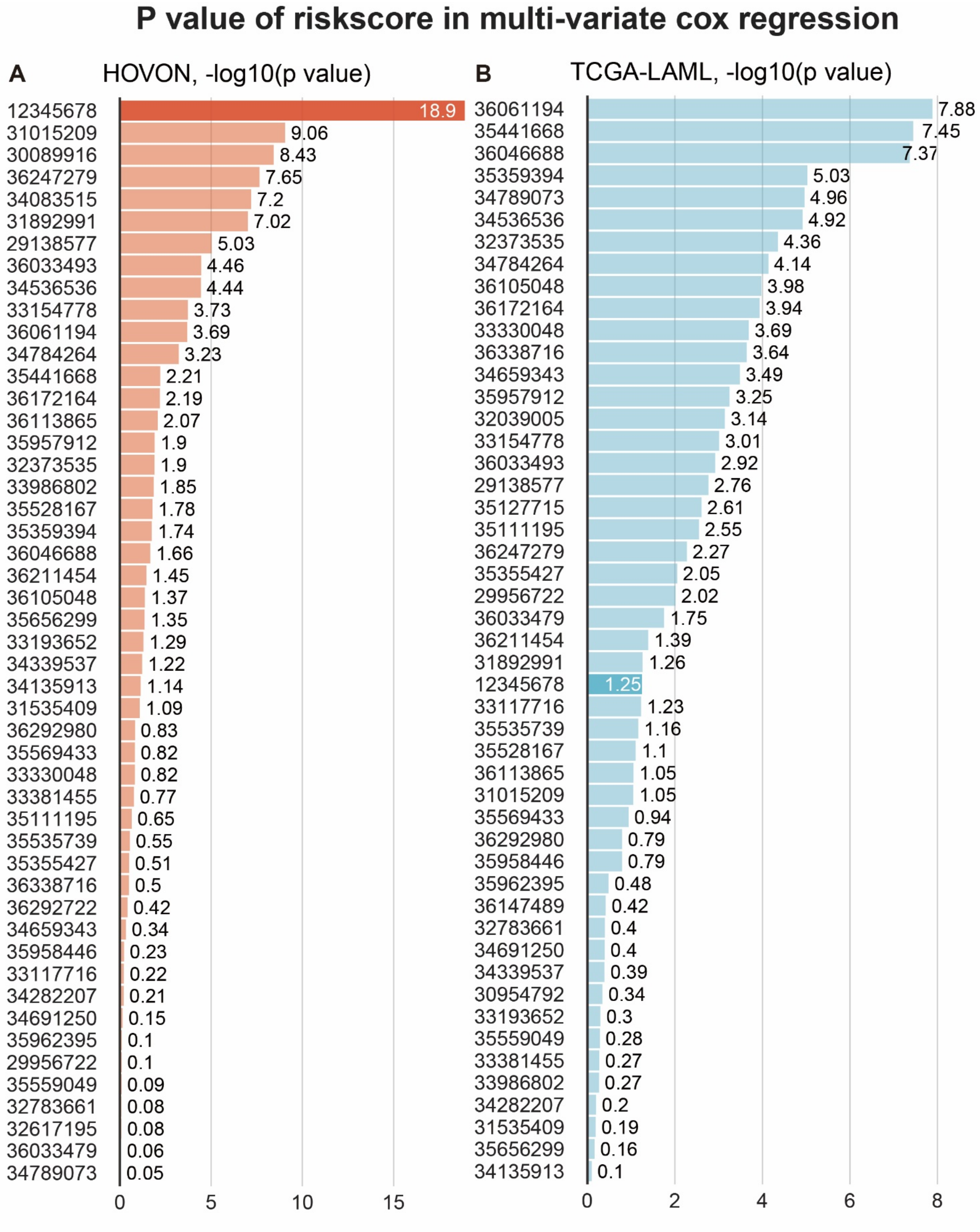

Supplementary Figure 8. Based on the collected signatures, multivariate Cox regression analysis was complemented in the HOVON cohort (A) and the TCGA-LAML cohort (B), and the p-value of PAN2RS of the signatures was compared.

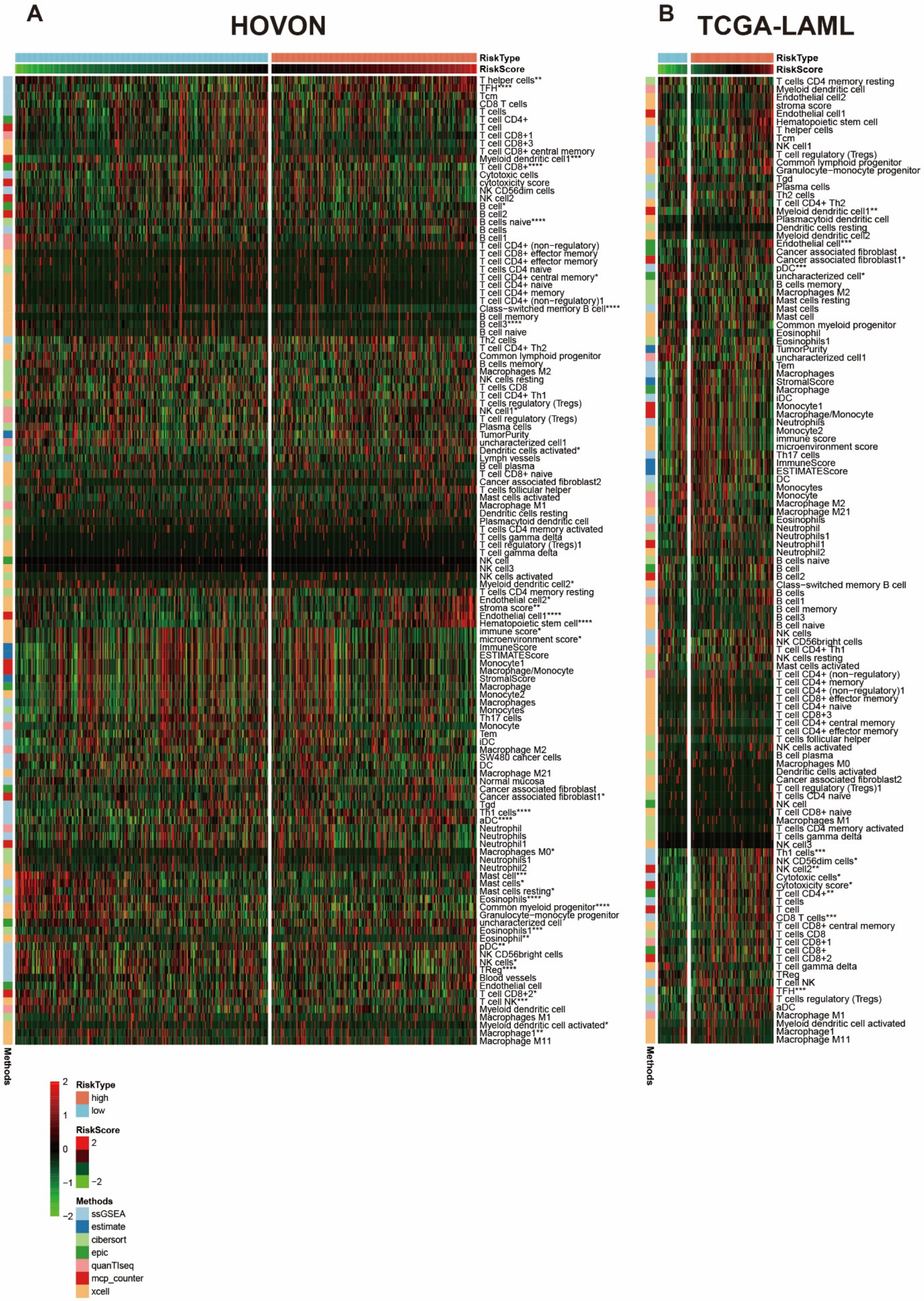

Supplementary Figure 9. The level of immune cell infiltration in the high and low PAN2RS groups were

investigated with Wilcoxon sum test in HOVON and TCGA-LAML cohorts. The two- sided p value < 0.05 was considered of significance for all statistical analyses and shown as * $p < 0.05$, ** $p<0.01$, *** $p < 0.001$ and **** $p < 0.0001$.

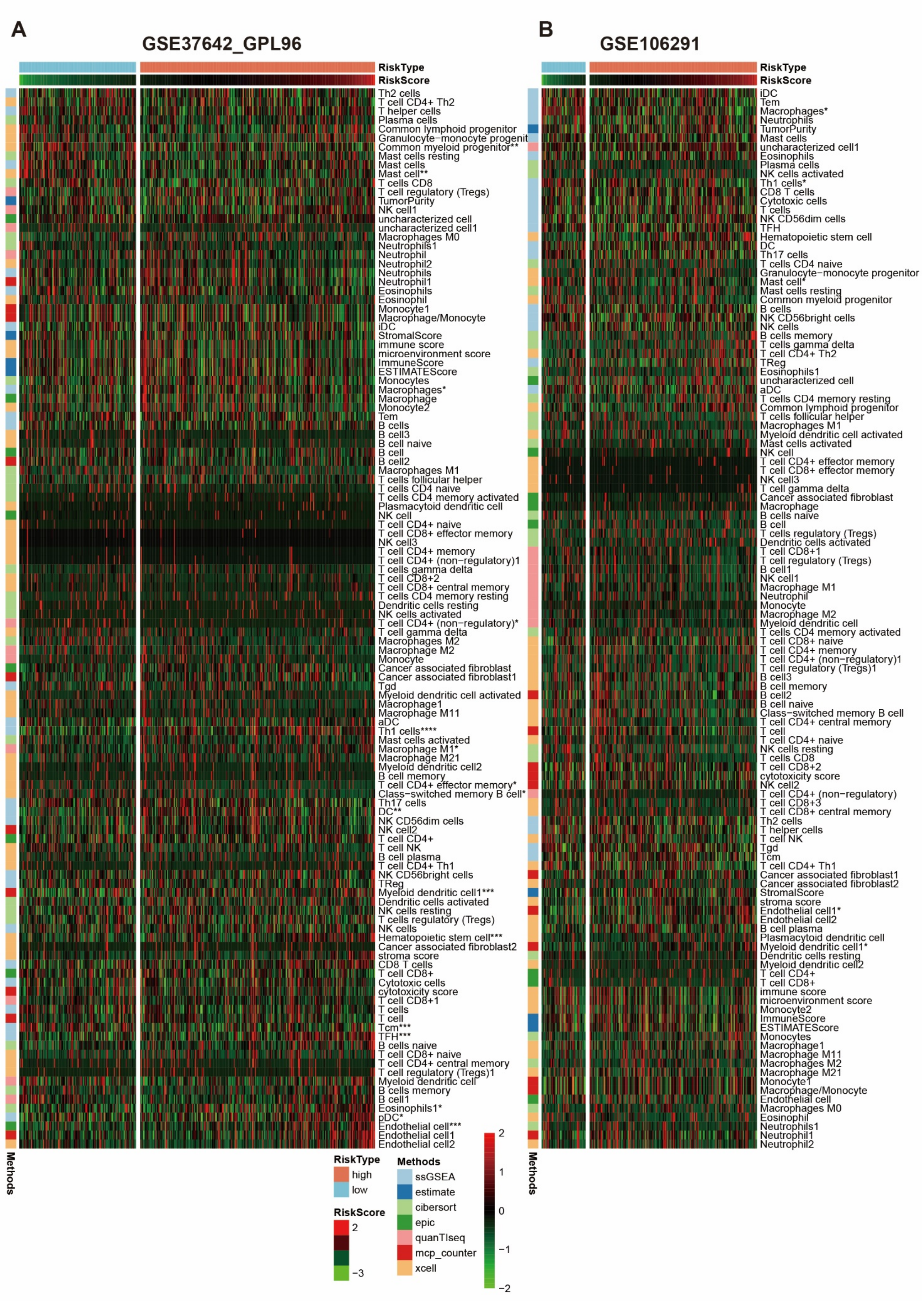

Supplementary Figure 10. The level of immune cell infiltration in the high and low PAN2RS groups were

investigated with Wilcoxon sum test in GSE37642 and GSE106291 cohorts. The two- sided p value < 0.05 was considered of significance for all statistical analyses and shown as * $p < 0.05$, ** $p<0.01$, *** $p < 0.001$ and **** $p < 0.0001$.

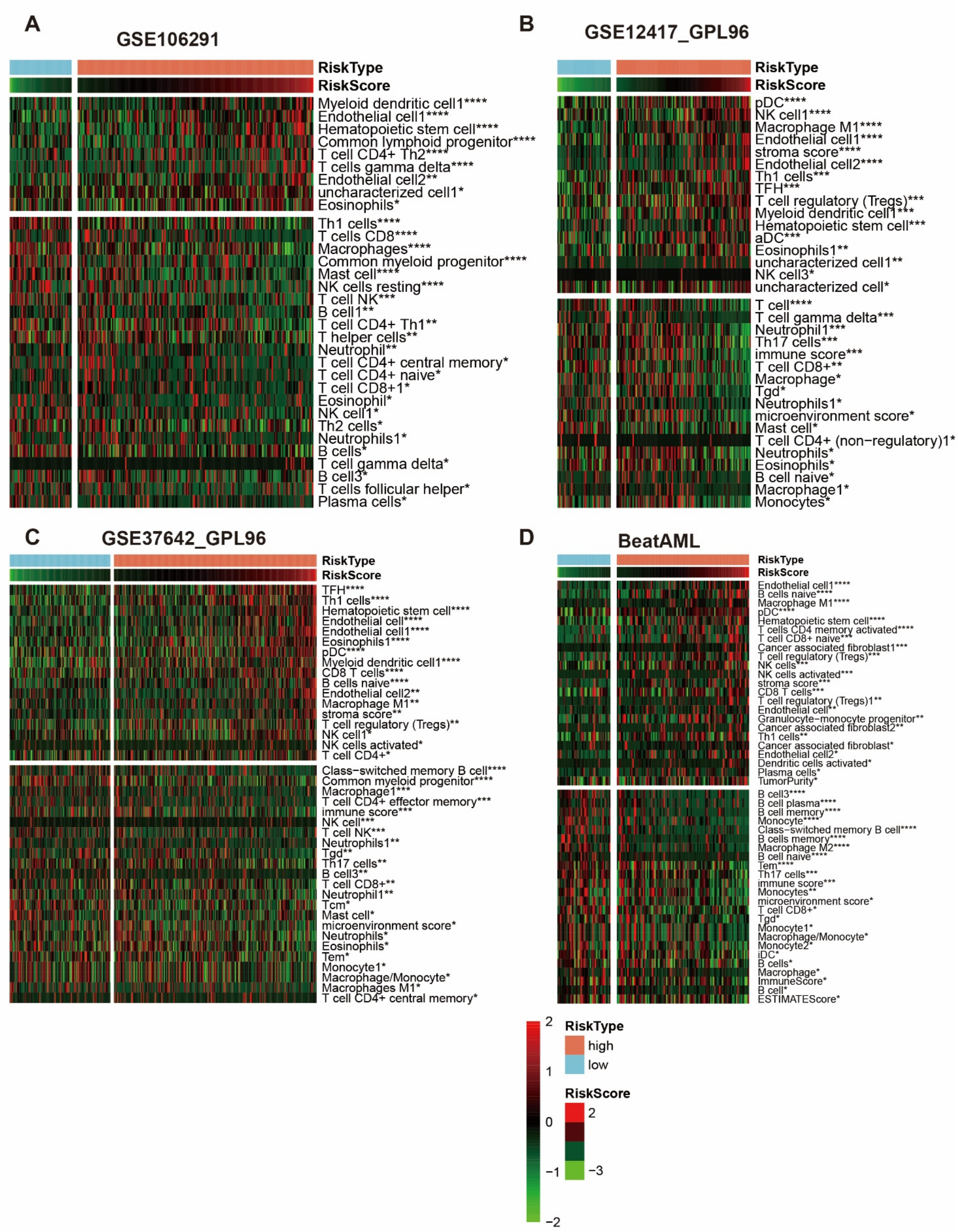

Supplementary Figure 11. The spearman correlation of the level of immune cell infiltration and PAN2RS was calculated in GSE37642, GSE106291, GSE12417 and BeatAML cohorts. The two- sided p value < 0.05 was

considered of significance for all statistical analyses and shown as * p < 0.05, ** p<0.01, *** p < 0.001 and **** p < 0.0001.

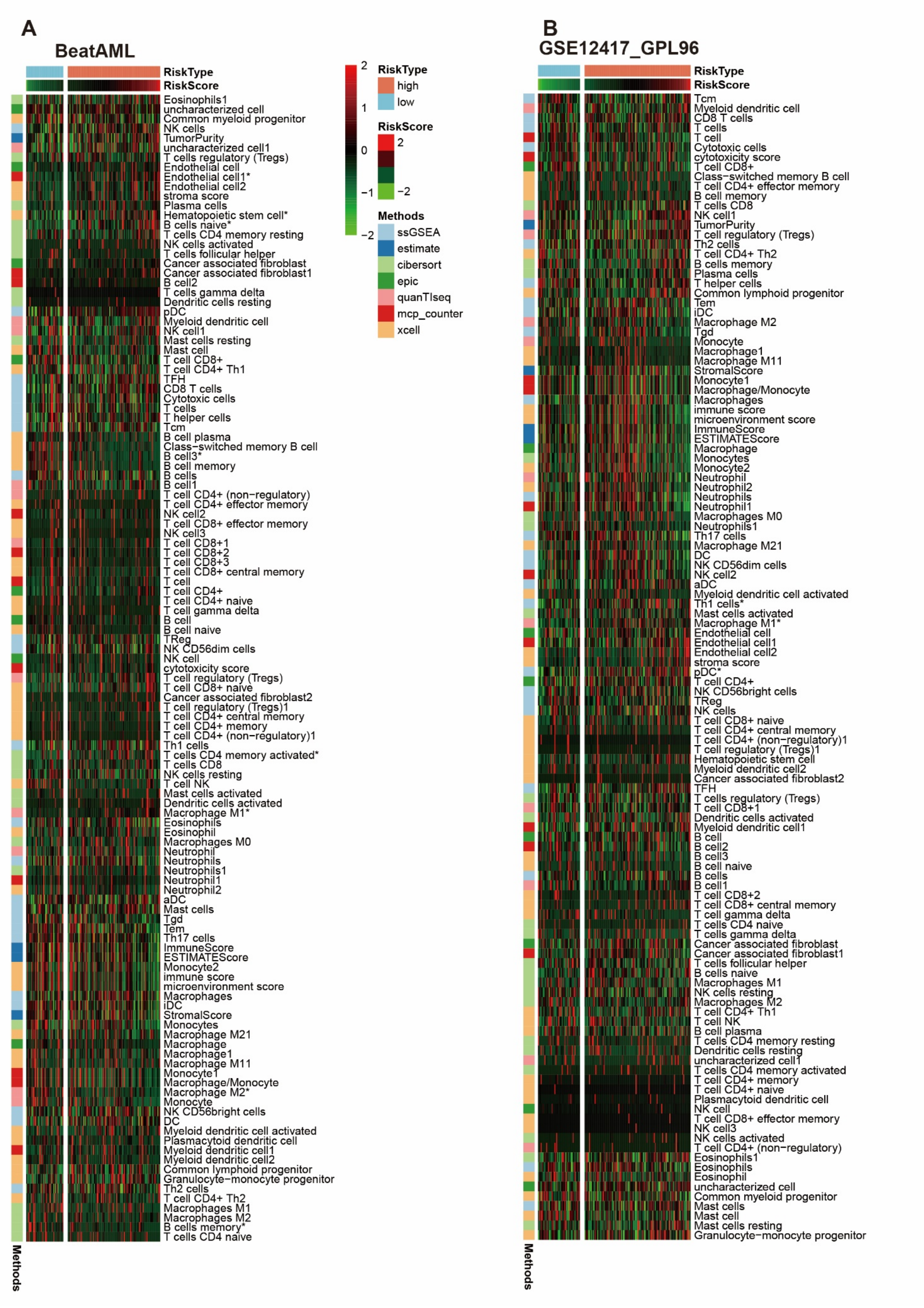

Supplementary Figure 12. The level of immune cell infiltration in the high and low PAN2RS groups were investigated with Wilcoxon sum test in GSE12417 and BeatAML cohorts. The two- sided p value < 0.05 was considered of significance for all statistical analyses and shown as * $p < 0.05$, ** $p<0.01$, *** $p < 0.001$ and **** $p < 0.0001$.

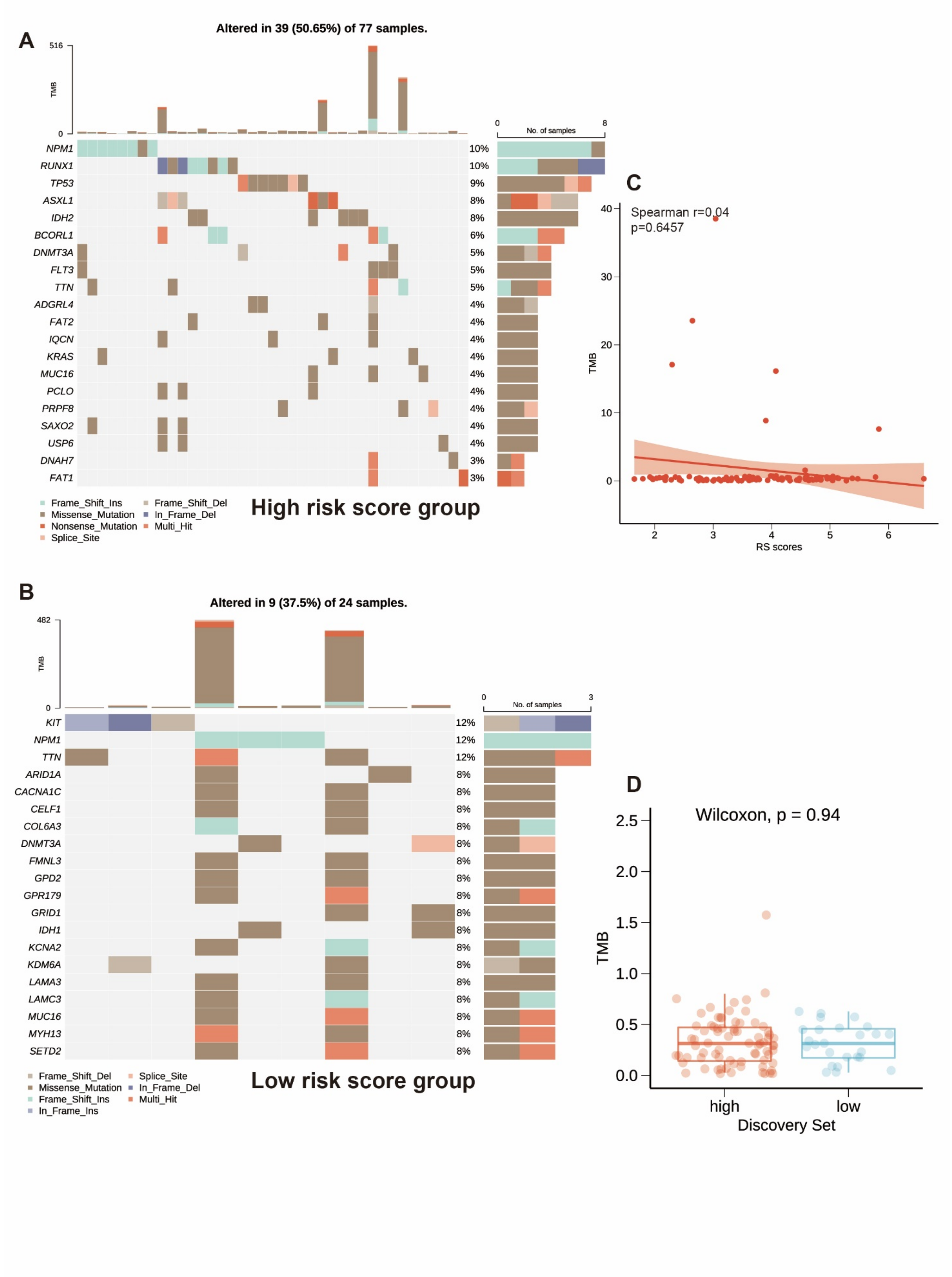

Supplementary Figure 13. A-B. With the mutation data in TCGA-LAML cohort, the gene mutation characteristic in high PAN2RS group (A) and low PAN2RS group (B) was investigated, and the top 20 of the mutated genes were

focused and displayed. C. The correlation between PAN2RS and TMB in the TCGA-LAML cohort was calculated. D. The difference of TMB between the patients with high and low PAN2RS was assessed.

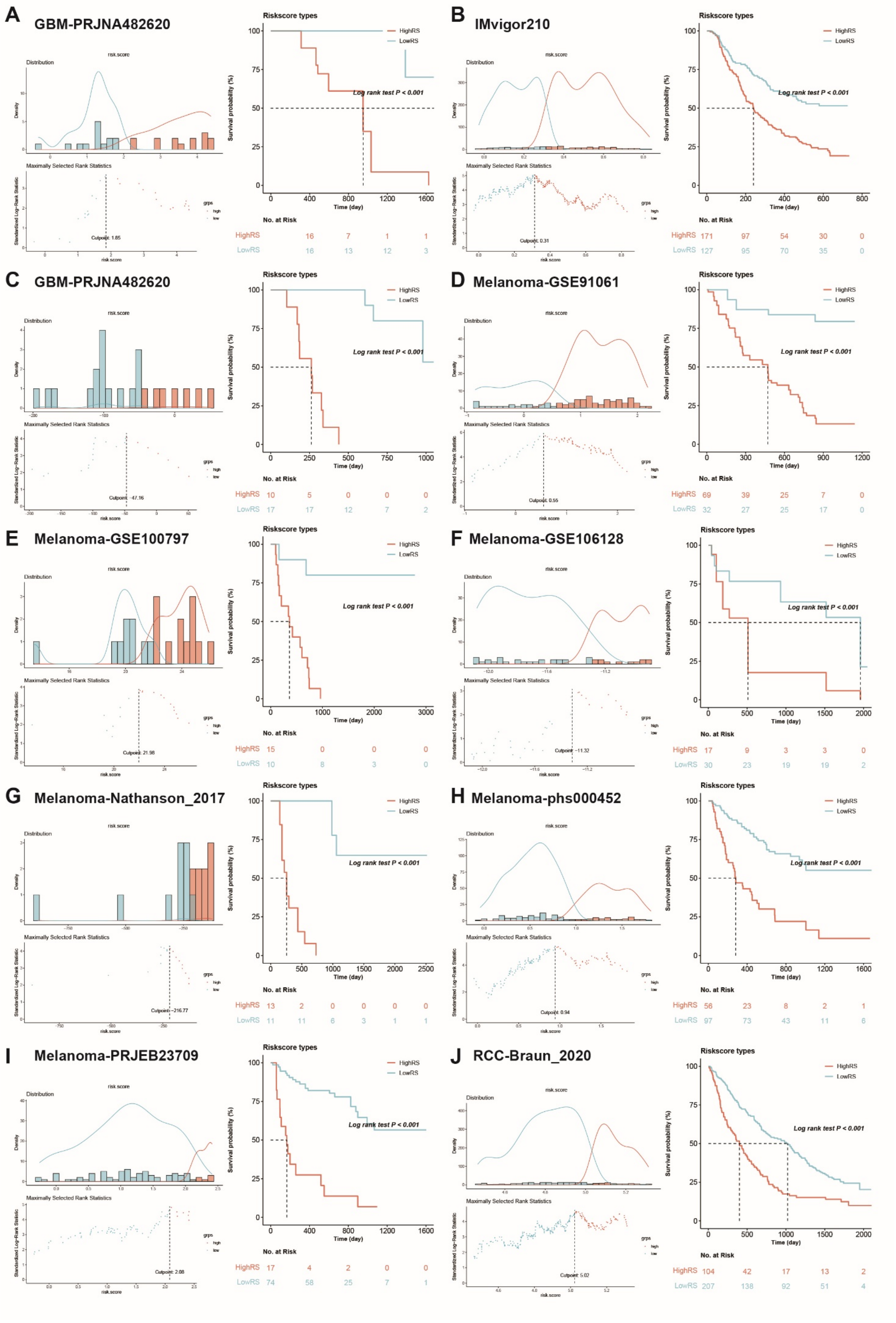
A GBM-PRJNA482620
Riskscore types
HighRS
LowRS
Log rank test P < 0.001
Survival probability (%)
Time (day)
No. at Risk
HighRS 16 7 1 1
LowRS 16 13 12 3
Distribution
Maximally Selected Rank Statistics
Cutpoint: 1.85
B IMvigor210
Riskscore types
Log rank test P < 0.001
No. at Risk
HighRS 171 97 54 30 0
LowRS 127 95 70 35 0
Cutpoint: 0.31
C GBM-PRJNA482620
Riskscore types
Log rank test P < 0.001
No. at Risk
HighRS 10 5 0 0 0
LowRS 17 17 12 7 2
Cutpoint: -47.16
D Melanoma-GSE91061
Riskscore types
Log rank test P < 0.001
No. at Risk
HighRS 69 39 25 7 0
LowRS 32 27 25 17 0
Cutpoint: 0.55
E Melanoma-GSE100797
Riskscore types
Log rank test P < 0.001
No. at Risk
HighRS 15 0 0 0
LowRS 10 8 3 0
Cutpoint: 21.98
F Melanoma-GSE106128
Riskscore types
Log rank test P < 0.001
No. at Risk
HighRS 17 9 3 3 0
LowRS 30 23 19 19 2
Cutpoint: -11.32
G Melanoma-Nathanson_2017
Riskscore types
Log rank test P < 0.001
No. at Risk
HighRS 13 2 0 0 0 0
LowRS 11 11 6 3 1 1
Cutpoint: -216.77
H Melanoma-phs000452
Riskscore types
Log rank test P < 0.001
No. at Risk
HighRS 56 23 8 2 1
LowRS 97 73 43 11 6
Cutpoint: 0.94
I Melanoma-PRJEB23709
Riskscore types
Log rank test P < 0.001
No. at Risk
HighRS 17 4 2 0 0
LowRS 74 58 25 7 1
Cutpoint: 2.08
J RCC-Braun_2020
Riskscore types
Log rank test P < 0.001
No. at Risk
HighRS 104 42 17 13 2
LowRS 207 138 92 51 4
Cutpoint: 5.02

Supplementary Figure 14. A-J. Based on PAN2RS, the patients with immunotherapy treatment were divided into high and low groups, and the patients with high PAN2RS all had worse survival time in all ten immunotherapy cohorts.

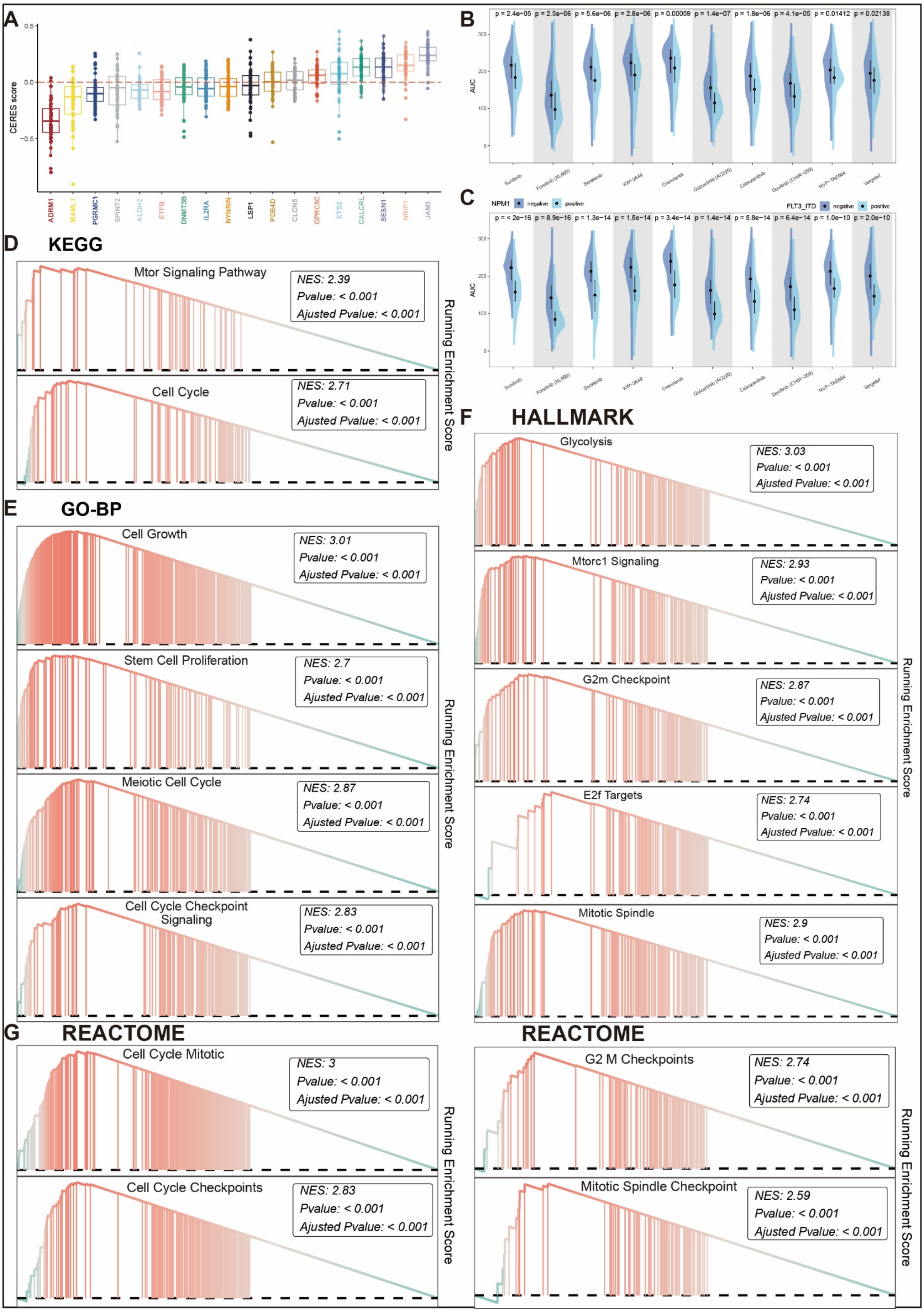

Supplementary Figure 15. A. The CERES scores of 19 signature genes were calculated. B-C. A number of drugs as

positive controls.The drugs were sunitinib, foretinib, sorafenib, KW-2449, crenolanib, quizartinib, cabozantinib, dovitinib, NVP-TAE684 and vargetef. The AUC of the drug between mutation type and wild type was compared. D-G. GSEA results based on KEGG, CO-BP, REACTOME, and HALLMARK gene sets showed high levels of biological signaling such as cell cycle-related pathways, glycolysis, and mTOR1 signaling in the high PAN2RS AML group, explaining the potential of these drugs to treat patients with high PAN2RS.

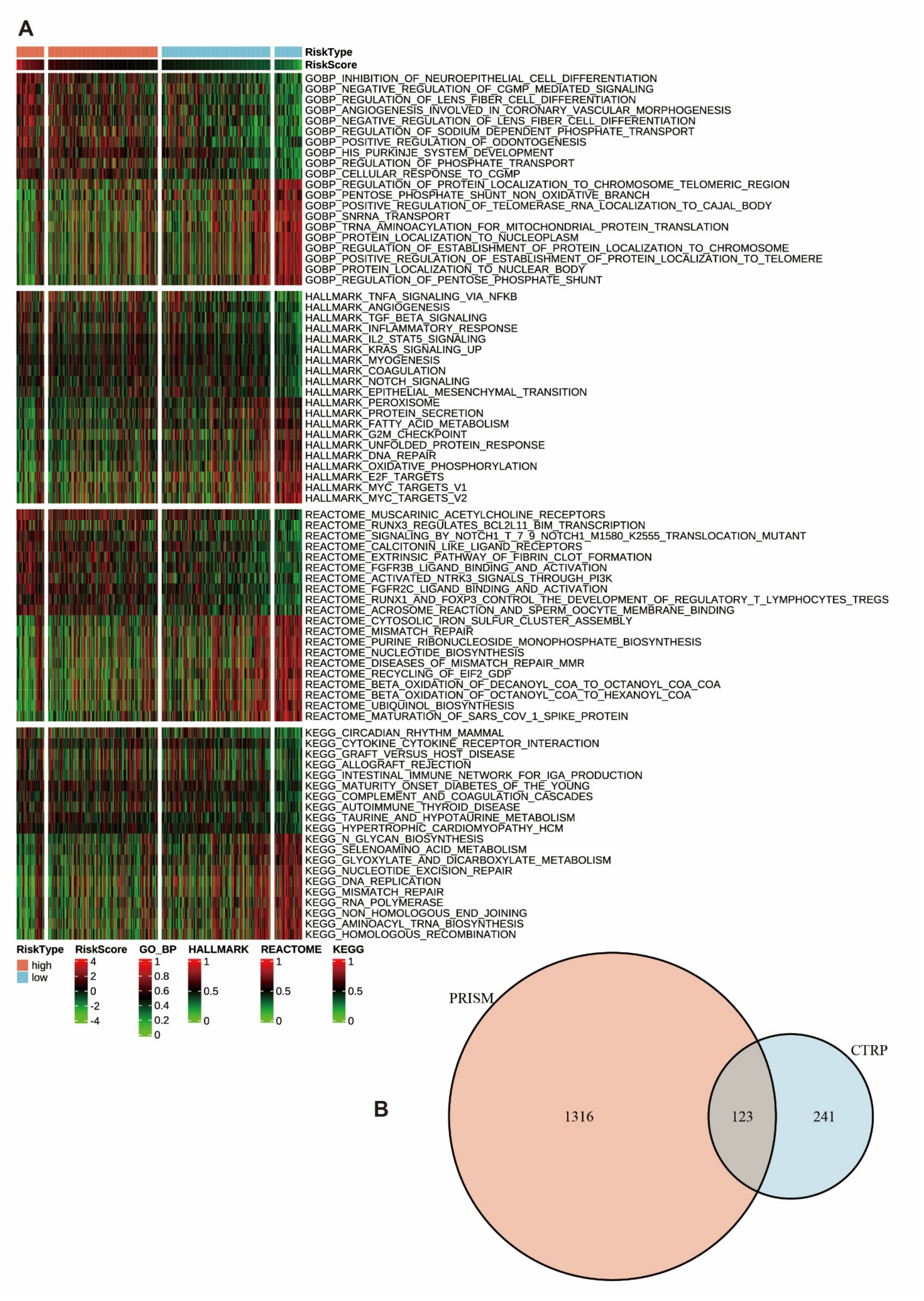

Supplementary Figure 16. A. With the GSVA algorithm, the difference of the signal pathways in GO BP, HALLMARK, REACTOME, and KEGG, were found between the high and low PAN2RS groups. B. A venn

diagram for summarizing included compounds from CTRP and PRISM datasets.

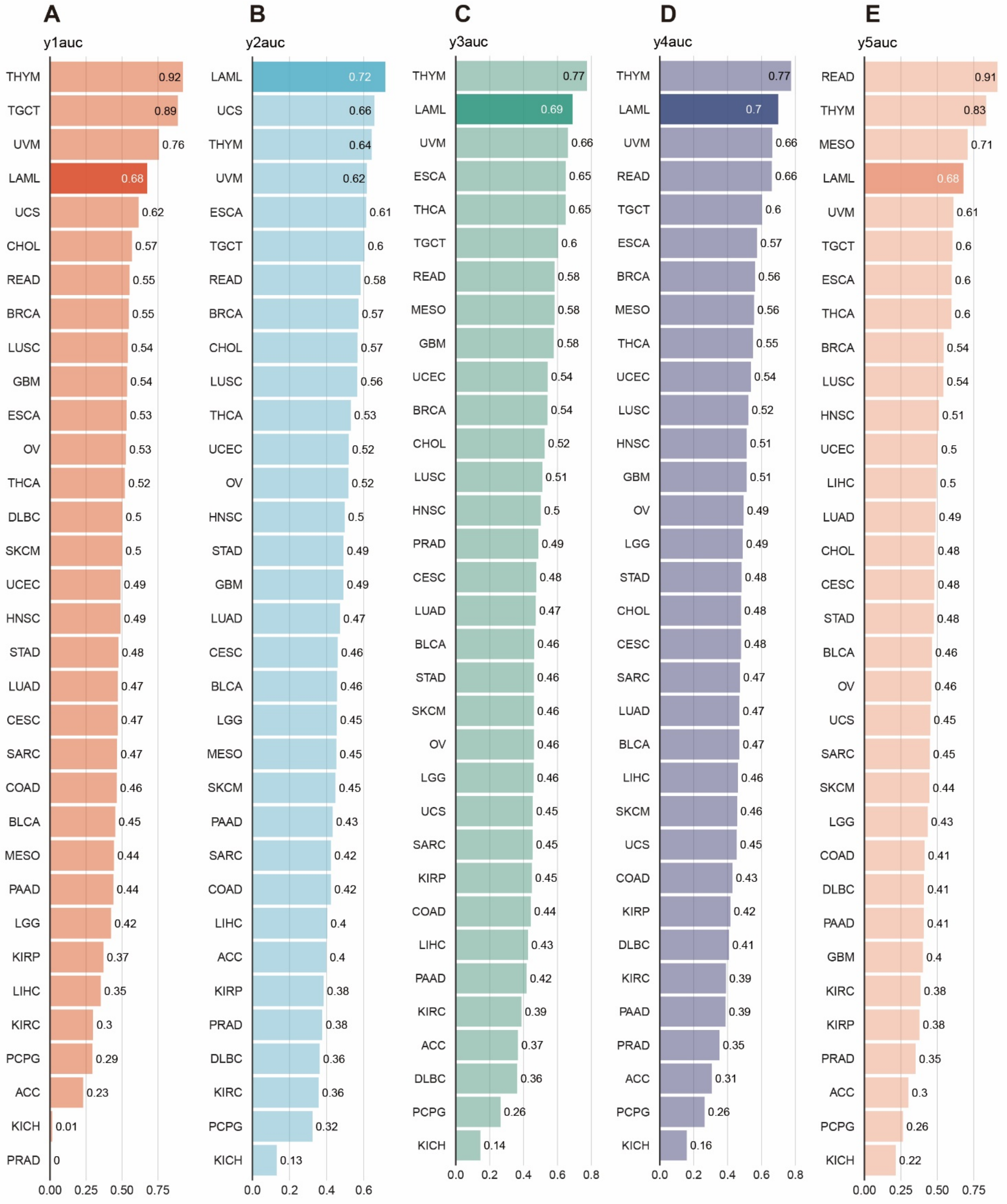

Supplementary Figure 17. A-E. The 1-year, 2-year, 3-year, 4-year and 5-year AUC of the PANoptosis signature were calculated across the cancer types.

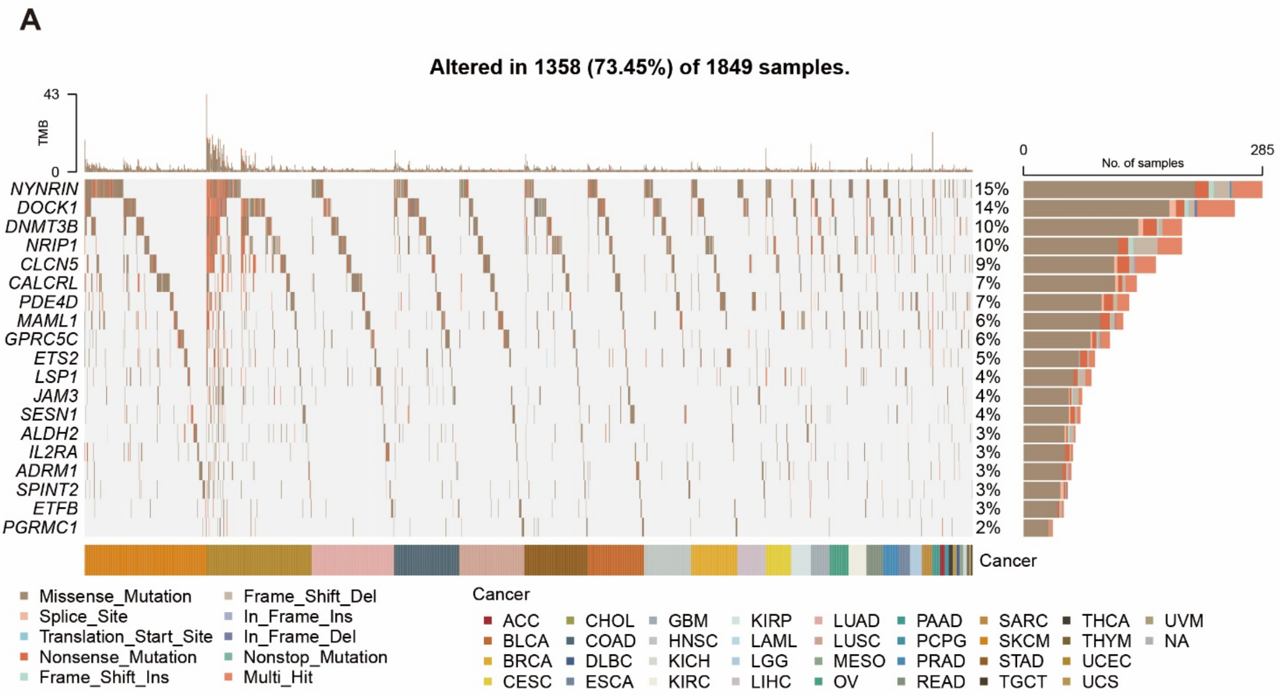

Supplementary Figure 18. The somatic mutation of the 19 signature genes was explored across multiple cancer types. The mainly type of the mutation was missense mutation.

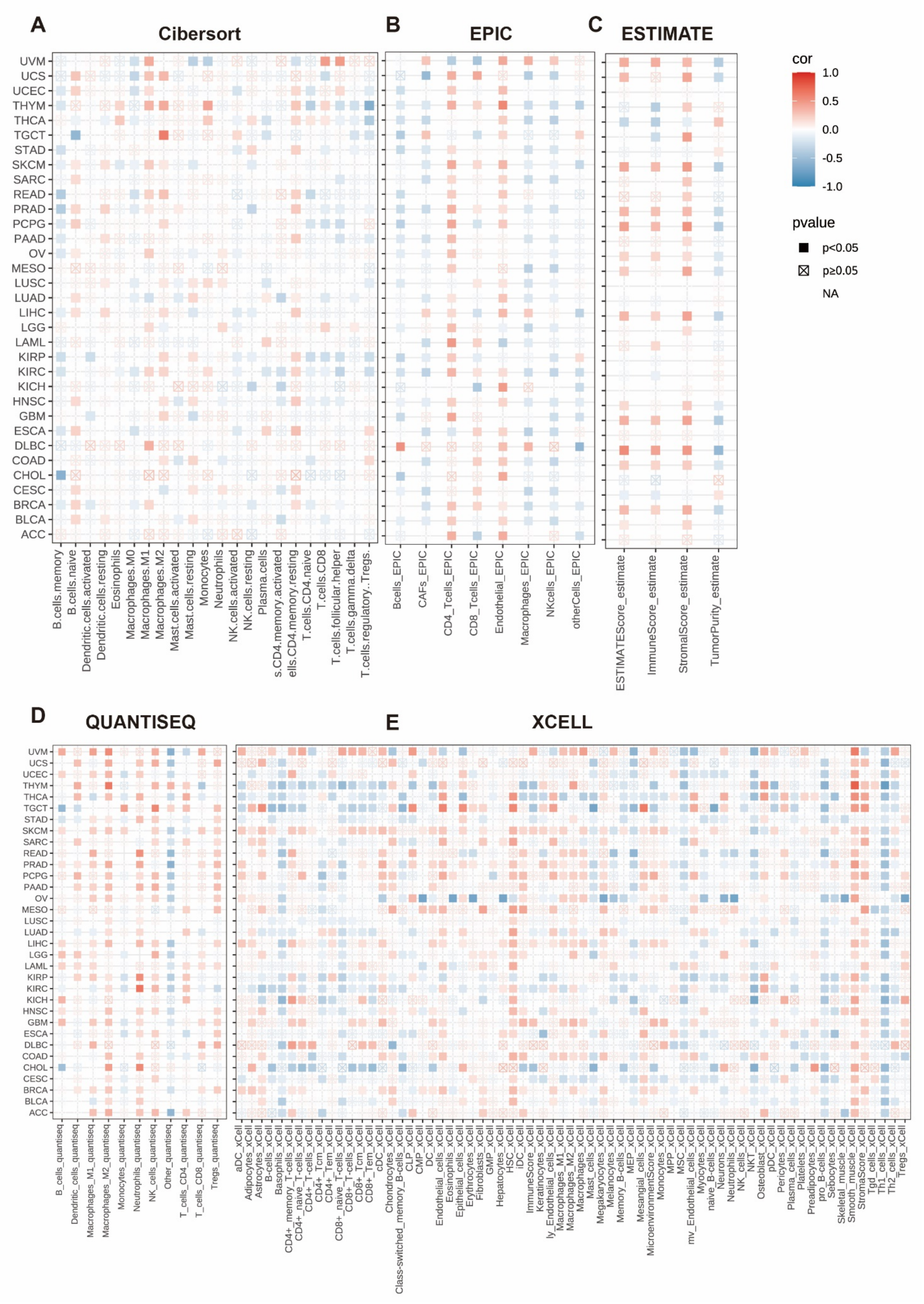

Supplementary Figure 19. We examined the correlation of the PAN2RS and the immune cell infiltration quantified

by five algorithms including CIBERSORT, EPIC, ESTIMATE, QUANTISEQ and xCell. The PAN2RS was significantly and positively associated with the infiltration of CD4 T cells, Tregs and M2 macrophages, while negatively associated with the infiltration of NK cells, memory B cells and Th1 cells across cancer types.

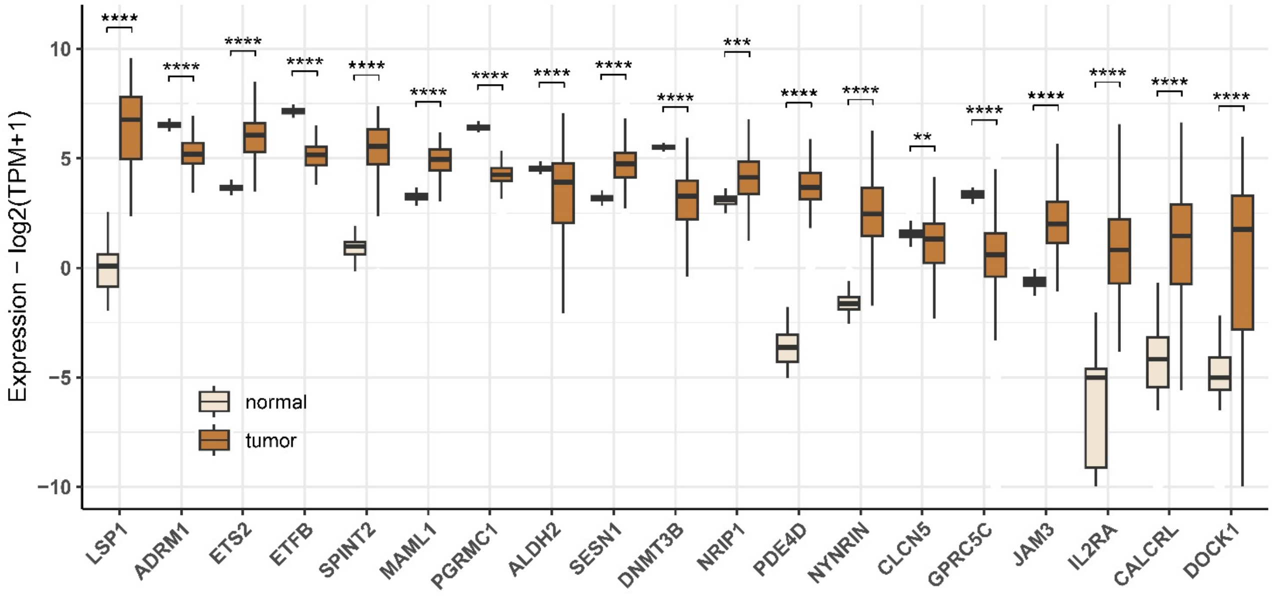

Supplementary figure 20. The expression of the candidate genes in TCGA-LAML and GTEx database. The two-sided p value < 0.05 was considered of significance for all statistical analyses and shown as * p < 0.05, ** p<0.01, *** p < 0.001 and **** p < 0.0001.
