# Supplementary material for: Machine Learning-Based Integrated Analysis of PANoptosis Patterns in Acute Myeloid Leukemia Reveals a Signature Predicting Survival and Immunotherapy
Source: Int J Clin Pract. 2024 Jan 30;2024:5113990. doi: 10.1155/2024/5113990 (PMC10846924; doi:10.1155/2024/5113990)
Supplement: Supplementary Materials — Supplementary Tables provide a helpful elucidation of the researched genes and their background, as well as the sources and comparison of the data. Supplementary Figures 1 to 20 provide a more detailed description of the origin of this model and the advantages and disadvantages compared to other models. They can help elucidate the purpose of this article. [file 5113990.f1.zip › SupplementaryTable_20231227.R.pdf]

Table S1. The genelist of the PANoptosis related genes

GeneSymbol	Type	GeneSymbol	Type	GeneSymbol	Type
ACIN1	Apoptosis	BAK1	Pyroptosis	ADPRHL2	Necroptosis
ADD1	Apoptosis	BAX	Pyroptosis	AIFM1	Necroptosis
AKT1	Apoptosis	CASP1	Pyroptosis	ALKBH7	Necroptosis
AKT2	Apoptosis	CASP3	Pyroptosis	ARHGEF2	Necroptosis
AKT3	Apoptosis	CASP4	Pyroptosis	ASAH1	Necroptosis
APAF1	Apoptosis	CASP5	Pyroptosis	BAX	Necroptosis
APC	Apoptosis	CHMP2A	Pyroptosis	BIRC2	Necroptosis
APIP	Apoptosis	CHMP2B	Pyroptosis	BIRC3	Necroptosis
APPL1	Apoptosis	CHMP4B	Pyroptosis	BOK	Necroptosis
ARHGAP10	Apoptosis	CHMP4C	Pyroptosis	CASP6	Necroptosis
AVEN	Apoptosis	CHMP6	Pyroptosis	CASP8	Necroptosis
BAD	Apoptosis	CHMP7	Pyroptosis	CAV1	Necroptosis
BAK1	Apoptosis	CYCS	Pyroptosis	CFLAR	Necroptosis
BAX	Apoptosis	ELANE	Pyroptosis	CYLD	Necroptosis
BBC3	Apoptosis	GSDMD	Pyroptosis	DNM1L	Necroptosis
BCAP31	Apoptosis	DFNA5	Pyroptosis	FADD	Necroptosis
BCL2	Apoptosis	GZMB	Pyroptosis	FAS	Necroptosis
BCL2L1	Apoptosis	HMGB1	Pyroptosis	FASLG	Necroptosis
BCL2L11	Apoptosis	IL18	Pyroptosis	FZD9	Necroptosis
BID	Apoptosis	IL1A	Pyroptosis	IPMK	Necroptosis
BIRC2	Apoptosis	IL1B	Pyroptosis	IRF3	Necroptosis
BMF	Apoptosis	IRF1	Pyroptosis	ITPK1	Necroptosis
BMX	Apoptosis	IRF2	Pyroptosis	MAP3K5	Necroptosis
C1QBP	Apoptosis	TP53	Pyroptosis	MIR22	Necroptosis
CARD8	Apoptosis	TP63	Pyroptosis	MLKL	Necroptosis
CASP3	Apoptosis			MUTYH	Necroptosis
CASP6	Apoptosis			NLRP6	Necroptosis
CASP7	Apoptosis			NUPR1	Necroptosis
CASP8	Apoptosis			OGT	Necroptosis
CASP9	Apoptosis			PARP1	Necroptosis
CD14	Apoptosis			PELI1	Necroptosis
CDH1	Apoptosis			PGAM5	Necroptosis
CDKN2A	Apoptosis			PPIF	Necroptosis
CFLAR	Apoptosis			PYGL	Necroptosis
CLSPN	Apoptosis			RBCK1	Necroptosis
CTNNB1	Apoptosis			RIPK1	Necroptosis
CYCS	Apoptosis			RIPK3	Necroptosis
DAPK1	Apoptosis			RNF31	Necroptosis
DAPK2	Apoptosis			SLC25A4	Necroptosis
DAPK3	Apoptosis			SPATA2	Necroptosis
DBNL	Apoptosis			TLR3	Necroptosis
DCC	Apoptosis			TNF	Necroptosis
DFFA	Apoptosis			TP53	Necroptosis
DFFB	Apoptosis			TRAF2	Necroptosis
DIABLO	Apoptosis			TRPM7	Necroptosis
DNM1L	Apoptosis			YBX3	Necroptosis
DSG1	Apoptosis			ZBP1	Necroptosis
DSG2	Apoptosis				
DSG3	Apoptosis				
DSP	Apoptosis				
DYNLL1	Apoptosis				
DYNLL2	Apoptosis				
E2F1	Apoptosis				

FADD	Apoptosis
FAS	Apoptosis
FASLG	Apoptosis
FNTA	Apoptosis
GAS2	Apoptosis
GSDMD	Apoptosis
DFNA5	Apoptosis
GSN	Apoptosis
GZMB	Apoptosis
H1F0	Apoptosis
HIST1H1A	Apoptosis
HIST1H1C	Apoptosis
HIST1H1D	Apoptosis
HIST1H1E	Apoptosis
HIST1H1B	Apoptosis
HMGB1	Apoptosis
HMGB2	Apoptosis
KPNA1	Apoptosis
KPNB1	Apoptosis
LMNA	Apoptosis
LMNB1	Apoptosis
LY96	Apoptosis
MAGED1	Apoptosis
MAPK1	Apoptosis
MAPK3	Apoptosis
MAPK8	Apoptosis
MAPT	Apoptosis
NMT1	Apoptosis
OCLN	Apoptosis
OMA1	Apoptosis
OPA1	Apoptosis
PAK2	Apoptosis
PKP1	Apoptosis
PLEC	Apoptosis
PMAIP1	Apoptosis
PPP1R13B	Apoptosis
PPP3CC	Apoptosis
PPP3R1	Apoptosis
PRKCD	Apoptosis
PRKCQ	Apoptosis
PSMA1	Apoptosis
PSMA2	Apoptosis
PSMA3	Apoptosis
PSMA4	Apoptosis
PSMA5	Apoptosis
PSMA7	Apoptosis
PSMA8	Apoptosis
PSMB1	Apoptosis
PSMB10	Apoptosis
PSMB2	Apoptosis
PSMB3	Apoptosis
PSMB4	Apoptosis
PSMB5	Apoptosis
PSMB6	Apoptosis
PSMB7	Apoptosis
PSMB8	Apoptosis

PSMB9	Apoptosis
PSMC1	Apoptosis
PSMC2	Apoptosis
PSMC3	Apoptosis
PSMC4	Apoptosis
PSMC5	Apoptosis
PSMC6	Apoptosis
PSMD1	Apoptosis
PSMD10	Apoptosis
PSMD11	Apoptosis
PSMD12	Apoptosis
PSMD13	Apoptosis
PSMD14	Apoptosis
PSMD2	Apoptosis
PSMD3	Apoptosis
PSMD4	Apoptosis
PSMD5	Apoptosis
PSMD6	Apoptosis
PSMD7	Apoptosis
PSMD8	Apoptosis
PSMD9	Apoptosis
PSME1	Apoptosis
PSME3	Apoptosis
PSME4	Apoptosis
PSMF1	Apoptosis
PTK2	Apoptosis
RIPK1	Apoptosis
ROCK1	Apoptosis
RPS27A	Apoptosis
SATB1	Apoptosis
SHFM1	Apoptosis
SEPTIN4	Apoptosis
SFN	Apoptosis
SPTAN1	Apoptosis
STAT3	Apoptosis
STK24	Apoptosis
MST4	Apoptosis
TFDP1	Apoptosis
TFDP2	Apoptosis
TICAM1	Apoptosis
TICAM2	Apoptosis
TJP1	Apoptosis
TJP2	Apoptosis
TLR4	Apoptosis
TNFRSF10A	Apoptosis
TNFRSF10B	Apoptosis
TNFSF10	Apoptosis
TP53	Apoptosis
TP53BP2	Apoptosis
TP63	Apoptosis
TP73	Apoptosis
TRADD	Apoptosis
TRAF2	Apoptosis
UACA	Apoptosis
UBA52	Apoptosis
UBB	Apoptosis

UBC	Apoptosis
UNC5A	Apoptosis
UNC5B	Apoptosis
VIM	Apoptosis
XIAP	Apoptosis
YWHAB	Apoptosis
YWHAE	Apoptosis
YWHAG	Apoptosis
YWHAH	Apoptosis
YWHAQ	Apoptosis
YWHAZ	Apoptosis

Table S2. The summary of the immune related genes

Immune checkpoint genes

Gene	Type	Function
BTLA	Receptor	Inhibitory
CD27	Receptor	Stimulatory
CD40	Receptor	Stimulatory
CTLA4	Receptor	Inhibitory
EDNRB	Receptor	Inhibitory
HAVCR2	Receptor	Inhibitory
ICOS	Receptor	Stimulatory
IL2RA	Receptor	Stimulatory
KIR2DL1	Receptor	Inhibitory
KIR2DL3	Receptor	Inhibitory
LAG3	Receptor	Inhibitory
PDCD1	Receptor	Inhibitory
TIGIT	Receptor	Inhibitory
TLR4	Receptor	Stimulatory
TNFRSF14	Receptor	Stimulatory
TNFRSF18	Receptor	Stimulatory
TNFRSF4	Receptor	Stimulatory
TNFRSF9	Receptor	Stimulatory
ARG1	Others	Inhibitory
ENTPD1	Others	Stimulatory
GZMA	Others	Stimulatory
HMGB1	Others	Stimulatory
PRF1	Others	Stimulatory
HLA-A	Antigen pre	MHC
HLA-B	Antigen pre	MHC
HLA-C	Antigen pre	MHC
HLA-DPA1	Antigen pre	MHC
HLA-DPB1	Antigen pre	MHC
HLA-DQA	Antigen pre	MHC
HLA-DQB	Antigen pre	MHC
HLA-DQB	Antigen pre	MHC
HLA-DRA	Antigen pre	MHC
MICA	Antigen pre	MHC
MICB	Antigen pre	MHC
CCL5	Ligand	Stimulatory
CD40LG	Ligand	Stimulatory
CD70	Ligand	Stimulatory
CX3CL1	Ligand	Stimulatory
CXCL10	Ligand	Stimulatory
CXCL9	Ligand	Stimulatory
IFNA1	Ligand	Stimulatory
IFNA2	Ligand	Stimulatory
IFNG	Ligand	Stimulatory
IL10	Ligand	Inhibitory
IL12A	Ligand	Stimulatory
IL13	Ligand	Inhibitory
IL1A	Ligand	Stimulatory
IL1B	Ligand	Stimulatory
IL2	Ligand	Stimulatory
IL4	Ligand	Inhibitory
TNF	Ligand	Stimulatory
TNFSF4	Ligand	Stimulatory

The signature of the anti-tumor cycle.

Gene	Steps	Direction	ImmuneCellType
IL10	1	positive	Multiple
TGFB1	1	positive	Multiple
HMGB1	1	positive	Multiple
ANXA1	1	positive	Multiple
CALR	1	positive	Multiple
CXCL10	1	positive	Multiple
PDIA3	1	positive	Multiple
HSPA1A	1	positive	Multiple
HSPA1B	1	positive	Multiple
HSPA2	1	positive	Multiple
HSPA8	1	positive	Multiple
HSPA4	1	positive	Multiple
HSPA14	1	positive	Multiple
HSPA5	1	positive	Multiple
HSPA6	1	positive	Multiple
HSPA9	1	positive	Multiple
HSPA13	1	positive	Multiple
HSPA7	1	positive	Multiple
HSPA8	1	positive	Multiple
HSPA12A	1	positive	Multiple
HSPA12B	1	positive	Multiple
HSP90AA1	1	positive	Multiple
HSP90AB1	1	positive	Multiple
HSP90B1	1	positive	Multiple
IFNA2	1	positive	Multiple
IFNA1	1	positive	Multiple
IFNA13	1	positive	Multiple
IFNA6	1	positive	Multiple
IFNA21	1	positive	Multiple
IFNA4	1	positive	Multiple
IFNA8	1	positive	Multiple
IFNA5	1	positive	Multiple
IFNA7	1	positive	Multiple
IFNA14	1	positive	Multiple
IFNA16	1	positive	Multiple
IFNA10	1	positive	Multiple
IFNA17	1	positive	Multiple
IFNB1	1	positive	Multiple
IFNE	1	positive	Multiple
IFNW1	1	positive	Multiple
TNF	2	positive	Multiple
IL1A	2	positive	Multiple
IL1B	2	positive	Multiple
IFNA2	2	positive	Multiple
IFNA1	2	positive	Multiple
IFNA13	2	positive	Multiple
IFNA6	2	positive	Multiple
IFNA21	2	positive	Multiple
IFNA4	2	positive	Multiple
IFNA8	2	positive	Multiple
IFNA5	2	positive	Multiple
IFNA7	2	positive	Multiple

TNFSF9	Ligand	Stimulatory
VEGFA	Ligand	Inhibitory
VEGFB	Ligand	Inhibitory
CD28	Co-stimula	Stimulatory
CD80	Co-stimula	Stimulatory
ICOSLG	Co-stimula	Stimulatory
BTN3A1	Co-inhibito	Stimulatory
BTN3A2	Co-inhibito	Stimulatory
CD274	Co-inhibito	Inhibitory
CD276	Co-inhibito	Inhibitory
PDCD1LG	Co-inhibito	Inhibitory
SLAMF7	Co-inhibito	Inhibitory
VTCN1	Co-inhibito	Inhibitory
ICAM1	Cell adhesi	Stimulatory
ITGB2	Cell adhesi	Stimulatory
SELP	Cell adhesi	Stimulatory

IFNA14	2	positive	Multiple
IFNA16	2	positive	Multiple
IFNA10	2	positive	Multiple
IFNA17	2	positive	Multiple
CD40LG	2	positive	Multiple
CD40	2	positive	Multiple
NT5C	2	positive	Multiple
HMGB1	2	positive	Multiple
TLR1	2	positive	Multiple
TLR2	2	positive	Multiple
TLR3	2	positive	Multiple
TLR4	2	positive	Multiple
TLR5	2	positive	Multiple
TLR6	2	positive	Multiple
TLR7	2	positive	Multiple
TLR8	2	positive	Multiple
TLR9	2	positive	Multiple
TLR10	2	positive	Multiple
HLAA	2	positive	Multiple
B2M	2	positive	Multiple
TAP1	2	positive	Multiple
IL10	2	negative	Multiple
IL4	2	negative	Multiple
IL13	2	negative	Multiple
CD3D	3	positive	Multiple
CD3E	3	positive	Multiple
CD3G	3	positive	Multiple
CD247	3	positive	Multiple
CD28	3	positive	Multiple
TNFRSF9	3	positive	Multiple
TNFSF9	3	positive	Multiple
TNFRSF4	3	positive	Multiple
TNFSF4	3	positive	Multiple
CD27	3	positive	Multiple
CD70	3	positive	Multiple
TNFRSF1[illegible]	3	positive	Multiple
TNFSF14	3	positive	Multiple
CD40	3	positive	Multiple
CD40LG	3	positive	Multiple
TNFRSF1[illegible]	3	positive	Multiple
TNFSF18	3	positive	Multiple
TNFRSF2[illegible]	3	positive	Multiple
TNFSF15	3	positive	Multiple
TNFRSF8	3	positive	Multiple
TNFSF8	3	positive	Multiple
HAVCR1	3	positive	Multiple
TIMD4	3	positive	Multiple
SLAMF7	3	positive	Multiple
SLAMF6	3	positive	Multiple
SLAMF1	3	positive	Multiple
SLAMF9	3	positive	Multiple
SLAMF8	3	positive	Multiple
CD2	3	positive	Multiple
CD48	3	positive	Multiple
CD58	3	positive	Multiple
CD226	3	positive	Multiple

ICOS	3	positive	Multiple
ICOSLG	3	positive	Multiple
KLRK1	3	positive	Multiple
MICA	3	positive	Multiple
MICB	3	positive	Multiple
RAET1E	3	positive	Multiple
RAET1G	3	positive	Multiple
CRTAM	3	positive	Multiple
CADM1	3	positive	Multiple
CTLA4	3	negative	Multiple
PDCD1	3	negative	Multiple
PDCD1LG	3	negative	Multiple
CD274	3	negative	Multiple
CD160	3	negative	Multiple
TNFRSF14	3	negative	Multiple
BTLA	3	negative	Multiple
VSIR	3	negative	Multiple
LAIR1	3	negative	Multiple
HAVCR1	3	negative	Multiple
HAVCR2	3	negative	Multiple
LGALS9	3	negative	Multiple
TIMD4	3	negative	Multiple
CD244	3	negative	Multiple
CD48	3	negative	Multiple
TIGIT	3	negative	Multiple
NECTIN3	3	negative	Multiple
LAG3	3	negative	Multiple
IL2	3	positive	Multiple
IL12A	3	positive	Multiple
IL12B	3	positive	Multiple
CXCR5	4	positive	B cell
CXCL13	4	positive	B cell
CCL24	4	positive	Basophil
CCL26	4	positive	Basophil
CCL19	4	positive	CD4 T cell
CX3CL1	4	positive	CD4 T cell
CXCL16	4	positive	CD4 T cell
CCR5	4	positive	CD8 T cell
CXCR3	4	positive	CD8 T cell
CXCL10	4	positive	CD8 T cell
CXCL9	4	positive	CD8 T cell
CCL20	4	positive	CD8 T cell
CXCL11	4	positive	CD8 T cell
CX3CL1	4	positive	CD8 T cell
CXCL16	4	positive	CD8 T cell
CCR7	4	positive	Dendritic cell
CCL3	4	positive	Dendritic cell
CCL4	4	positive	Dendritic cell
CCL5	4	positive	Dendritic cell
CCL21	4	positive	Dendritic cell
CCL11	4	positive	Eosinophil
CCL24	4	positive	Eosinophil
CCL26	4	positive	Eosinophil
CSF1	4	positive	Macrophage
CCL2	4	positive	Macrophage
CCL3	4	positive	Macrophage

CCL4	4	positive	Macrophage
CCL5	4	positive	Macrophage
CXCR2	4	positive	MDSC
CXCL5	4	positive	MDSC
CCL2	4	positive	Monocyte
CCL7	4	positive	Monocyte
CX3CL1	4	positive	Monocyte
CXCL1	4	positive	Neutrophil
CXCL2	4	positive	Neutrophil
CXCL3	4	positive	Neutrophil
CXCL8	4	positive	Neutrophil
CXCL6	4	positive	Neutrophil
CXCL5	4	positive	Neutrophil
CXCR3	4	positive	NK cell
CXCL10	4	positive	NK cell
CXCL9	4	positive	NK cell
CCL3	4	positive	NK cell
CCL4	4	positive	NK cell
CCL5	4	positive	NK cell
CXCL11	4	positive	NK cell
CX3CL1	4	positive	NK cell
CXCR5	4	positive	T cell
CCR7	4	positive	T cell
CXCL9	4	positive	T cell
CCL3	4	positive	T cell
CCL4	4	positive	T cell
CCL5	4	positive	T cell
CCL19	4	positive	T cell
CCL21	4	positive	T cell
CX3CL1	4	positive	T cell
CXCL13	4	positive	T cell
CXCR3	4	positive	TH1 cell
CXCL10	4	positive	TH1 cell
CXCL9	4	positive	TH1 cell
CXCL11	4	positive	TH1 cell
CCR6	4	positive	TH17 cell
CCL20	4	positive	TH17 cell
CXCL12	4	positive	TH17 cell
CXCR4	4	positive	TH17 cell
CCL1	4	positive	Th2 cell
CCL17	4	positive	Th2 cell
CCL22	4	positive	Th2 cell
CCR6	4	positive	TH22 cell
CCL20	4	positive	TH22 cell
CCR4	4	positive	Treg cell
CCR10	4	positive	Treg cell
CCL1	4	positive	Treg cell
CCL17	4	positive	Treg cell
CCL22	4	positive	Treg cell
CCL28	4	positive	Treg cell
STAT1	5	positive	T cell
IRF5	5	positive	T cell
KLF2	5	positive	T cell
ITGB2	5	positive	T cell
ICAM1	5	negative	T cell
EZH2	5	negative	T cell

DNMT1	5	negative	T cell
VEGFA	5	negative	T cell
EDNRB	5	negative	T cell
CD28	6	positive	Multiple
ICOS	6	positive	Multiple
ICOSLG	6	positive	Multiple
TNFRSF9	6	positive	Multiple
TNFSF9	6	positive	Multiple
CD27	6	positive	Multiple
CD70	6	positive	Multiple
TNFRSF4	6	positive	Multiple
TNFSF4	6	positive	Multiple
TNFSF14	6	positive	Multiple
CD40	6	positive	Multiple
CD40LG	6	positive	Multiple
HLAA	6	positive	Multiple
B2M	6	positive	Multiple
TAP1	6	positive	Multiple
BIRC5	6	positive	Multiple
MDM2	6	positive	Multiple
MAGEA4	6	positive	Multiple
TP53	6	positive	Multiple
PDCD1	6	negative	Multiple
PDCD1LG	6	negative	Multiple
CD274	6	negative	Multiple
CTLA4	6	negative	Multiple
BTLA	6	negative	Multiple
VTCN1	6	negative	Multiple
IFNG	7	positive	Multiple
GZMB	7	positive	Multiple
PRF1	7	positive	Multiple
PDCD1	7	negative	Multiple
SMC3	7	negative	Multiple
VTCN1	7	negative	Multiple
HAVCR2	7	negative	Multiple
MICA	7	negative	Multiple
MICB	7	negative	Multiple
BTLA	7	negative	Multiple
VSIR	7	negative	Multiple
LAG3	7	negative	Multiple
IDO1	7	negative	Multiple
IDO2	7	negative	Multiple
ARG1	7	negative	Multiple
ARG2	7	negative	Multiple
NOS1	7	negative	Multiple
NOS2	7	negative	Multiple
NOS3	7	negative	Multiple
TGFB1	7	negative	Multiple
IL10	7	negative	Multiple
CCL28	7	negative	Multiple
CXCL12	7	negative	Multiple
CCL2	7	negative	Multiple
CXCL8	7	negative	Multiple

Table S3. The collection of the ORR across cancers

Tumor	ORR
ACC	0.062
BRCA	0.057
CESC	0.2
COAD_MS	0.39
COAD_MS	0
UCEC	0.13
ESCA	0.11
GBM	0.087
HNSC	0.15
LIHC	0.18
SKCM	0.37
MESO	0.13
UVM	0.036
LUAD	0.17
LUSC	0.17
OV	0.099
PAAD	0
PRAD	0.075
KIRC	0.24
SARC	0.093
BLCA	0.18

Table S4. The AML-related signatures gathered from the published papers (The signatures (PMID=12345678) was developed in this paper)

Number	PMID	signatures	coefficient
0	12345678	CALCRL	0.165201917
0	12345678	NRIP1	-0.04572606
0	12345678	DOCK1	0.000876038
0	12345678	GPRC5C	0.356917447
0	12345678	DNMT3B	0.2205022
0	12345678	ALDH2	0.164058052
0	12345678	NYNRIN	-0.044654
0	12345678	ETS2	0.059592669
0	12345678	LSP1	0.013716468
0	12345678	MAML1	-0.43203219
0	12345678	PGRMC1	0.227522484
0	12345678	CLCN5	0.229219481
0	12345678	ADRM1	-0.20361338
0	12345678	PDE4D	0.270212408
0	12345678	SPINT2	0.15869172
0	12345678	ETFB	0.234474761
0	12345678	SESN1	0.125186518
0	12345678	JAM3	-0.00137712
0	12345678	IL2RA	0.113519502
1	34135913	BAG3	0.1084
1	34135913	CALCOCO2	-0.3836
1	34135913	CAMKK2	-0.5617
2	34135913	CANX	-0.2402
2	34135913	DAPK1	0.5119
2	34135913	P4HB	0.2899
3	34135913	TSC2	-0.6286
3	34135913	ULK1	-0.3645
3	34784264	ACSL6	-0.16427034
3	34784264	G3BP1	-0.05745754
3	34784264	CD44	-0.01260057
3	34784264	FH	0.011883347
3	34784264	GPX4	0.040188616
3	34784264	CISD1	0.070266332
3	34784264	SESN2	0.073905513
3	34784264	LPCAT3	0.074774143
3	34784264	AIFM2	0.075162582
4	34784264	ACSL5	0.115640755
4	34784264	HSPB1	0.184767081
4	34784264	SOCS1	0.287328369
5	35111195	CHAC1	0.476551252
5	35111195	CISD1	0.291108125
5	35111195	DPP4	0.242536399
5	35111195	GPX4	0.02380646
5	35111195	AIFM2	0.581670513
5	35111195	SQLE	0.080298868
6	35111195	PGD	0.01518642
6	35111195	ACSF2	0.413485022
6	35127715	LGALS1	0.154

6	35127715	DLL3	-0.0482	
6	35127715	ZFPM2	0.0683	
6	35127715	LHX6	0.0279	
6	35127715	MXRA5	-0.0111	
6	35127715	TMEM56	-0.0119	
6	35127715	CCL23	0.0113	
6	35127715	FAM155B	0.0308	
6	35127715	ZSCAN4	0.0119	
6	35127715	PCDHB12	-0.0182	
6	35127715	PRINS	-0.0659	
6	35127715	FOXL1	-0.00139	
6	35127715	ASTN1	-0.00639	
6	35127715	HMX2	0.0638	
6	35127715	HRASLS	0.0428	
7	34282207	ENO3	1	
7	34282207	F3	0.55	
7	34282207	CCNA2	0.41	
7	34282207	SLC2A5	0.47	
8	33193652	FLT3	0.261	
8	33193652	CD177	0.327	
8	33193652	TTPAL	0.555	
8	28473658	has-mir-146b	1.652	
8	28473658	hsa-mir-181c	1.838	
8	28473658	has-mir-4786	1.455	
9	35958446	ACVR2A	0.12	
9	35958446	CCL22	1.53	
9	35958446	EBI3	0.168	
10	35958446	EDN1	0.691	
10	35958446	FFAR2	0.066	
10	35958446	HRH1	0.661	
10	35958446	ICOSLG	0.313	
10	35958446	IL10	0.033	
10	35958446	INHBA	0.095	
10	35958446	ITGB3	0.019	
10	35958446	LAMP3	1.103	
10	34869365	USP30	0	0.190627
10	34869365	AC114271.2	0.10995083	
11	34869365	AF064858.8	0.0704641	
11	34869365	RP11-	22L13.1	0.025116
11	34869365	MIR181A1HG	-0.129286	
11	34869365	RP11-544A12.4	-0.0512868	
11	34869365	MIR133A1HG	-0.0383528	
11	32436945	4-Oct	0.815	
11	32436945	POU5F1B	-0.933	
11	32436945	BMI1P1	-0.909	
11	36034801	CASP3	-0.09931534	
11	36034801	ELANE	-0.06690728	
11	36034801	GSDMA	1.296940834	
11	36034801	NOD1	-0.1644051	
11	36034801	PYCARD	0.44027452	
11	36034801	VDR	0.007074287	
11	34579730	AP001266.2	-0.1258	
11	34579730	AC133961.1	0.7448	
11	34579730	AF064858.3	0.1299	
12	34579730	AC007383.2	0.0913	
12	34579730	AC008906.1	0.2202	

12	34579730	AC026771.1	0.1015
12	34579730	KIF26B-AS1	0.3423
12	34777463	ALDOC	0.2737
12	34777463	BATF3	0.1343
13	34777463	COL5A1	0.0762
13	34777463	DR1	0.2795
13	34777463	ELOB	0.6469
13	34777463	HBP1	-0.5492
13	34777463	HK1	-0.4513
13	34777463	KRT14	-0.1064
14	34777463	NOS2	0.074
14	34777463	PSMA2	0.3421
14	34777463	PSMA7	0.8385
14	34777463	PSMB6	-1.1489
14	34777463	PSMC1	0.4238
14	34777463	PTGS1	0.1434
15	34777463	SIAH2	0.3213
15	34777463	SORL1	-0.1447
15	34777463	THBS1	0.0994
15	34777463	UBA52	-0.4732
16	35559049	DNAJB6	-0.65
16	35559049	HSPB1	0.19
16	36292722	YBX3	-0.3676
16	36292722	ZBP1	0.5756
16	36292722	BRAF	-0.6369
16	36292722	ALK	1.7282
17	36292722	BNIP3	-0.4797
17	36292722	CDC37	0.5021
17	34339537	ENPP4	0.153
17	34339537	KLF9	0.2
17	34339537	TUBA4A	0.104
17	34339537	CD247	0.3
17	35854240	DLL3	-0.33351627
17	35854240	NRG1	-0.25591276
17	35854240	CDKN2B	-0.00600483
17	35854240	MMP2	-0.00626355
17	35854240	PPARGC1A	-0.03748634
17	35854240	HOXB7	0.026886551
17	35854240	SNCG	0.182960345
17	35854240	MMP7	0.093034382
17	35854240	BCL2A1	0.067815218
17	29901168	LRRC75A-AS1	-0.2763
17	29901168	FAM30A	0.0822
17	29901168	LINC00926	-0.1418
18	32783661	CASP3	0.01532
18	32783661	CHAF1B	0.0561
18	32783661	KLHL24	0.01959
18	32783661	OPTN	0.06514
18	32783661	VEGFA	-0.05621
18	32783661	VPS37C	0.07141
18	35656299	ATG3	-0.28134
18	35656299	FAM106A	-0.10016
18	35656299	KLHL9	-0.07429
18	35656299	LCMT2	-0.4437
19	35656299	LRRC40	0.1902
19	35656299	LZTR1	0.2291

<table>
<tr><td>19</td><td>35656299</td><td>NCR2</td><td>0.20767</td><td></td></tr>
<tr><td>19</td><td>35656299</td><td>PAFAH2</td><td>0.32442</td><td></td></tr>
<tr><td>19</td><td>35656299</td><td>PCMTD2</td><td>0.14762</td><td></td></tr>
<tr><td>19</td><td>35656299</td><td>PLA2G5</td><td>-0.44088</td><td></td></tr>
<tr><td>19</td><td>35656299</td><td>SCARB1</td><td>0.39573</td><td></td></tr>
<tr><td>19</td><td>35656299</td><td>TK1</td><td>-0.51048</td><td></td></tr>
<tr><td>20</td><td>35656299</td><td>ZNF576</td><td>0.20382</td><td></td></tr>
<tr><td>20</td><td>34966465</td><td>LINC00461</td><td>0.097559</td><td></td></tr>
<tr><td>20</td><td>34966465</td><td>RP11-309M23.1</td><td>0.087081</td><td></td></tr>
<tr><td>20</td><td>34966465</td><td>AC016735.2</td><td>0.107281</td><td></td></tr>
<tr><td>20</td><td>34966465</td><td>RP11-</td><td>61I13.3</td><td>0.100018</td></tr>
<tr><td>20</td><td>34966465</td><td>KIAA0087</td><td>0.072299</td><td></td></tr>
<tr><td>20</td><td>34966465</td><td>RORB-AS1</td><td>0.081708</td><td></td></tr>
<tr><td>20</td><td>34966465</td><td>AC012354.6</td><td>-0.24187</td><td></td></tr>
<tr><td>20</td><td>34630514</td><td>LAR</td><td>P1B</td><td>-0.01322</td></tr>
<tr><td>21</td><td>34630514</td><td>TRNT1</td><td>-0.113572</td><td></td></tr>
<tr><td>21</td><td>34630514</td><td>SMN2</td><td>-0.16828</td><td></td></tr>
<tr><td>21</td><td>34630514</td><td>MRPL28</td><td>0.01175</td><td></td></tr>
<tr><td>22</td><td>34630514</td><td>TRIM21</td><td>0.02097</td><td></td></tr>
<tr><td>22</td><td>34630514</td><td>RPS19BP1</td><td>0.06365</td><td></td></tr>
<tr><td>22</td><td>34630514</td><td>XPO6</td><td>0.08799</td><td></td></tr>
<tr><td>23</td><td>34630514</td><td>TSR2</td><td>0.09969</td><td></td></tr>
<tr><td>23</td><td>34630514</td><td>ISG20</td><td>0.10714</td><td></td></tr>
<tr><td>23</td><td>34630514</td><td>HELZ2</td><td>0.15339</td><td></td></tr>
<tr><td>23</td><td>34630514</td><td>EXOSC4</td><td>0.18212</td><td></td></tr>
<tr><td>23</td><td>34630514</td><td>EIF2AK4</td><td>0.29918</td><td></td></tr>
<tr><td>23</td><td>35569433</td><td>PYCARD</td><td>0.018</td><td></td></tr>
<tr><td>23</td><td>35569433</td><td>BAK1</td><td>0.064</td><td></td></tr>
<tr><td>23</td><td>35569433</td><td>CHMP7</td><td>0.101</td><td></td></tr>
<tr><td>23</td><td>35569433</td><td>NLRP2</td><td>0.115</td><td></td></tr>
<tr><td>23</td><td>35569433</td><td>ZBP1</td><td>0.327</td><td></td></tr>
<tr><td>24</td><td>35569433</td><td>NOD1</td><td>0.101</td><td></td></tr>
<tr><td>24</td><td>35945345</td><td>COL9A2|NPDC1</td><td>-0.384</td><td></td></tr>
<tr><td>24</td><td>35945345</td><td>PLXNC1|SLC24A</td><td>0.438</td><td></td></tr>
<tr><td>25</td><td>35945345</td><td>FZD6|MYO1B</td><td>0.441</td><td></td></tr>
<tr><td>25</td><td>35945345</td><td>TCF4|TAF1C</td><td>0.362</td><td></td></tr>
<tr><td>25</td><td>35945345</td><td>TAF1C|ACSL3</td><td>-0.569</td><td></td></tr>
<tr><td>25</td><td>35945345</td><td>ACSL3|CRTAP</td><td>0.433</td><td></td></tr>
<tr><td>25</td><td>35945345</td><td>ACSL3|IGLL1</td><td>0.378</td><td></td></tr>
<tr><td>25</td><td>35945345</td><td>ACSL3|DNMT3B</td><td>-0.788</td><td></td></tr>
<tr><td>25</td><td>35945345</td><td>CYP2E1|MYO1B</td><td>-0.277</td><td></td></tr>
<tr><td>25</td><td>35945345</td><td>SLC36A1|FSTL1</td><td>-0.465</td><td></td></tr>
<tr><td>25</td><td>35355427</td><td>BST2</td><td>0.184823</td><td></td></tr>
<tr><td>25</td><td>35355427</td><td>MPO</td><td>-0.13986</td><td></td></tr>
<tr><td>25</td><td>35355427</td><td>PSMD3</td><td>1.018944</td><td></td></tr>
<tr><td>25</td><td>35355427</td><td>THBS1</td><td>0.031434</td><td></td></tr>
<tr><td>25</td><td>35355427</td><td>CALR</td><td>-0.250446</td><td></td></tr>
<tr><td>25</td><td>35355427</td><td>OGFR</td><td>-0.765625</td><td></td></tr>
<tr><td>26</td><td>35355427</td><td>CDK6</td><td>-0.592075</td><td></td></tr>
<tr><td>26</td><td>36338716</td><td>CD44</td><td>-0.548</td><td></td></tr>
<tr><td>26</td><td>36338716</td><td>CHAC1</td><td>0.371</td><td></td></tr>
<tr><td>26</td><td>36338716</td><td>CISD1</td><td>0.629</td><td></td></tr>
<tr><td>26</td><td>36338716</td><td>DPP4</td><td>0.399</td><td></td></tr>
<tr><td>26</td><td>36338716</td><td>NCOA4</td><td>-0.849</td><td></td></tr>
<tr><td>27</td><td>36338716</td><td>SAT1</td><td>0.299</td><td></td></tr>
<tr><td>27</td><td>36338716</td><td>SLC7A11</td><td>0.485</td><td></td></tr>
</table>

27	36338716	AIFM2	0.28
27	36338716	G6PD	1.391
28	36338716	ACSF2	0.955
28	32617195	CAMK2A	3.1175
28	32617195	FAT2	-1.3247
28	32617195	GDF9	-1.7172
28	32617195	TCERG1	-0.907
28	32617195	DOC2B	-0.4339
28	32617195	PTGIS	0.4833
28	32617195	PREX1	0.426
28	32617195	DNTTIP1	1.4097
29	32617195	C22orf42	4.6867
29	32617195	CRISPLD1	0.8599
29	33330048	CSK	0.3827
30	33330048	MMP7	0.1383
30	33330048	IKBKG	0.3114
30	33330048	PDCD1	0.1589
30	33330048	PSMA7	0.3812
30	33330048	ISG15	0.2127
30	31278736	AC004223.2	-0.24476
31	31278736	AC067735.1	-7.00E-05
31	31278736	DIRC3-AS1	0.36099
31	31278736	AL355353.1	0.18098
31	31278736	AL645608.1	-0.4052
32	31278736	AC025430.1	0.57742
32	31278736	AF064858.2	0.25425
32	31278736	AL645608.5	0.31499
32	31278736	FP671120.3	-0.20944
32	31278736	AC107398.3	-0.3091
33	35240791	SNORD72	0.452
33	35240791	SNORD38	0.31
33	35240791	U3	-0.203
34	35240791	SNORA73B	0.527
34	35240791	SNORD79	-0.288
34	35240791	SNORA73	-0.712
34	35240791	SNORD12B	-0.608
34	35240791	SNORA74	0.226
34	35240791	SNORD116-12	-0.54
34	35240791	SNORA65	1.227
34	35240791	SNORA14	0.29
34	35240791	snoU13	0.756
34	35240791	SNORA75	0.225
34	35240791	SNORA31	-0.282
34	35962395	ARTN	-0.257
34	35962395	IL5RA	-0.4
34	35962395	LTK	-0.239
34	35962395	SERPINI2	0.534
34	35962395	SLC9A3R2	-0.53
34	35962395	TPPP3	-0.189
35	35756629	ELAINE	0.077
35	35756629	CASP1	0.095
35	35756629	NLRP2	-148
35	32268820	TET3	-0.40986964
35	32268820	S100A4	0.424257154

35	32268820	BATF	0.371192948
36	32268820	CLEC11A	-0.32198846
36	32268820	PTP4A3	0.500628619
36	32268820	SPATS2L	0.186937132
36	32268820	SDHA	0.255904972
36	32268820	ATOX6	-0.24085341
36	30242879	hsa-miR-509	0.914
36	30242879	hsa-miR-542	0.759
36	30242879	hsa-miR-3667	-0.837
36	30242879	hsa-miR-146a	-0.856
37	35924822	SELE	-0.04612562
37	35924822	LGALS1	0.06862245
37	35924822	ITGAX	0.05494352
37	35924822	TMEM200A	0.20978102
37	35924822	SLC25A21	-0.07618051
37	35924822	S100A4	0.14862263
37	35924822	CRIP1	0.03722238
37	36034801	CASP3	-0.09931534
38	36034801	ELANE	-0.06690728
38	36034801	GSDMA	1.296940834
38	36034801	NOD1	-0.1644051
38	36034801	PYCARD	0.44027452
39	36034801	VDR	0.007074287
39	34691250	FAM124B	0.1814
39	34691250	HPDL	0.3634
39	34691250	MPO	0.2312
40	34691250	P2RY1	0.342
40	35528167	MEF2C	1.064015
40	35528167	ENPP2	1.215766
40	35528167	FAM107A	9.98E-05
41	35528167	CD37	1.020001
41	35528167	TNFAIP8L2	1.027994
41	35528167	CASS4	1.192386
41	35854240	DLL3	-0.33351627
41	35854240	NRG1	-0.25591276
42	35854240	CDKN2B	-0.00600483
42	35854240	MMP2	-0.00626355
42	35854240	PPARGC1A	-0.03748634
42	35854240	HOXB7	0.026886551
42	35854240	SNCG	0.182960345
42	35854240	MMP7	0.093034382
42	35854240	BCL2A1	0.067815218
43	34051812	NFKB2	0.04296
43	34051812	NEK9	0.070743
43	34051812	HOXA7	0.055637
43	34051812	ARPC5L	0.748162
43	34051812	FAM30A	0.294823
43	34051812	LOC105371592	0.031033
43	35957912	EBP	0.148491224
43	35957912	CBR1	0.100012929
43	35957912	PLA2G4A	0.097460254
43	35957912	DGKA	0.500714298
43	35957912	PNPLA6	0.027807782

43	35957912	LDLRAP1	0.005424364
44	34276784	MIR133A1HG	-0.556
44	34276784	AL359715.1	-0.44
44	34276784	MIRLET7BHG	0.264
44	34276784	AL356752.1	-0.381
44	36292980	PGAM5	0.3313
44	36292980	SQSTM1	0.2609
44	36292980	ATG9A	0.52
44	36292980	GABARAPL1	-0.194
45	35359394	HPDL	0.019734
45	35359394	CPT1A	0.027628
45	35359394	IDH3A	0.170339
45	35359394	ETFB	0.245829
46	32150667	ALDH2	0.0152
46	32150667	FAM124B	0.017
46	32150667	NYNRIN	0.007
46	32150667	DNMT3B	0.021
46	32150667	DDIT4	0.015
46	32150667	SOCS2	0.0197
46	32150667	ADGRG1	0.039
46	32150667	CALCRL	0.072
46	32150667	NDST1	-0.015
46	32150667	FHL1	0.01
46	36247279	ADAMTS3	-0.0604
46	36247279	DNMT3B	0.1853
47	36247279	NYNRIN	0.0288
47	36247279	SORT1	0.1719
47	36247279	ZFHX3	-0.0633
47	36247279	ZG16B	-0.0373
47	30089916	DNMT3B	0.0874
47	30089916	ZBTB46	-0.0347
47	30089916	NYNRIN	0.00865
47	30089916	ARHGAP22	-0.0138
47	30089916	LAPTM4B	0.00582
47	30089916	MMRN1	0.0258
47	30089916	DPYSL3	0.0284
47	30089916	KIAA0125	0.0196
47	30089916	CDK6	-0.0704
47	30089916	CPXM1	-0.0258
47	30089916	SOCS2	0.0271
47	30089916	SMIM24	-0.0226
47	30089916	EMP1	0.0146
47	30089916	NGFRAP1	0.0465
48	30089916	CD34	0.0338
48	30089916	AKR1C3	-0.0402
48	30089916	GPR56	0.0501
48	36225590	KDM5B	-0.0838
48	36225590	MAP4K1	0.1467
48	36225590	SMURF2	-0.5299
48	36225590	G6PD	0.221
48	36225590	CDK18	0.2415
48	36225590	SOCS1	0.2543
48	36225590	ETS2	0.0803

48	36225590	AKR1B1	0.0411	
48	31645648	DNMT3B	0.189	
49	31645648	GPR56	0.054	
49	31645648	CD34	0.0171	
50	31645648	SOCS2	0.141	
50	31645648	SPINK2	0.109	
50	31645648	FAM30A	0.0516	
50	36033479	ACSF2	0.2283	
50	36033479	SOCS1	0.1089	
50	36033479	MYB	-0.014	
50	36033479	SLC7A11	0.8615	
51	36033479	GPX4	0.0217	
51	36033479	AIFM2	0.4538	
51	36033479	EIF2AK4	0.1988	
51	36105048	MIF	0.40081	
51	36105048	MPO	-0.15201	
51	36105048	DEF6	0.78073	
51	36105048	AVPR1B	-0.45192	
52	36105048	OSM	0.25912	
52	34536536	MRPS11	0.146689465	
52	34536536	SPATS2L	0.178990804	
52	34536536	ETFB	0.22148659	
52	34536536	PTP4A3	0.252213639	
52	34536536	S100A4	0.136878143	
52	36033493	ADAMTS3	-0.086	
52	36033493	CD52	0.18	
53	36033493	CLCN5	0.472	
53	36033493	HAL	-0.356	
53	36033493	ICAM3	0.368	
53	25428263	hsa-miR-9-5p	0.4908	
53	25428263	hsa-miR-155-5p	0.2243	
53	25428263	hsa-miR-203	-0.7187	
53	36113865	CD27	-0.177	
53	36113865	IL2RA	0.26	
53	36113865	CD96	-0.19	
53	36113865	TIMP1	-0.098	
53	36113865	COL18A1	0.19	
53	36113865	HGF	-0.017	
53	36113865	GDF15	-0.196	
53	36113865	CLEC11A	-0.161	
53	32373535	ROBO2	0.2016	
54	32373535	IL1R2	0.1274	
54	32373535	SCNN1B	-0.5365	
54	31739140	LINC01003	-1.1685	
54	31739140	CTD-2234N14	1.1285	
54	31739140	RP1-137K24	-0.8036	
54	31739140	RP11-834C111	-0.6063	
54	29956722	TREM	L2	1.053
54	29956722	SLC7A11	0.426	
54	29956722	NLRP2	0.222	
54	29956722	DDIT4	0.548	
54	29956722	LSP1	-0.771	
54	29956722	CLEC11A	-0.396	

54	35483237	SYTL4	-0.00457
54	35483237	LGALSL	0.058887
55	35483237	XXbac_BPG32J3	0.152172
55	35483237	C10orf128	0.154056
55	35483237	PTP4A3	0.29969
56	35483237	LINC00649	-0.37059
56	35483237	RP1_261D10.2	-0.32933
56	35483237	RP11_225B17.2	-0.18497
56	35483237	CTD_3179P9.2	-0.00997
56	35483237	RP11_284F21.10	0.021922
56	35483237	CTC_264K15.6	0.025426
56	35483237	AL133493.2	0.027398
57	35483237	CTB_171A8.1	0.125765
57	35483237	AP001610.5	0.148469
57	35483237	RP11_567J20.3	0.166323
57	35483237	CTC_548K16.1	0.219562
58	35483237	RP11_234K19.1	0.260962
58	35483237	TRERNA1	0.328309
58	31413100	DNMT3B	0.0874
58	31413100	ZBTB46	-0.0347
58	31413100	NYNRIN	0.00865
58	31413100	ARHGAP22	-0.0138
58	31413100	LAPTM4B	0.00582
58	31413100	MMRN1	0.0258
58	31413100	DPYSL3	0.0284
58	31413100	KIAA0125	0.0196
58	31413100	CDK6	-0.0704
58	31413100	CPXM1	-0.0258
58	31413100	SOCS2	0.0271
58	31413100	SMIM24	-0.0226
58	31413100	EMP1	0.0146
58	31413100	NGFRAP1	0.0465
59	31413100	CD34	0.0338
59	31413100	AKR1C3	-0.0402
59	31413100	GPR56	0.0501
59	31535409	REC8	2.268
59	31535409	ZNF718	1.471
59	31535409	FUT4	-1.443
59	31535409	CD274	1.633
59	31535409	HTR1B	-0.342
59	31535409	F2RL1	-1.227
59	34025649	CALR	-0.0376
59	34025649	HSPA1B	0.0362
59	34025649	APOBEC3G	0.0589
59	34025649	MX1	0.0687
59	34025649	ISG20	0.171
59	34025649	MPO	-0.0483
59	34025649	CCL4	0.134
59	34025649	FGR	0.0032
59	34025649	MIF	0.2294
60	34025649	IGHD5.18	0.0108
60	34025649	IGHV4.39	-0.0794
60	34025649	IGHV5.51	-0.0608

60	34025649	PLXNB2	0.0676
60	34025649	CLEC11A	-0.0324
60	34025649	TRH	-0.0645
61	34025649	IL1R2	0.0939
61	34025649	GZMB	0.0535
61	31015209	CALCRL	1.734
62	31015209	LSP1	1.092
62	31015209	CD109	0.826
63	33381455	CXCR1	0.65
63	33381455	CXCR2	2.01
63	33381455	CXCR3	1.89
63	33381455	CXCR6	0.65
63	36046688	AGRN	-0.391
63	36046688	ETFB	0.827
64	36046688	PLA2G4A	0.236
64	36046688	RYR1	0.65
64	36046688	SIGMAR1	0.404
64	36046688	SOCS1	0.473
64	34083515	TPM2	0.097292
64	34083515	CD82	0.038719
64	34083515	SLC29A2	0.167487
64	34083515	INF2	0.150025
64	34083515	STAR	-0.13354
65	34083515	ACP6	0.04633
65	34083515	IFI6	0.015753
65	34083515	MROH6	0.086738
65	34083515	GSDMD	0.008289
65	36276132	AC093278.2	0.3233
65	36276132	AC133961.1	0.6025
65	36276132	LINC01679	1.2585
65	36276132	LINC02757	0.349
65	31496748	SLC17A7	0.1089
65	31496748	MSX2	0.1107
65	31496748	CDC26	0.319
65	31496748	MSLN	-0.0486
65	31496748	CTSZ	-0.0681
66	31496748	DEFA3	0.0456
66	32637351	SH3TC2-DT	0.006899
66	32637351	AF064858.1	0.00026
67	32637351	AL133353.1	0.016446
67	36172164	ECHDC3	0.0127
67	36172164	RPS6KL1	-6.00E-04
67	36172164	RELL2	0.0256
67	36172164	FAM64A	-0.0823
67	36172164	SPATS2L	0.0611
67	36172164	MEIS3P1	-0.0284
67	36172164	CDCP1	0.0296
67	36172164	CD276	0.0099
67	36172164	IL1R2	0.0033
67	36172164	OLFML2A	0.1069
67	29138577	ACSF2	0.438
67	29138577	CXCR6	-0.077
67	29138577	FAM124B	0.295

67	29138577	FRYL	-0.391
67	29138577	GYPA	-0.289
67	29138577	HBG1	-0.072
67	29138577	MAGOH	0.387
68	29138577	MDH2	-0.106
68	29138577	SLC2A5	0.329
68	29138577	SUCLG1	0.221
68	29138577	TMC5	-0.35
68	35535739	HIVEP3	0.0292
68	35535739	LPCAT3	0.1576
68	35535739	AIFM2	0.1845
69	36102723	IFI27L1	0.286
69	36102723	YIPF6	-0.228
69	36102723	PARVB	0.0569
69	36102723	TRIM32	0.0614
69	36102723	RHOBTB3	-0.0971
69	33986802	STAT1	0.4439
69	33986802	BATF	0.3082
69	33986802	EML4	0.3003
69	32742384	LINC00639	0.20989291
69	32742384	MKRN3-AS1	0.06638359
70	32742384	LINC02060	0.3139191
70	32742384	LINC02133	0.42299789
70	32742384	LINC01726	-0.19338239
70	32742384	LINC00240	-0.2969476
70	32742384	PAX8-AS1	0.73636164
70	32742384	ZNF32-AS1	0.02132678
71	32742384	LARS2-AS1	0.1805946
71	32742384	RASSF8-AS1	0.53198533
71	32742384	ELF3-AS1	0.41975344
71	32742384	WASHC5-AS1	0.33392756
71	32742384	MYB-AS1	0.62016856
71	32742384	FAR1-IT1	0.02508178
71	31892991	CD52	0.0492
71	31892991	CD96	-0.0018
71	31892991	EMP1	0.0131
71	31892991	TSPAN2	0.2058
71	31892991	STAB1	0.0234
72	31892991	MBTPS1	-0.3658
72	32039005	ITGAL	0.177
72	32039005	ITGAM	0.315
72	32039005	HLA-DRB1	0.371
72	32039005	HLA-DRB5	-0.009
72	32039005	FPR1	0.034
73	32039005	CX3CR1	-0.074
73	32039005	TNFRSF1B	0.172
73	32039005	CXCL16	-0.104
73	32039005	CTSB	-0.38
73	32039005	CTSS	-0.201
73	32039005	HLA-DRA	-0.353
73	32039005	P2RY13	0.003
74	32039005	ITGB2	0.038
74	32039005	CEACAM3	-0.051

74	32039005	SLC11A1	-0.034
74	32039005	C5AR1	-0.049
74	32039005	ADORA3	0.213
75	32039005	GNGT2	0.208
75	36211454	CD74	0.00239
75	36211454	PLXNB1	-0.13147
75	36211454	THBS1	0.0166
75	36211454	PTK2	-0.11492
75	36211454	UNC93B1	0.01322
75	36211454	PPBP	0.00479
75	36211454	CXCL12	-0.01512
75	36211454	GZMB	0.03528
75	36211454	IFI30	-0.048
75	35601490	LINC00852	0.0256
75	35601490	AL157392.3	0.0278
76	35601490	AC127459.1	0.0946
76	35601490	AC106820.3	0.0024
76	35601490	AC092757.2	1.6278
76	35601490	AC124248.1	0.0054
76	35601490	AC145207.5	8.00E-04
76	35601490	DNAAF4-CCPG	0.007
77	35601490	AC023908.3	0.1977
77	36061194	GPR18	-0.4339
77	36061194	LGALS1	0.3393
77	36061194	AOAH	-0.3897
77	36061194	DNMT3B	0.3038
77	36061194	CBR1	0.2168
77	36061194	ANKRD55	0.3566
77	36061194	SIRPB2	-0.3112
77	36061194	DPY19L2	-0.2369
77	36061194	IL1R2	0.1176
77	36061194	ST8SIA4	-0.316
77	36061194	DOC2A	-0.2405
77	36061194	SERPINI2	0.4186
77	36061194	GZMB	0.3901
77	36061194	TNNT1	0.1895
78	36061194	SORCS2	0.3065
78	34659343	MYB	-0.14587378
78	34659343	MPO	-0.09235308
78	34659343	TWIST1	-0.08499833
78	34659343	ARL6IP5	0.032888142
79	34659343	TXN2	0.035097965
79	34659343	UCP2	0.050187897
79	34659343	SESN2	0.085314112
79	34659343	ATP13A2	0.091438829
79	30954792	HERC5	-0.81432
80	30954792	ABCA5	0.69895
80	30954792	CYP7A1	0.15915
80	30954792	TMPRSS3	-0.2049
80	30954792	CCDC144NL	0.09788
80	30954792	HIST1H2AB	-0.16725
80	35441668	OPRK1	0.318853119
80	35441668	INHBA	0.011740416

80	35441668	OSM	0.145984597
81	35441668	LDLR	0.005309586
81	35441668	BDKRB1	0.525368748
81	35441668	HRH1	0.24729239
81	35441668	CALCRL	0.195067963
82	35441668	CSF3R	-0.12023751
82	35441668	FFAR2	0.057074719
82	35441668	SRI	0.137806144
82	35441668	SLAMF1	0.097822192
82	35441668	LY6E	0.040768088
82	35441668	DCBLD2	-0.23624335
82	35441668	RTP4	0.042952646
82	35441668	EMP3	0.151341064
82	35441668	CLEC5A	-0.01636758
82	32373530	ALDH2	0.018
83	32373530	CYP2E1	-0.103
83	32373530	DNMT3B	0.078
83	32373530	ENPP2	0.032
83	32373530	HAAO	-0.01
83	32373530	ITPKA	-0.039
83	32373530	PAFAH1B2	-0.007
83	32373530	PHGDH	0.04
83	32373530	PSAT1	0.015
83	33117716	PLA2G4A	0.588
84	33117716	HMOX2	1.334
84	33117716	AK1	3.5
84	33117716	SMPD3	1.162
84	33154778	ZC3HAV1L	-0.33298
84	33154778	TFRC	-0.27376
84	33154778	SOCS1	0.10993
84	33154778	ELAVL1	-0.09726
84	33154778	ROBO3	0.22322
85	33154778	UNC93B1	-0.0229
85	33154778	PTPN6	0.246114
85	33154778	IL2RA	0.227133
85	33154778	IL3RA	0.217204
85	36147489	ITGAM	0.14160595
85	36147489	HSPA1B	0.261681018
86	36147489	TNF	-0.23192236
86	36147489	HCP5	0.209743109
86	33536020	CALR	0.34
86	33536020	VSTM1	0.32
87	33536020	PLA2G4A	2.85
87	33536020	GOLGA3	0.35
87	33536020	FNDC3B	0.29
87	34741393	PSAT1	0.271
87	34741393	DDIT4	0.191
87	34741393	ACSL3	0.165
88	34741393	ENPP2	0.131
88	34741393	PGD	0.093
88	34741393	SLC38A1	0.079
88	34741393	CXCL2	0.012
89	34741393	ARNTL	-0.043

89	34741393	HRAS	-0.084
89	34741393	STEAP3	-0.091
89	34741393	HSD17B11	-0.109
89	34741393	PHKG2	-0.51
89	34789073	AKR1C2	0.16
89	34789073	SOCS1	0.383
89	35265597	LINC01436	0.070402
89	35265597	AC073534.2	-0.30330225
89	35265597	LINC02593	-0.13924131

Table S5. The summary of the immunotherapy dataset

DatasetID	Cancer Type	PMID	Therapy	Datasize	R	NR
RCC-Braun_2020	Renal cell carcinoma	32472114	anti-PD-1 and EVEROLIMUS	311	44	237
Melanoma-PRJEB23709	Melanoma	30753825	anti-PD-1 and anti-CTLA-4+anti-PD-1	91	49	42
Melanoma-phs000452	Melanoma	26359337	anti-PD-1	153	63	90
Melanoma-Nathanson_2017	Melanoma	27956380	anti-CTLA-4	24	8	16
Melanoma-GSE106128	Melanoma	29682201	DCs_treated	47	21	14
Melanoma-GSE100797	Melanoma	29170503	ACT	25	10	15
Melanoma-GSE91061	Melanoma	29033130	anti-PD-1	109	20	78
Melanoma-GSE78220	Melanoma	28129544	anti-PD-1	28	15	13
GBM-PRJNA482620	Glioblastoma	30996326	anti-PD-1	34	17	17
IMvigor210	Bladder Cancer	29443960	anti-PD-1	298	68	231

Table S6. The summary of sequence of the genes

IL2RA	FORWARD	CTCTGCCACTCGGAACACAAC
	REVERSE	AGGCTCGCTTGGTCCACTG
JAM3	FORWARD	CAGGTGTGAGGAGCAGGAGATG
	REVERSE	CAGTACAGCAAGGACAACCAGAAC
SESN1	FORWARD	CATTCTCTTGCCTCATTCACATTCG
	REVERSE	CAGTAGTTGCTAACAGAAGGAGGTC
ETFB	FORWARD	AGATCCGAGTGAAGCCTGACAG
	REVERSE	CCGCACAGCCTCCTCCAC
SPINT2	FORWARD	AACGCAGCATCCACGAGAATG
	REVERSE	GGGAGCACTTGGGACAGAGG
PDE4D	FORWARD	GCAACCCAACAAAGCCTCTCC
	REVERSE	GTTCCCTCTCTCGGTCTCCTTG
ADRM1	FORWARD	GGACGACTCGCTTATTCACTTCTG
	REVERSE	CTCCTCATCCTGGTCTGTCTTGG
CLCN5	FORWARD	GAAGCGAAAGACCACCCAGTTG
	REVERSE	TGTGCTCATCCGAGTGTATTCATTG
PGRMC1	FORWARD	CTCAACCTGCTGCTGCTTGG
	REVERSE	TCGTCGTCGTCGCTGTCG
MAML1	FORWARD	GCGGAACAGGAGAAGCAACAG
	REVERSE	GGCACGGCAGCAGAGGAC
LSP1	FORWARD	CACCTGGAGGAGTTGAGTCTGAG
	REVERSE	CCTGGGCTGCTGACATTTCTG
ETS2	FORWARD	GACTTCCCAGGCAGCAACTTG
	REVERSE	TGTCCGCACCGTTCTCAGG
NYNRIN	FORWARD	CCAGAGCACACCGCAGGAG
	REVERSE	CTTGAGCACTGTCCATACCATCTTG
ALDH2	FORWARD	CCACCGTCAATCCGTCCACTG
	REVERSE	CTTCACTGCCTTGTCCACATCTTC
DNMT3B	FORWARD	GTGTGAGGAGTCCATTGCTGTTG
	REVERSE	GCTTCCGCCAATCACCAAGTC
GPRC5C	FORWARD	CTGTTCGCCATCTGCTTCTCTTG
	REVERSE	GCCACTCTGTATTGATGATGACCTC
DOCK1	FORWARD	GGAGGCAGAGGAGACGAACAG
	REVERSE	TTTGGCGAGGTATTTGTGCTTCC
NRIP1	FORWARD	TTACAGATGACAGCAGTGGTGATG
	REVERSE	CCAAGTGTTTAGCAAGGATTGAGTG
CALCRL	FORWARD	TTAGACATCCAGCAAGCAACAGAAC
	REVERSE	GCCAAGCGAGATAAGCAGTGATG

β-actin-F	Forward primer sequence [5'-3']:	CCTGGCACCCAGCACAAT
β-actin-R	Reverse primer sequence [5'-3']	GGGCCGGACTCGTCATAC

TableS7. The univariate cox regressin analysis of the PANoptosis related genes in HOVON cohort (training cohort). The genes with z_pvalue <0.05 were retained

Genes	beta	HR	z_pvalue	Wald_pvalue	Likelihood_pvalue
AKT1	-0.287869286	0.749859603	0.027169209	0.027169209	0.028387805
AKT3	0.159859997	1.173346588	0.004935433	0.004935433	0.005493956
AVEN	-0.291685226	0.747003636	0.006353062	0.006353062	0.006477747
C1QBP	-0.164046441	0.848702606	0.033762006	0.033762006	0.035011589
CYCS	-0.235875203	0.789879242	0.019882271	0.019882271	0.021929063
DAPK1	0.176171122	1.192642128	0.00066631	0.00066631	0.000595224
DBNL	-0.257058057	0.773323316	0.035923773	0.035923773	0.036547375
DFFB	-0.156630064	0.855020303	0.044159572	0.044159572	0.044950984
DSG2	0.356191499	1.42788096	0.000785623	0.000785623	0.00223535
MAGED1	0.196486586	1.217118994	0.003236861	0.003236861	0.00327327
OMA1	-0.131383571	0.876881364	0.048730669	0.048730669	0.05296001
PSMA1	0.434713211	1.544520044	0.0087957	0.0087957	0.008529317
PSMD5	-0.420772203	0.656539642	4.81E-05	4.81E-05	0.000182863
PTK2	0.449557163	1.567617832	0.040336751	0.040336751	0.048507253
SEPTIN4	0.612885701	1.845750003	0.005382232	0.005382232	0.006090051
STK24	-0.443965202	0.641487743	0.032094236	0.032094236	0.030461578
TJP2	0.130195852	1.139051447	0.001951272	0.001951272	0.001980228
TP53	-0.175869296	0.838727601	0.007292264	0.007292264	0.009299961
TP53BP2	-0.271863564	0.761958212	0.014682449	0.014682449	0.015312803
YWHAG	-0.204711984	0.814881982	0.014312417	0.014312417	0.016588278
CHMP2B	0.189934291	1.209170141	0.033216209	0.033216209	0.032100391
ELANE	-0.041646534	0.959208768	0.037217739	0.037217739	0.038216584
CAV1	-0.237007369	0.788985474	0.003521626	0.003521626	0.001660991
IPMK	-0.328709025	0.719852445	0.002370309	0.002370309	0.003132451
MAP3K5	0.1374139	1.147302919	0.041580048	0.041580048	0.038592538
PELI1	0.072298193	1.074975846	0.021459679	0.021459679	0.020434123
PYGL	-0.135776702	0.873037558	0.022195051	0.022195051	0.025344421

Table S8. The candidate genes were screened during the construction of the signature

The signature genes were screened with the variable selection using minimal depth via the R package "randomForestSRC". (n=19)

	depth	VIMP (variable importance).	relative importance
CALCRL	10.464	0.012734	1
NRIP1	12.178	0.007018	0.55111
DOCK1	12.63	0.007473	0.586859
GPRC5C	12.679	0.004454	0.349809
DNMT3B	12.79	0.005704	0.447968
ALDH2	12.889	0.004565	0.358468
NYNRIN	12.925	0.0051	0.400486
ETS2	12.963	0.006613	0.519353
LSP1	12.966	0.007062	0.554543
MAML1	13.008	0.006048	0.474923
PGRMC1	13.178	0.004835	0.379669
CLCN5	13.189	0.007439	0.584155
ADRM1	13.267	0.004774	0.374888
PDE4D	13.328	0.002971	0.233296
SPINT2	13.329	0.002945	0.2313
ETFB	13.358	0.003319	0.260625
SESN1	13.463	0.001768	0.138865
JAM3	13.474	0.003017	0.236894
IL2RA	13.476	0.003798	0.298236

Table S9. The coefficients of the signature genes in the developed predictive

Signatures	Coefficient
CALCRL	0.165201917
NRIP1	-0.045726056
DOCK1	0.000876038
GPRC5C	0.356917447
DNMT3B	0.2205022
ALDH2	0.164058052
NYNRIN	-0.044653997
ETS2	0.059592669
LSP1	0.013716468
MAML1	-0.432032192
PGRMC1	0.227522484
CLCN5	0.229219481
ADRM1	-0.203613382
PDE4D	0.270212408
SPINT2	0.15869172
ETFB	0.234474761
SESN1	0.125186518
JAM3	-0.001377123
IL2RA	0.113519502

Table S10. The objective remission rate (ORR) data for 21 cancers in TCGA and the correlation between the signature genes and the ORR

Gene	PearsonR	ValueP
NRIP1	-0.473	0.032
CALCRL	-0.287	0.141
JAM3	-0.269	0.157
MAML1	-0.222	0.204
GPRC5C	-0.188	0.242
DOCK1	-0.158	0.279
SPINT2	-0.154	0.285
NYNRIN	-0.138	0.305
SESN1	-0.135	0.309
DNMT3B	-0.106	0.348
PGRMC1	-0.072	0.395
ALDH2	0.01	0.514
PDE4D	0.025	0.536
LSP1	0.038	0.555
IL2RA	0.173	0.74
ETFB	0.245	0.82
ETS2	0.301	0.871
ADRM1	0.385	0.93
CLCN5	0.491	0.973
